# ﻿*Corrieoponenouragues* gen. nov., sp. nov., a new Ponerinae from French Guiana (Hymenoptera, Formicidae)

**DOI:** 10.3897/zookeys.1074.75551

**Published:** 2021-12-03

**Authors:** Flavia A. Esteves, Brian L. Fisher

**Affiliations:** 1 Entomology, California Academy of Sciences, 55 Music Concourse Drive, San Francisco, CA 94118, USA California Academy of Sciences San Francisco United States of America

**Keywords:** Ant Course, ants, myrmecology, neotropical, new genus, ponerines, South America

## Abstract

This study describes the worker and queen castes of the Neotropical ponerine *Corrieoponenouragues***gen. nov.**, **sp. nov.**, an ant from the tropical rainforest in French Guiana. Worker morphology of the taxon is compared with those of other Ponerinae and the similarities between them are discussed, refining the definition of character states for some diagnostic characters at the generic level, providing an identification key to the Neotropical genera, and making some adjustments to the taxonomic framework within the subfamily. Descriptions, diagnosis, character discussion, identiﬁcation key, and glossary are illustrated with more than 300 images and line drawings. Open science is supported by providing access to measurement data for specimens of the new genus, a matrix of character states for all ponerine taxa evaluated in this study, and specimen data for all examined material. The new or revived combinations presented here are *Pachycondylaprocidua* Emery, **comb. rev.**, *Neoponeracuriosa* (Mackay and Mackay), **comb. nov.**, *Leptogenysbutteli* (Forel), **comb. nov.**, and *Bothroponeraescherichi* (Forel), **comb. nov.** In addition, *Leptogenysbutteli* is synonymized with *Leptogenysmyops* (Emery), **syn. nov.**

## ﻿Introduction

Since 2001, 17 Ant Course editions have trained nearly 500 students from 59 countries, included more than 60 international instructors, and offered opportunities to explore the biological diversity in different parts of the globe, from Australia to Southeast Asia, to East Africa, and to North, Central, and South America (www.antcourse.org). Specimens collected in past editions enhanced our understanding of several aspects of ant biology, such as functional morphology (e.g., Peeters et al. 2017; Larabee et al. 2017, 2018; Gibson et al. 2018), ecology (Kronauer 2004), reproductive biology (Peeters 2017), and natural history (LaPolla et al. 2004; Lattke and Delsinne 2016). The course also created opportunities for remarkable discoveries. For example, two new genera records for USA fauna, *Typhlomyrmex* Mayr (CASENT0173317) and *Fulakora* Mann (MCZ-ENT00528501); the first record of the worker caste of the Afrotropical genus *Aenictogiton* Emery (CASENT0906052); and two genera recorded for the first time in Borneo (*Rhopalothrix* Mayr and *Tyrannomyrmex* Fernández; Fisher et al. 2015).

Here we describe a novel ponerine genus and species discovered during the 2018 Ant Course in the Natural Reserve of Nouragues, French Guiana. Ponerinae Lepeletier de Saint-Fargeau (see diagnosis in Fisher and Bolton 2016: 53–54) currently consists of 47 extant genera and 1,263 valid species (AntCat.org). Of these, 17 genera and 673 species are present in the Neotropics (AntWeb.org). Schmidt and Shattuck (2014) recently revised the higher classification of the subfamily and split the former genus *Pachycondyla* into 19 genera. The authors also described *Iroponeraodax*, a name coined by WL Brown Jr (Cornell University, Ithaca, USA), who unfortunately passed away before describing that unknown genus and species. *Iroponera* Schmidt & Shattuck is the most recent ponerine genus description based on a new species rather than reclassification.

As part of the process of diagnosing the new genus, we make a few adjustments to the taxonomic framework within Ponerinae, provide a new identification key for workers of the Neotropical genera, and reassess morphological characters across the subfamily.

## ﻿Materials and methods

### ﻿Study site

**Figure 1.**
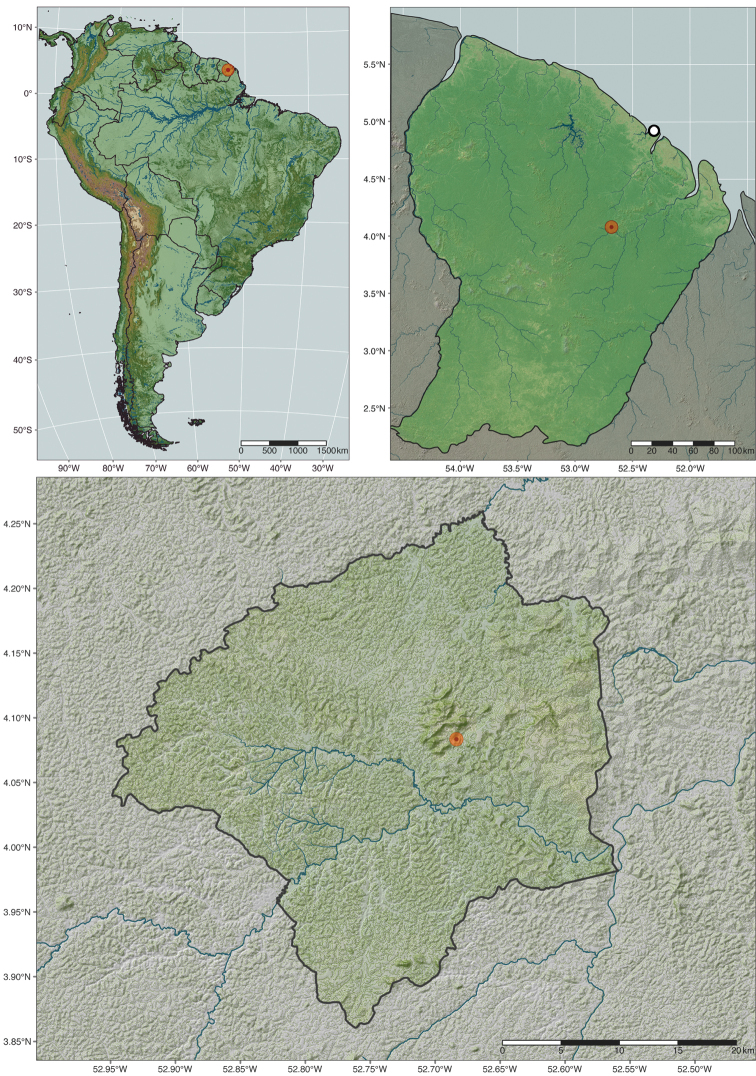
Shaded relief maps of the type locality of *Corrieoponenouragues.* Clockwise from top-left: South America, French Guiana, and Natural Reserve of Nouragues. Symbols: red circle, collection locality; circle with white fill and black outline, Cayenne, the capital of French Guiana.

**Figure 2.**
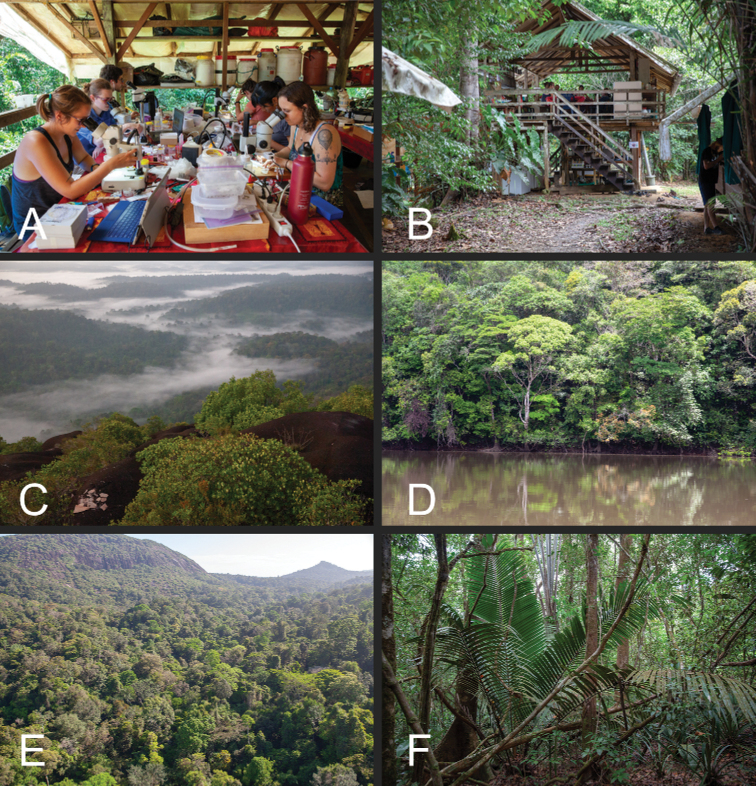
Images of the Nouragues Research Station **A** ant Course 2018 students working in the lab at the Inselberg camp **B** view of the Inselberg camp kitchen facilities **C** forested landscape of the reserve viewed from the Nouragues Inselberg **D** rainforest along the Approuague river **E** the Nouragues Inselberg (top left of the image) and surrounding landscape **F** collection site of *Corrieoponenouragues*. Photography by BL Fisher.

Ant Course 2018 was held in August at Nouragues Research Station (4.08796°N, 52.68002°W) in the Natural Reserve of Nouragues in French Guiana (Figs 1, 2). A French overseas territory on the northeastern edge of South America, French Guiana sits on a Precambrian massif known as the Guiana Shield (Grimaldi and Riéra 2001). The Nouragues station’s hilly landscape features a granitic inselberg that rises to 430 m (Fig. 2C, E; Grimaldi and Riéra 2001). The station lies in the Guianan Lowland Moist Forests ecoregion, a subset of the Tropical and Subtropical Moist Forests biome (Dinerstein et al. 2017); the climate is equatorial humid, and seasonality is determined by the movements of the Inter-tropical Convergence Zone (Grimaldi and Riéra 2001). The region experiences an average annual temperature of ~26 °C and yearly average precipitation ~2990 mm, with the rainy season occurring from November to August but interrupted in March by a short dry period (Grimaldi and Riéra 2001).

### ﻿Material examined and specimen records

During this study, we contrasted the morphology of the new genus with 129 species or subspecies representing all 47 currently valid extant Ponerinae genera (AntCat 2021; see Table 1 for taxa list, Suppl. material 1: Table S1 for specimen data). Suppl. material 3: Table S3 (character matrix) documents characters evaluated that could be unambiguously discretized, and we share it here to disclose our methodology and foster validation, replication, and reinterpretation of our results (see also Suppl. material 2: Table S2 for character statements).

**Table 1.** List of taxa used for direct comparisons with the morphology of the new genus. In addition, it contains information on how specimens were determined.

Taxon name and Author(s)year	Determination methodology
*Anochetusangolensis* Brown	Type examined
*Anochetusemarginatus* (Fabricius)	Det. CA Schmidt; compared with type images
*Asphinctoponedifferens* Bolton & Fisher	Type examined
*Asphinctoponesilvestrii* Santschi	Det. BL Fisher
*Austroponeracastanea* (Mayr)	Det. WL Brown
*Belonopeltadeletrix* Mann	Det. SP Cover; FA Esteves; BL Fisher
*Boloponeraikemkha* Hawkes	Type examined
*Boloponeravicans* Fisher	Type examined
*Bothroponeracariosa* Emery	Det. BL Fisher
*Bothroponeracrassa* (Emery)	Det. G Fischer; BL Fisher
*Bothroponerapachyderma* (Emery)	Det. BL Fisher; compared with type images
*Bothroponerasilvestrii* (Santschi)	Det. G Fischer; compared with type images
*Bothroponeratalpa* André	Det. BL Fisher
*Brachyponerachinensis* (Emery)	Det. WP Mackay; compared with type images
*Brachyponeracroceicornis* (Emery)	Type examined
*Brachyponeralutea* (Mayr)	Det. BL Fisher; compared with type images
*Brachyponeraluteipes* (Mayr)	Det. WL Brown; compared with type images
*Brachyponeraobscurans* (Walker)	Det. BL Fisher
*Brachyponerasennaarensis* (Mayr)	Det. FA Esteves; compared with type images
*Buniaponeamblyops* (Emery)	Det. FA Esteves; compared with type images
*Centromyrmexbrachycola* (Roger)	Det. ES Ross; compared with type images
*Centromyrmexdecessor* Bolton & Fisher	Type examined
*Centromyrmexereptor* Bolton & Fisher	Type examined
*Centromyrmexraptor* Bolton & Fisher	Type examined
*Cryptoponegilva* (Roger)	Det. BL Fisher; J Lattke; compared with type images
*Cryptoponeguianensis* (Weber)	Det. SP Cover; PS Ward
*Cryptoponehartwigi* Arnold	Det. P Hawkes
*Diacammaceylonense* Emery	Det. C Peeters
*Dinoponeralongipes* Emery	Det. PA Lenhart
*Dinoponeralucida* Emery	Det. JRM Santos
*Dolioponerafustigera* Brown	Det. BL Fisher; compared with type images
*Ectomomyrmexjavanus* Mayr	Det. WL Brown
*Emeryoponebuttelreepeni* Forel	Det. D Agosti; FA Esteves
*Euponerabrunoi* (Forel)	Det. BL Fisher; compared with type images
*Euponerasikorae* (Forel)	Det. BL Fisher
*Euponerasjostedti* (Mayr)	Det. BL Fisher; RR Snelling
*Feroponeraferox* Bolton & Fisher	Type examined
*Fisheroponeambigua* (Weber)	Det. FA Esteves; BL Fisher
*Hagensiahavilandimarleyi* (Arnold)	Det. C Peeters; compared with type images
*Harpegnathossaltator* Jerdon	Det. C Peeters
*Hypoponerapunctatissima* (Roger)	Det. BL Fisher
*Iroponeraodax* Schmidt & Shattuck	Det. WL Brown
*Leptogenysixta* Lattke	Type examined
*Leptogenysperuana* Lattke	Type examined
*Leptogenyspodenzanai* (Emery)	Det. RA Keller (?); compared with type images
*Leptogenyspucuna* Lattke	Type examined
*Leptogenyssonora* Lattke	Type examined
*Leptogenyswheeleri* Forel	Det. FA Esteves; compared with type images
*Loboponeraobeliscata* Bolton & Brown	Det. taxon author(s)
*Loboponeravigilans* Bolton & Brown	Det. taxon author(s)
*Mayaponeraarhuaca* (Forel)	Det. WL Brown; compared with type images
*Mayaponerabecculata* (Mackay & Mackay)	Type examined
*Mayaponeracernua* (Mackay & Mackay)	Type examined
*Mayaponeraconicula* (Mackay & Mackay)	Type examined
*Mayaponeraconstricta* (Mayr)	Det. JT Longino
*Mayaponerapergandei* (Forel)	Det. WP Mackay
*Megaponeraanalis* (Latreille)	Det. FA Esteves
*Mesoponeraambigua* (André)	Det. G Fischer; BL Fisher
*Mesoponeraaustralis* (Forel)	Det. FA Esteves
*Mesoponeracaffraria* (Smith)	Det. BL Fisher
*Mesoponeraelisaerotundata* (Emery)	Det. G Fischer
*Mesoponeramelanariamacra* (Emery)	Det. FA Esteves
*Mesoponerapapuana* (Viehmeyer)	Type examined
*Mesoponerarubra* (Smith)	Det. FA Esteves; compared with type images
*Mesoponerasubiridescens* (Wheeler)	Det. G Fischer; BL Fisher
*Myopiasdarioi* Probst & Boudinot	Type examined
*Myopiasmaligna* (Smith)	Det. FA Esteves; compared with type images
*Neoponeraaenescens* (Mayr)	Det. WL Brown; compared with type images
*Neoponeraapicalis* (Latreille)	Det. WP Mackay; compared with type images
*Neoponerabugabensis* (Forel)	Det. WP Mackay; compared with type images
*Neoponeracarinulata* (Roger)	Det. WP Mackay; compared with type images
*Neoponeracavinodis* Mann	Det. WL Brown
*Neoponeracommutata* (Roger)	Det. WL Brown
*Neoponeracrenata* (Roger)	Det. WP Mackay; compared with type images
*Neoponeradismarginata* (Mackay & Mackay)	Det. taxon author(s)
*Neoponeraeleonorae* (Forel)	Det. WL Brown
Neoponeracf.fiebrigi	Det. BL Fisher
*Neoponerafisheri* (Mackay & Mackay)	Type examined
*Neoponerafoetida* (Linnaeus)	Det. WP Mackay
*Neoponeraglobularia* (Mackay & Mackay)	Type examined
*Neoponerainsignis* (Mackay & Mackay)	Det. BL Fisher
*Neoponerainversa* (Smith)	Det. WP Mackay; compared with type images
*Neoponeralaevigata* (Smith)	Det. WP Mackay; compared with type images
*Neoponeraluteola* (Roger)	Det. WP Mackay; compared with type images
*Neoponeramoesta* (Mayr)	Det. WP Mackay; compared with type images
*Neoponeraobscuricornis* (Emery)	Det. WP Mackay; compared with type images
*Neoponeraschoedli* (Mackay & Mackay)	Type examined
*Neoponerastriatinodis* (Emery)	Det. WP Mackay
*Neoponeraunidentata* (Mayr)	Det. BL Fisher; compared with type images
*Neoponeraverenae* (Forel)	Det. WP Mackay; compared with type images
*Neoponeravillosa* (Fabricius)	Det. LR Davis; compared with type images
*Odontomachusbauri* Emery	Det. FA Esteves; RA Keller
*Odontoponeratransversa* (Smith)	Det. FA Esteves; RA Keller
*Ophthalmoponeberthoudi* Forel	Det. FA Esteves; compared with type images
*Pachycondylacrassinoda* (Latreille)	Det FA Esteves, Kempf (1964) ID key
*Pachycondylaharpax* (Fabricius)	Det FA Esteves, Kempf (1964) ID key; compared with type images
*Pachycondylaimpressa* (Roger)	Det FA Esteves, Kempf (1964) ID key; compared with type images
*Pachycondylalattkei* Mackay & Mackay	Type examined
*Pachycondylalenis* Kempf	Det FA Esteves, Kempf (1964) ID key
*Pachycondylaprocidua* Emery	Det FA Esteves, Kempf (1964) ID key; compared with type images
*Pachycondylastriata* Smith	Det. WP Mackay; compared with type images
*Paltothyreustarsatus* (Fabricius)	Det. G Alpert; G Fischer
*Parvaponeradarwiniimadecassa* (Emery)	Det. BL Fisher
*Phrynoponerapulchella* Bolton & Fisher	Type examined
*Phrynoponeratransversa* Bolton & Fisher	Type examined
*Platythyreacribrinodis* (Gerstäcker)	Det. FA Esteves, Brown (1975) ID key; compared with type images
*Platythyreapunctata* (Smith)	Det. LR Davis; RA Keller; compared with type images
*Platythyreaturneri* Forel	Det. RA Keller (?); CA Schmidt; PS Ward
*Plectroctenastrigosa* Emery	Det. C Peeters; HJ Robertson
*Poneraalpha* Taylor	Det. taxon author(s)
*Ponerapennsylvanica* Buckley	Det. RA Keller; J Lattke
*Promyopiassilvestrii* (Santschi)	Det. B Bolton
*Psalidomyrmexprocerus* Emery	Det. WL Brown; A Dejean; BL Fisher
*Pseudoneoponeraporcata* (Emery)	Det. RA Keller
*Pseudoneoponeratridentata* (Smith)	Det. BL Fisher
*Pseudoponeragilberti* (Kempf)	Det. FA Esteves, Mackay and Mackay (2010) ID key; compared with Kempf (1960) description
*Pseudoponerastigma* (Fabricius)	Type examined
*Rasoponecostaricensis* Longino & Branstetter	Type examined
*Rasoponecryptergates* Longino & Branstetter	Type examined
*Rasoponecubitalis* Longino & Branstetter	Type examined
*Rasoponeguatemalensis* Longino & Branstetter	Type examined
*Rasoponepanamensis* (Forel)	Type examined
*Rasoponepluviselva* Longino & Branstetter	Type examined
*Rasoponepolitognatha* Longino & Branstetter	Type examined
*Simopeltaoculata* Gotwald & Brown	Type examined
*Simopeltatransversa* Mackay & Mackay	Type examined
*Streblognathuspeetersi* Robertson	Det. C Peeters
*Thaumatomyrmexfraxini* D’Esquivel & Jahyny	Det. taxon author(s)
*Thaumatomyrmexzeteki* Smith	Det. FA Esteves, Kempf (1975) ID key

Specimens were examined with a Leica M125 microscope with 187.5 × total magnification power (Leica Microsystems, Switzerland). Minute characters were accessed with scanning electron microscopy (SEM) or through SEM images previously available on AntWeb (www.antweb.org). Each specimen evaluated bears a registered unique identifier (e.g., CASENT0830464) associated with collection and specimen information, images, and identification on AntWeb. Data is accessible on AntWeb through the persistent URL of a given unique identifier (e.g., www.antweb.org/specimen/CASENT0830464).

Maxillary and labial palpal counts are based on direct observation of specimens with protracted maxillolabial complex (*N* = 41), dissections (*N* = 47), and existing SEM images of the maxillolabial complex on AntWeb.org (*N* = 26). For dissected specimens, the maxillolabial complex was removed from the buccal cavity with a pin or forceps. Larger specimens were observed with a Leica M125 microscope; smaller specimens were accessed with SEM. See Suppl. material 3: Table S3 (column Notes_char_9) for specimen data on palps evaluated, including unique specimen identifiers and observation method.

In subsequent sections, we refer to several other species whose morphology was only assessed through extended-focus images of specimens databased on AntWeb. Those species were not included in Table 1 or Tables S1 and S3. Instead, and when necessary, references are accompanied by the unique identifier of relevant specimens, which are hyperlinked to respective webpages on AntWeb.

### ﻿Images, drawings, and maps

Extended focus montage images were acquired with a Leica DFC 425 camera and LEICA APPLICATION SUITE software (version 3.8; Leica Microsystems, Switzerland). For most SEM images, samples were coated with gold-palladium in a Cressington 108 Sputter Coater (Cressington Scientific Instruments, United Kingdom), and micrographs were taken at high vacuum secondary electron emission (accelerating voltage: 15 kV, spot intensity diameter: 40) in a Hitachi SU3500 microscope (Hitachi High-Technologies, Japan). Uncoated specimens (e.g., primary types, unique specimens) were also imaged at high vacuum secondary electron emission, but with the accelerating voltage set to 1.5 kV and spot intensity to 50. Image enhancement (e.g., contrast, levels, sharpness, darken background) occurred in ADOBE PHOTOSHOP (version 22.4.2; Adobe Inc., United States of America). All images produced in this study are available on AntWeb.

Line drawings were originally traced in ADOBE ILLUSTRATOR (version 25.2.3; Adobe Inc., United States of America) or modified from artwork produced by scientific illustrator Jessica Huppi, whose authorship is credited in figure captions when pertinent. Jessica Huppi line art is part of a work-for-hire agreement that makes the California Academy of Sciences a copyright holder for her artwork.

Mapping of the study area occurred on RStudio Desktop (version 1.4.1717; RStudio Team 2021), an integrated development environment for R (version 4.1.0; R Core Team 2021). Spatial data was sourced directly from the following: NASADEM HGT v001 products (NASA JPL 2020), Global Lakes and Wetlands Database (GLWD-2 dataset; Lehner and Döll 2004), Shuttle Radar Topography Mission Water Body models (NASA JPL 2013), Global River Classification dataset (GloRiC version 1.0, Ouellet Dallaire et al. 2018), and Global Tree Cover dataset (Hansen et al. 2013). Alternatively, data were acquired and imported into R with functions of the following R packages: OSMDATA (Padgham et al. 2017), RASTER (Hijmans 2021), RWORLDMAP (South 2011), and RWORLDXTRA (South 2012); the OSMDATA package imports features from OpenStreetMap (OpenStreetMap.org data were available under the Open Database License). Data geoprocessing was executed with functions of DPLYR (Wickham et al. 2021), GDALUTILS (Greenberg and Mattiuzzi 2020), RASTER, and SF (Pebesma 2018) packages. Map projection and mapping were performed with functions available in the TMAP package (Tennekes 2018).

### ﻿Terminology

**Figure 3.**
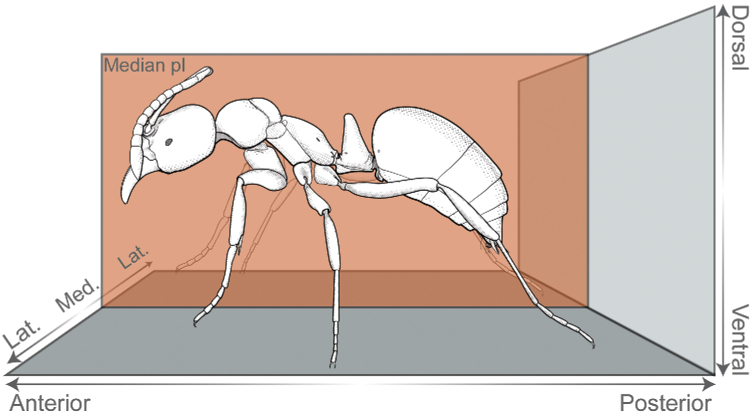
Anatomical position and coordinate system adopted as framework for positional and directional terminology. *Corrieoponenouragues* is shown in profile, with the head oriented to the left. The coordinate system is defined by the median plane and three directional axes (anteroposterior, dorsoventral, and mediolateral). Illustration by FA Esteves.

Positional and directional terminology references a hypothetical worker seen in profile, with the head oriented to the left and standing with legs slightly spread over the horizontal plane of a multidimensional space (Fig. 3). Mouthparts are retracted, and antennal scapes are directed posteriad, parallel to the head dorsum. We defined three main directional axes from this orientation, which guided the description of direction and relative position of morphological characters in this study. The anteroposterior axis extends horizontally across the space, from the anterior left side to the posterior right; the dorsoventral axis extends vertically, with “dorsal” directed upwards and “ventral” directed downwards. Finally, the mediolateral axis is perpendicular to a median plane that bisects the ant through its bilateral line, with “medial” directed towards the plane and "lateral" diverging from it (Fig. 3).

**Figure 4.**
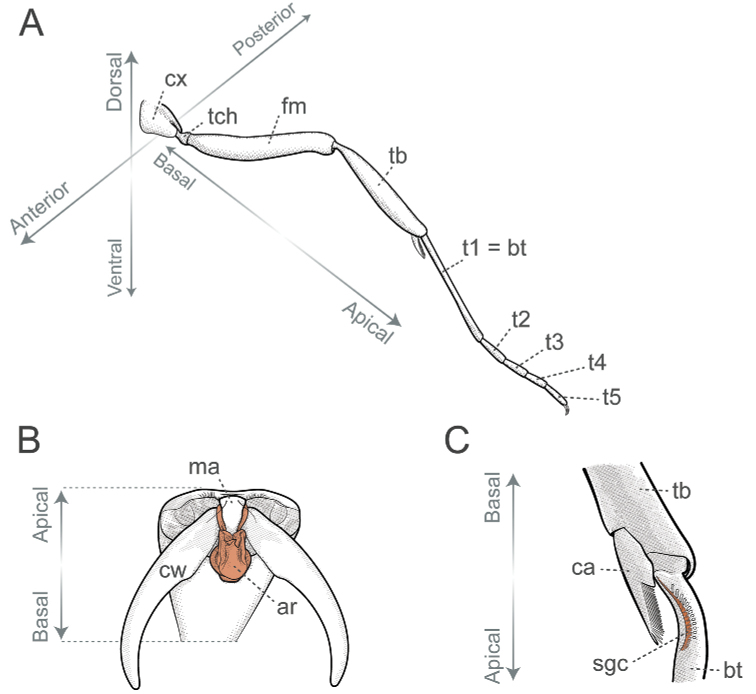
Glossary of terminology, part I **A** hypothetical hindleg in a three-dimensional coordinate system defined by the anteroposterior, dorsoventral, and basoapical directional axes **B** apicalmost tarsomere and pretarsus in ventroapical view, with the retracted arolium highlighted in orange **C** probasitarsus and protibial apex in posterior view, with the comb of strigil highlighted in orange. Abbreviations: **ar**, arolium; **bt**, basitarsus; **ca**, calcar of strigil; **cx**, coxa; **cw**, claw; **fm**, femur; **ma**, manubrium; **sgc**, comb of strigil; **tb**, tibia; **t1–5**, tarsi; **tch**, trochanter. Illustrations by FA Esteves.

Additionally, we utilized a fourth axis for aiding the description of positions and directions of characters located on appendages, sclerites, processes, or any structure that may project away from the body. The basoapical axis arises from the median plane, with “basal” being close to the plane while “apical” is distant from it (Fig. 4A). Morphological terminology is based on Keller (2011) unless otherwise stated. Wherever possible, terms in the glossary are hyperlinked to correspondent terms in the Hymenoptera Anatomy Ontology portal (Yoder et al. 2010). Terms absent from the glossary below are defined, illustrated, or associated with references to pertinent literature at first use.

**Figure 5.**
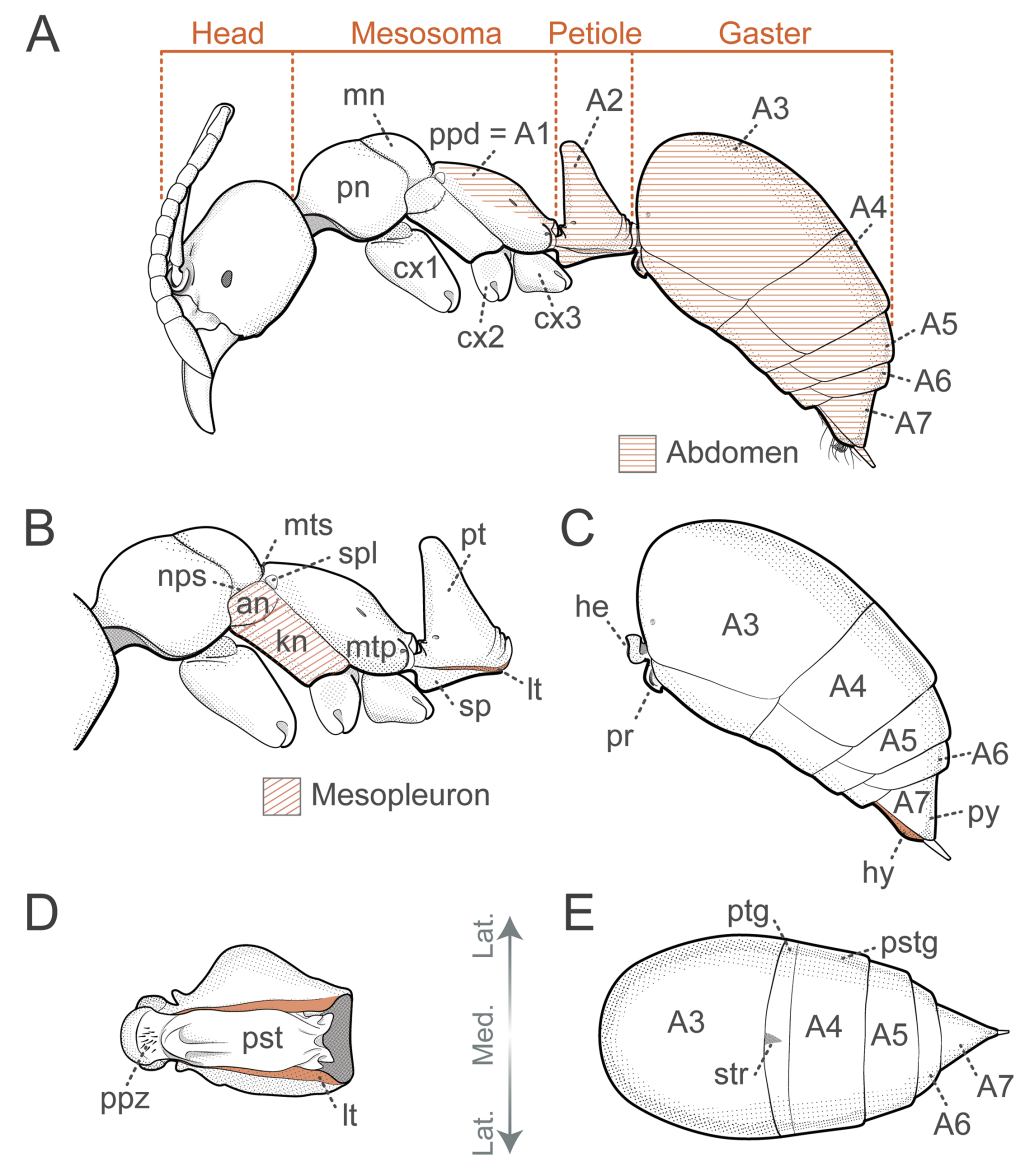
Glossary of terminology, part II **A** body in profile, with the abdomen highlighted in orange hatching **B** mesosoma and petiole in profile, with the mesopleuron highlighted in orange hatching; laterotergite in solid orange **C** gaster in profile, with the hypopygium highlighted in orange **D** ventral face of the petiole; laterotergite highlighted in orange **E** dorsal face of the gaster. Abbreviations: **A1–7**, abdominal segments; **an**, anepisternum; **cx1–3**, coxae; **he**, helcium; **hy**, hypopygium; **kn**, katepisternum; **lt**, laterotergite; **mn**, mesonotum; **mtp**, metapleuron; **mts**, metanotal sulcus; **nps**, notopleural suture; **pn**, pronotum; **ppd**, propodeum; **ppz**, petiolar proprioceptor zone; **pr**, prora; **pst**, petiolar sternite; **pstg**, posttergite; **pt**, petiole; **ptg**, pretergite; **py**, pygidium; **sp**, subpetiolar process; **spl**, spiracular lobe; **str**, stridulitrum. Illustrations by FA Esteves.

**Anepisternum** (an, Fig. 5B): Dorsal subdivision of the mesopleuron, separated from the katepisternum by the mesepisternal sulcus (= anapleural sulcus in Fisher and Bolton 2016).

**Figure 6.**
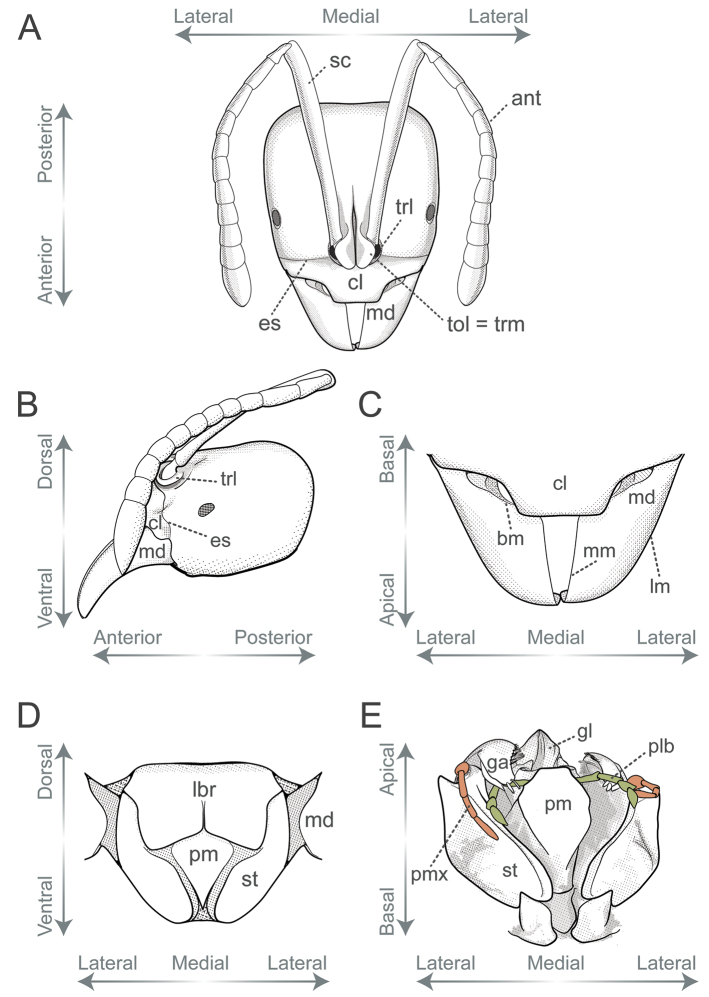
Glossary of terminology, part III **A** head in full-face view **B** head in lateral view **C** mandibles and the anterior area of the clypeus in dorsal view **D** labrum and retracted maxillolabial complex in ventral view **E** maxillolabial complex in central view; labial palps highlighted in green, maxillary palps in orange. Abbreviations: **ant**, antenna; **bm**, basal margin of the mandible; **cl**, clypeus; **es**, epistomal sulcus; **ga**, galea; **gl**, glossa; **lbr**, labrum; **lm**, lateral margin of the mandible; **md**, mandible; **mm**, masticatory margin of the mandible; **plb**, labial palps; **pm**, premental shield; **pmx**, maxillary palps; **sc**, scape; **st**, stipes; **tol**, torular lobe; **trm**, torular median arch. Illustrations by FA Esteves (**A, B, C, E**), and modified from artwork by Jessica Huppi (**D**).

**Antenna** (pl antennae; ant, Fig. 6A): Paired head appendage that, from base to apex, is composed of scape, pedicel, and flagellum. We refer to pedicel and flagellomeres as antennomeres; Roman numerals in ascending order indicate the position of individual antennomeres along the antenna’s basoapical axis (e.g., scape = antennomere I, pedicel = antennomere II, basalmost flagellomere = antennomere III).

**Arolium** (pl arolia; ar, Fig. 4B): The adhesive pretarsal organ; a soft cuticular structure between the pretarsal claws.

**Bulbus neck**: The constricted portion of the antennal scape, bordered basally by the bulbus (= condylar bulbus in Fisher and Bolton 2016) and apically by the main shaft of the scape.

**Calcar of strigil** (ca, Fig. 4C): The protibial spur; together with the comb of strigil, forms the antennal cleaning organ.

**Comb of strigil** (sgc, Fig. 4C): Comb-like structure on the ventral face of the basal portion of the probasitarsus; together with the calcar of strigil, forms the antennal cleaning organ.

**Epistomal sulcus** (es, Fig. 6A, B; as in Richards 1977): Sulcus delimitating the clypeus posteriorly and laterally from the remainder of the head.

**Galea** (ga, Fig. 6E): The spatulate lobe located at the apical part of the stipes; part of the maxilla.

**Galeal comb**: Row of setae located on the outer face of the medial margin of the galea, opposite the maxillary comb located near the inner face of the margin.

**Galeal crown**: The apicalmost part of the galea.

**Gaster** (Fig. 5A, C, E; as in Fisher and Bolton 2016): Tagma formed by the third, fourth, fifth, sixth, and seventh abdominal segments in female ponerines. When mentioning individual segments, we refer to the homologous abdominal segment labeled by a corresponding Roman numeral (e.g., abdominal segment V). For aesthetic reasons in figures and measurements abbreviations, segments are noted by “A” followed by the corresponding Arabic numeral (e.g., A5).

**Helcium** (he, Fig. 5C): Structure formed by the specialized presclerites of abdominal segment III, which articulates with the petiole.

**Hypopygium** (hy, Fig. 5C): The sternite of abdominal segment VII in adult female ants.

**Katepisternum** (kn, Fig. 5B): Ventral subdivision of the mesopleuron, separated from the anepisternum by the mesepisternal sulcus (= anapleural sulcus in Fisher and Bolton 2016). Note that this usage does not claim homology between the katepisternum in ants and homonymous mesothoracic areas in other insects.

**Labrum** (lbr, Fig. 6D): Mouthpart appendage that connects to the anterior margin, or the ventral face of the clypeus, and usually folds over the retracted maxillolabial complex.

**Labial palps** (plb, Fig. 6E): Labial ring-shaped sclerites articulated with the apicolateral portion of the prementum. We refer to each sclerite as a palpomere and use Roman numerals in ascending order to indicate the position of individual palpomeres along the basoapical axis of the palps (e.g., basalmost labial palpomere = labial palpomere I).

**Lower and upper metapleuron** (as in Snodgrass 1910): Secondary division of the metapleuron into dorsal wing-bearing and ventral leg-bearing portions.

**Maxillary palps** (pmx, Fig. 6E): Ring-shaped sclerites articulated with the apicomedial or the apicalmost portion of the stipes in the maxilla. We refer to each sclerite as a palpomere and use Roman numerals in ascending order to indicate the position of individual palpomeres along the basoapical axis of the palps (e.g., basalmost maxillary palpomere = maxillary palpomere I).

**Mesoscutellar-axillar complex** (as in Gibson et al. 1998): In winged ants, the area of the mesonotum that comprises the mesoscutellum and axillae; located posteriad to the transscutal line.

**Mesoscutum** (as in Gibson et al. 1998): In winged ants, the region of the mesonotum anterior to the transscutal line and mesoscutellar-axillar complex, whose laterals usually bear parapsidal lines.

**Mesometapleural suture** (as in Fisher and Bolton 2016): Groove-like suture delimiting the mesopleuron from the metapleuron.

**Mesonotum** (Fig. 5A): The tergum of the mesothorax.

**Mesopleuron** (Fig. 5B; as in Fisher and Bolton 2016): Pleuron of the mesothorax; extends over the lateral and most of the ventral external surfaces of the second thoracic segment.

**Mesosoma** (Fig. 5A): Tagma formed by the three thoracic segments (pro-, meso-, and metathorax) plus the propodeum (abdominal segment I).

**Mesosternal process** (as in Fisher and Bolton 2016): The pair of cuticular projections surrounding the midline of the mesothorax’s ventral face, anterior to the mesocoxal cavities.

**Metasternal process** (as in Fisher and Bolton 2016): The pair of cuticular projections surrounding the midline of the metathorax’s ventral face, anterior to the metacoxal cavities.

**Metanotum** (mts, Fig. 5B): The tergum of the metathorax; reduced to a groove (Fig. 5B) or completely indistinct in Ponerinae workers.

**Metapleuron** (mtp, Fig. 5B; as in Fisher and Bolton 2016): Pleuron of the metathorax; extends over the lateral and ventral external surfaces of the third thoracic segment; bears the metapleural gland opening laterally.

**Notopleural suture** (nps, Fig. 5B): Suture between mesopleuron and mesonotum.

**Petiolar laterotergite** (lt, Fig. 5B, D): Paired, long, narrow, strip-like area of the ventral margin of the petiolar tergite that is separated from the main part of the sclerite by an impression; flanks the petiolar sternite.

**Petiolar proprioceptor zone** (ppz, Fig. 5D): A depression on the anteriormost part of the petiolar sternite, sharply delineated anteriorly, and bearing numerous sensilla.

**Petiole** (pt, Fig. 5A, B, D): Abdominal segment II.

**Postsclerite**: Posterior portion of each abdominal sclerite not concealed by an articulation. The term posttergite refers to the postsclerite of a tergum (pstg, Fig. 5E), and poststernite to the postsclerite of a sternum (Fig. 5D).

**Presclerite**: Anterior articulatory region of each abdominal sclerite overlapped by the anterior segment. The term pretergite refers to the presclerite of a tergum (ptg, Fig. 5E), and presternite to the presclerite of a sternum.

**Premental shield** (pm, Fig. 6D, E): Labial sclerite that articulates apicolaterally with the labial palps.

**Pronotum** (pn, Fig. 5A): The tergum of the prothorax, which is hypertrophied in workers, and also in queens and males of some ant taxa.

**Prora** (pr, Fig. 5C; modified from Boudinot et al. 2020): Anteroventral process of abdominal sternite III that contacts the petiole sternite and gives stability to the ventral flexion of the gaster during stinging. It may be located anywhere from the area between the ventral margins of the helcial tergite arch to the anterior face of the poststernite.

**Pygidium** (py, Fig. 5A, C): In adult female ants, the tergite of abdominal segment VII.

**Scape** (sc, Fig. 6A): Basalmost antennomere followed apically by the pedicel; formed by the bulbus, the bulbus neck, and the main shaft of the scape.

**Scutoscutellar sulcus** (as in Gibson 1985): Sulcus impressed along the scutoscutellar suture. The scutoscutellar suture runs across the mesoscutellar-axillar complex, and separates axillae from mesoscutellum.

**Stipes** (st, Fig. 6D, E): Maxillary sclerite that articulates apically with the maxillary palps and with the galea.

**Subpetiolar process** (sp, Fig. 5B; as in Fisher and Bolton 2016): Ventral projection of the petiolar poststernite.

**Suture and sulcus**: A suture is a groove formed by the fusion of two sclerites; sulcus is an impression that corresponds to an apodeme.

**Torular lobes** (tol, Fig. 6A): Trait formed by the laterally projected median arches of the torulus; similar to frontal lobes.

Additionally, we used the following adjectives to describe vestiture:

**Aristate**: Shaped basally like a spine-like seta, and bearing a long, thin, flexuous apex.

**Figure 7.**
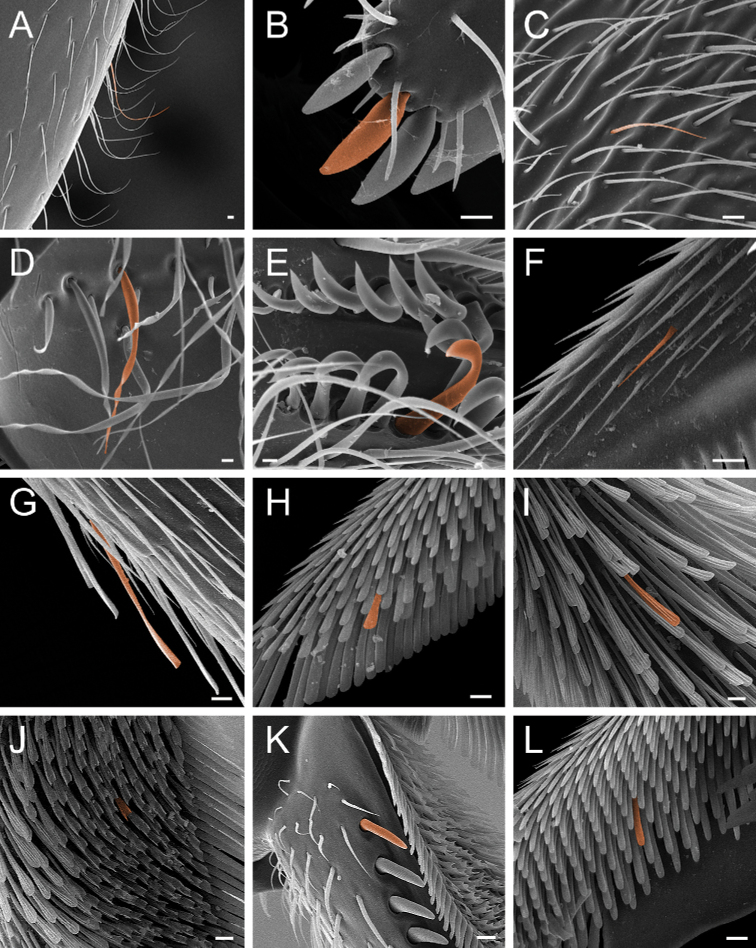
Glossary of terminology, part IV: vestiture. Individual parts show SEM images taken from *Corrieoponenouragues*; paratypes, worker caste. Distinct types of seta and microtrichium are highlighted in orange **A** buoyant seta **B** elliptic seta **C** filiform seta **D** helicoid seta **E** hook-shaped seta **F** lanceolate microtrichium **G** spatulate seta **H** spatulate microtrichium **I** spatulate-costate seta **J** spatulate-bicuspid seta **K** stout, spine-like seta **L** tubiform microtrichium. Specimens imaged: CASENT0872031 (**A, C, D, K**) and CASENT0923158 (**B, E–J, L**). Images by FA Esteves; available at AntWeb.org. Scale bars: 0.01 mm.

**Buoyant** (Fig. 7A): Shaped as if floating in air and weightless.

**Elliptic** (Fig. 7B): Elongate oval, with the widest part near the middle.

**Filiform** (Fig. 7C): Filamentous, shaped like a thread. Here used to describe seta with a regular, hair-like shape.

**Glabrous**: Devoid of hair or cuticular projections.

**Helicoid** (Fig. 7D): Seta compressed longitudinally and twisted, resembling a helix.

**Hook-shaped** (Fig. 7E): Seta with the apical portion curved like a hook.

**Lanceolate** (Fig. 7F): Shaped like a lancehead, with the widest part near the base and the narrowest part near the apex.

**Microtrichium** (pl microtrichia; Fig. 7F, H, L): Setae-like, minute cuticular projection.

**Serrate**: With one margin bearing a series of small, sharp, teeth-like projections.

**Spatulate** (Fig. 7G, H): Shaped like a spatula, with a broad, flat apex that tapers to the base.

**Spatulate-costate** (Fig. 7I): Spatulate seta with longitudinal parallel ridges.

**Spatulate-bicuspid** (Fig. 7J): Spatulate seta in which the apex is much wider than the base, so that its apicolateral corners arch over the apicomedial area, giving the seta a bicuspid appearance.

**Stout** (Fig. 7K): Heavily built.

**Tubiform** (Fig. 7L): Tubular, not tapering to a point.

Sculpture terminology follows Harris (1979), as below.

**Figure 8.**
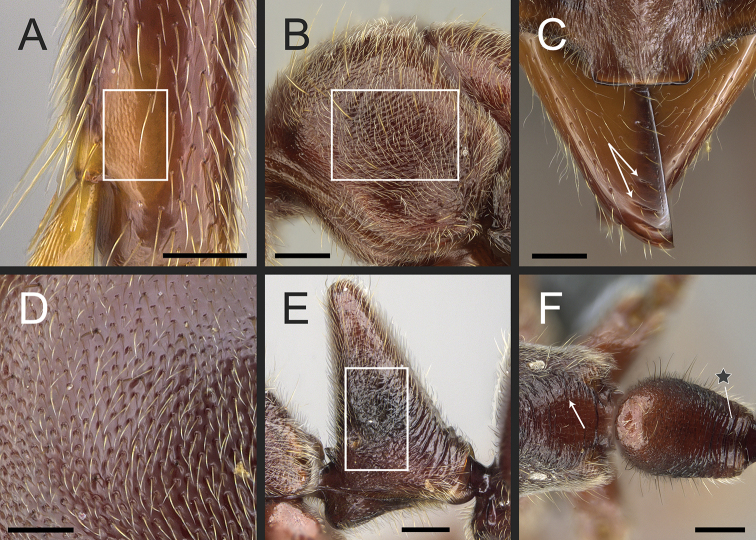
Glossary of terminology, part V: sculpture. Individual parts show SEM images taken from *Corrieoponenouragues*; holotype, worker (CASENT0830464) **A** rectangle encloses the colliculate sculpture on the metatibial apicoposterior surface **B** rectangle encloses the costulate lateral surface of the pronotum **C** arrows indicate the fossulae on the dorsal face of the mandible **D** punctate sculpture on the dorsoposterior area of the head **E** rectangle encloses the confused rugose sculpture on the petiolar profile **F** declivitous face of the propodeum and petiole in dorsal view; arrow indicates the strigulate sculpture; star highlights the strigate sculpture. Images by M Esposito (**A, E**) and FA Esteves (**B–D, F**); available at AntWeb.org. Scale bars: 0.1 mm (**A, D**); 0.2 mm (**B, C, E, F**).

**Colliculate** (Fig. 8A): Covered with continuous, short, rounded prominences.

**Confused** (Fig. 8E): Sculpture without definite outlines or definite pattern.

**Costulate** (Fig. 8B): With thin, longitudinal, parallel ridges.

**Fossula** (pl fossulae; Fig. 8C): Elongate, oblong depressions.

**Punctate** (Fig. 8D): With fine, impressed punctures.

**Rugose** (Fig. 8E): Wrinkled.

**Smooth**: Devoid of any sculpturing.

**Strigate** (Fig. 8F): With narrow, transverse ridges.

**Strigulate** (Fig. 8F): Finely or minutely strigate.

### ﻿Measurements and indices

We utilized linear morphometry to quantify size and offer a means of comparison with other Ponerinae taxa. Measurements, indices, and abbreviations follow Fisher (2006). All measurements were taken at 80 × power with a Leica MZ125 microscope using an orthogonal pair of micrometers, recorded to the nearest 0.001 mm, rounded to two decimal places, and presented as minimum and maximum values with holotype measurement within parentheses. Indices are rounded to the nearest integer and expressed as minimum and maximum values with holotype index within parentheses (see original data in Suppl. material 4: Table S4).

**Figure 9.**
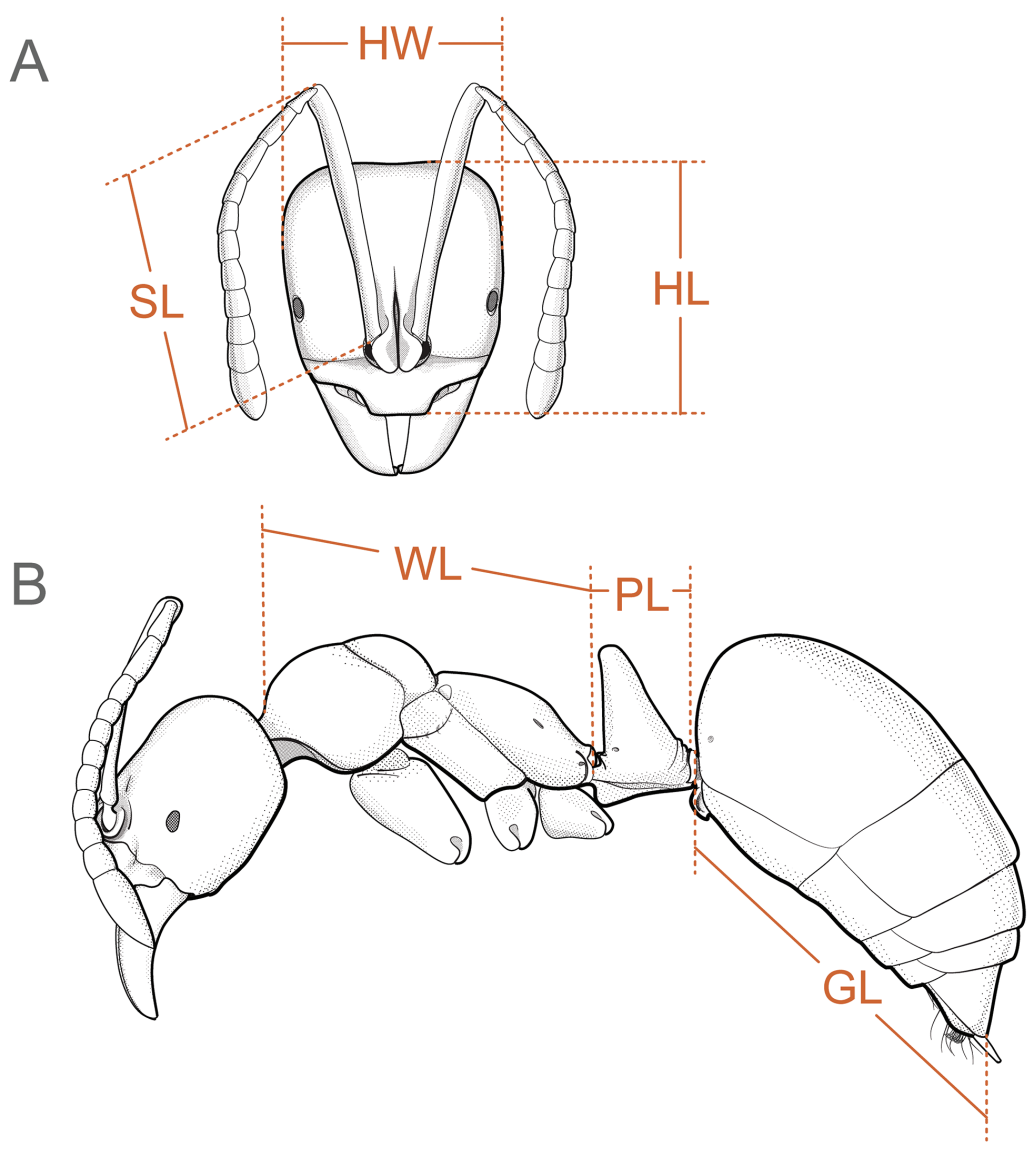
Measurements taken from *Corrieoponenouragues* workers and queen **A** head in full-face view **B** body in profile. Abbreviations: **GL**, gaster length; **HL**, head length; **HW**, head width; **PL**, petiolar length; **SL**, scape length; **WL**, Weber’s length of mesosoma. Illustrations by FA Esteves.

**HL** Head length (Fig. 9A): Maximum longitudinal length of the head, measured from the anteriormost portion of the projecting clypeus to the midpoint of an imaginary line traced across the posterior margin of the head.

**HW** Head width (Fig. 9A): Maximum width of head, excluding eyes.

**SL** Scape length (Fig. 9A): Maximum chord length of the main shaft of the antennal scape, excluding basal bulbus and bulbus neck.

**WL** Weber’s length (Fig. 9B): With the mesosoma in profile, the diagonal length from the posteroventral corner of the propodeum to the farthest point on the anterior face of the pronotum (i.e., anterior inflection point of the pronotum), excluding the neck.

**TL** Total length: Sum of HL + WL + length of segments A2 to A7. A2 to A7 is measured as follows: maximum length of the petiole in profile (PL; Fig. 9B) + A3 to A7, or gaster length (GL; Fig. 9B).

**CI** Cephalic index: HW/HL × 100.

**SI** Scape index: SL/HW × 100.

### ﻿Acronyms

**CASC** California Academy of Sciences, San Francisco, California, U.S.A.

**JTLC** John T. Longino private collection, Salt Lake City, Utah, U.S.A.

**MHNG** Musee d’Histoire Naturelle Genève, Geneva, Switzerland

**MNHN** Muséum Nationale d’Histoire Naturelle, Paris, France

**MZSP** Museu de Zoologia da Universidade de São Paulo, São Paulo, S.P., Brazil

## ﻿Taxonomic treatments

### ﻿Taxonomic changes in Ponerinae

Before describing *Corrieoponenouragues* gen. nov., sp. nov., we must make some adjustments to the taxonomic framework proposed for Ponerinae by Schmidt and Shattuck (2014), which otherwise would render comments on morphological similarities and differences among genera in subsequent sections cumbersome. The following changes are based on assessing female morphology among specimens examined in this study, with images available on AntWeb, and information gathered from relevant taxonomic literature.

### ﻿Transfers between *Neoponera* and *Pachycondyla*

*Neoponera* Emery and *Pachycondyla* Smith are recognized among other Neotropical ponerines by the following combination of characters: The anterior clypeal margin lacks a pair of large teeth-like projections. Torular lobes are closely approximated. Mandibles are triangular to subtriangular, inserted on the anterolateral corner of the head, and armed with numerous teeth. The metapleural gland orifice is closely skirted medially and posteriorly by a well-developed carina. The propodeal spiracle is usually slit-shaped; otherwise, the head presents a bilateral carina between the clypeal margin and the anterior margin of the compound eye, and the pretergite of abdominal segment IV presents a stridulitrum. The mesotibia lacks stout, spine-like setae along its dorsal face, and the metatibia presents two spurs. Pretarsal claws are not pectinate. The petiole sternite lacks a posterior spatulate projection that folds posteriad over the remaining sternite; otherwise, the anterior clypeal margin is convex and angulate (see Suppl. material 3: Table S3; Mackay and Mackay 2010; Schmidt and Shattuck 2014).

**Figure 10.**
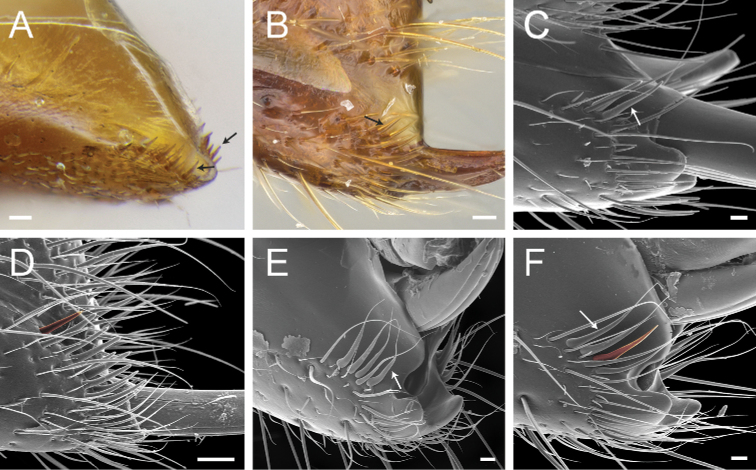
Stout setae on the posterolateral face of the hypopygium in *Neoponera* and *Pachycondyla***A***Neoponerabucki*, worker (UFV-LABECOL-007493); hypopygium disassociated from the pygidium; arrows indicate the spine-like setae **B***N.carinulata*, worker (CASENT0845443); arrow indicates the spine-like setae **C***Pachycondylacrassinoda*, worker (CASENT0370917); arrow indicates the spine-like setae **D***P.harpax*, worker (CASENT0374616); spine-like seta highlighted in orange **E***P.crassinoda*, worker (CASENT0372235); left side; arrow indicates the aristate setae **F***P.crassinoda*, worker (CASENT0372235); right side; arrow indicates the aristate setae; spine-like seta highlighted in orange. Images by JCM Chaul (**A**) and FA Esteves (**B–F**); available at AntWeb.org. Scale bars: 0.04 mm (**A–C, E, F**); 0.06 mm (**D**).

According to our assessment, *Neoponera* and *Pachycondyla* can be set apart from each other by only two characters: the former genus presents distinct arolia between the pretarsal claws and a stridulitrum on the pretergite of abdominal segment IV; the latter does not (see Suppl. material 3: Table S3; Kempf 1961a; Mackay and Mackay 2010; Fernandes et al. 2014; Schmidt and Shattuck 2014). Contrary to Schmidt and Shattuck (2014), the presence or absence of stout, spine-like setae on the posterior portion of the hypopygium is of little importance to distinguish the genera for two reasons. First, the hypopygium bears spine-like setae in some *Neoponera* species [e.g., *N.bucki* (Borgmeier), *N.cavinodis*, *N.crenata*, *N.striadinodis*, and *N.unidentata*; Fig. 10A, B], which are similar to those found in *Pachycondyla* (Fig. 10C, D). Second, in every *Pachycondyla* species examined (*N* = 6), some specimens presented a hypopygium armed with aristate setae instead (Fig. 10E). Moreover, among those aristate setae, a few had blunt-apices and resembled spines (Fig. 10F). We assume that this intraspecific and intraindividual shape variation is caused by wear. For example, contact with prey during stinging could erode the apices of pristine aristate setae on the hypopygium, which would then resemble spines.

Other characters with inconsistent diagnostic value include the presence of a metanotal sulcus and the shape of the petiole. The metanotal sulcus is distinctly impressed in most *Neoponera* species, but is absent to shallowly marked or shows variation in distinctiveness in *N.bucki* (CASENT0915250), *N.crenata* species-group members (sensu Mackay and Mackay 2010; CASENT0923100), and *N.laevigata* (ATPFOR2006, CASENT0902510). In most *Pachycondyla*, the sulcus is obliterated, yet is present in some specimens of *P.harpax* (specimen CASENT0104808), *P.impressa* (ECOFOG-IT14-0276-06), *P.lattkei* (CASENT0217562), and *P.striata* (CASENT0178185, CASENT0249158). The shape of the petiole in profile ranges from scale-like to cuboid in *Neoponera* [as in *N.carbonaria* (Smith), CASENT0915655, and *N.crenata*, CASENT0915261, respectively]. It is cuboid in most *Pachycondyla* species, but also D-shaped in *P.harpax* (CASENT0249149) and scale-like in *P.lenkoi* Kempf (UFV-LABECOL-000002).

**Figure 11.**
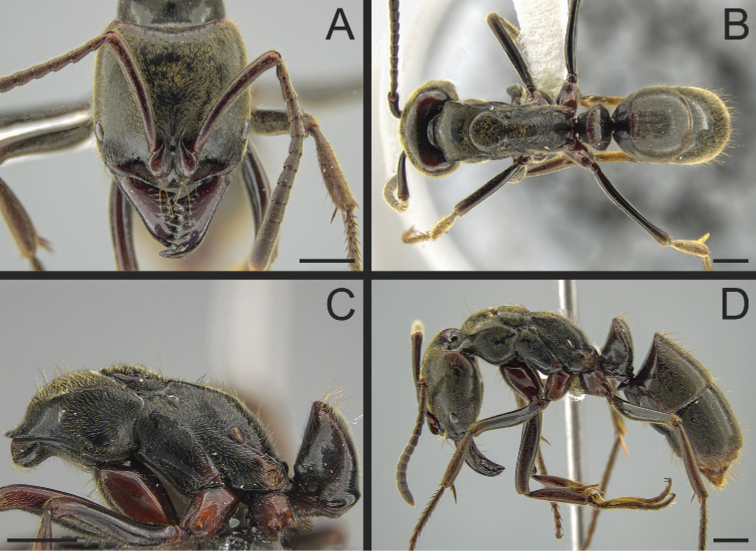
*Pachycondylaprocidua* comb. rev.; worker **A** head in full-face view (CASENT0646046) **B** dorsal view (CASENT0646046) **C** mesosoma in profile (CASENT0830573) **D** body in profile (CASENT0646046). Images by JT Longino (**A, B, D**) and FA Esteves (**C**); available at AntWeb.org. Scale bars: 1 mm.

**Figure 12.**
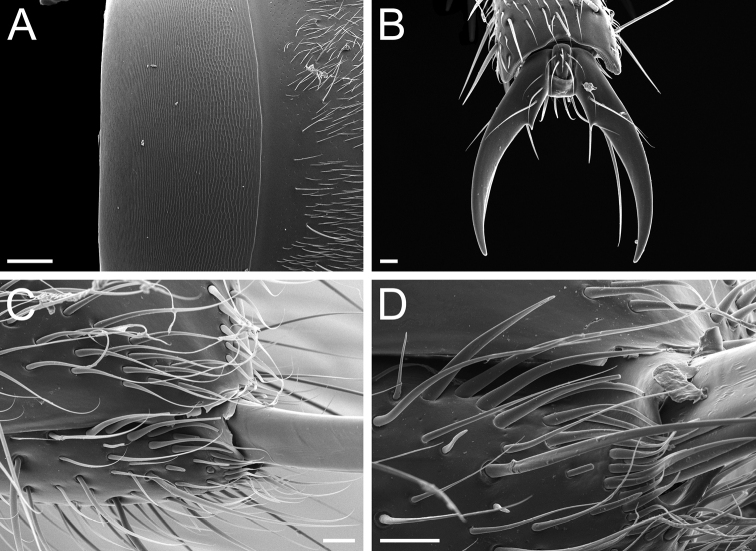
*Pachycondylaprocidua* comb. rev.; worker (CASENT0830573) **A** median area of the abdominal pretergite IV in dorsal view **B** pretarsus and apex of apicalmost tarsomere in posterodorsal view **C** posterolateral face of the hypopygium **D** close-up of the posterolateral face of the hypopygium. Images by FA Esteves; available at AntWeb.org. Scale bars: 0.02 mm (**A, B**); 0.04 mm (**C, D**).

The Neotropical *Pachycondylaprocidua* Emery, 1890 **comb. rev.** (Fig. 11) was previously assigned to *Neoponera* by Schmidt and Shattuck (2014). Yet, the species lacks a stridulitrum and distinct arolia (Fig. 12A, B; Kempf 1964). Additionally, it presents the above characters that discriminate *Neoponera* and *Pachycondyla* from ponerines in the neotropics (Fig. 11; Kempf 1964; Mackay and Mackay 2010). The metanotal sulcus is distinct (Fig. 11B), while the petiole is scale-like (Fig. 11C, D). The two specimens we examined present aristate hypopygial setae. The setae seem fragile, and in one specimen, most were abraded by the cleaning procedure that preceded SEM imaging (Fig. 12C, D).

**Figure 13.**
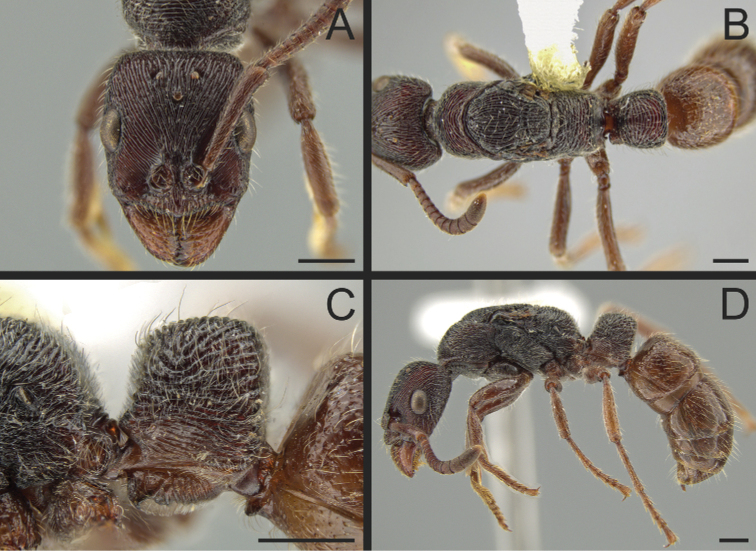
*Neoponeracuriosa* comb. nov.; holotype, dealated queen (LACMENT226103) **A** head in full-face view **B** dorsal view **C** petiole in profile **D** body in profile. Images by JE Lattke; available at AntWeb.org. Scale bars: 0.5 mm.

*Neoponeracuriosa* (Mackay & Mackay, 2010) **comb. nov.** also conforms with the characters shared by *Neoponera* and *Pachycondyla* (Fig. 13; Mackay and Mackay 2010). In addition, the species possesses a stridulitrum on the pretergite of abdominal segment IV (Mackay and Mackay 2010). According to the authors of the taxon, the arolia are underdeveloped. However, note that the adjective was likely used to compare the taxon with other species formerly assigned to *Pachycondyla* in which the arolium is distinct. For example, Mackay and Mackay (2010) also described the trait as underdeveloped in *N.striadinodis*; yet we examined one specimen determined by W. P. Mackay that has distinct arolia.

### ﻿Transfer from *Euponera* to *Leptogenys* and new synonymy

The genus *Leptogenys* Roger occurs in the tropics and subtropics around the world. Most of its species present pectinate or multidentate pretarsal claws, absent elsewhere in Ponerinae (Bolton 1975; Schmidt and Shattuck 2014; Fisher and Bolton 2016). The clypeal medial area usually projects anteriad into a well-developed, angular prominence, generally skirted anteriorly by a translucid lamella; the median area usually bears a longitudinal carina. Torular lobes are small and only conceal the medial portion of the antennal sockets in dorsal view. Mandibles insert on the anterolateral corners of the head. Mandible shapes range from subtriangular to oblique, to falcate, to linear, to bizarre forms in-between. The propodeal spiracle usually presents a round to oval orifice. The metatibia bears two spurs; the metabasitarsus does not bear stout, spine-like setae on its dorsal face. The helcium is infra-axial (i.e., helcium positioned ventrad to the midheight of the anterior face of abdominal segment III).

**Figure 14.**
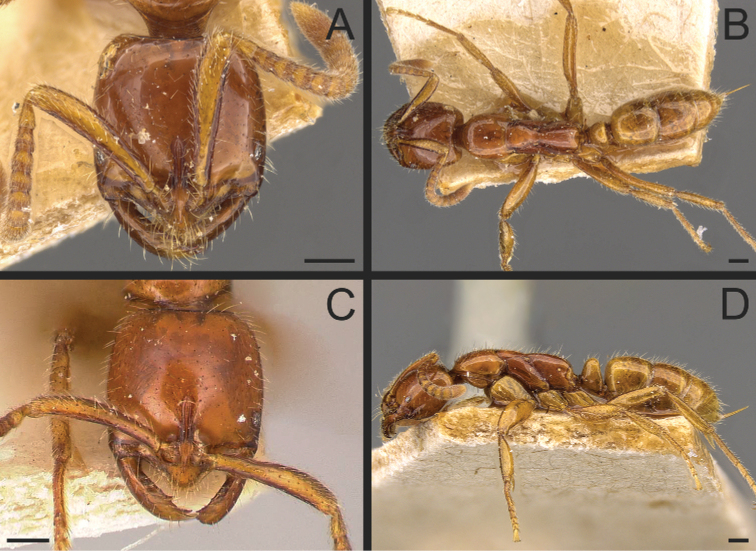
*Leptogenysbutteli* comb. nov.; syntypes, worker caste **A** head in full-face view (CASENT0907293) **B** dorsal view (CASENT0907293) **C** head in full-face view (FOCOL1013) **D** view in profile (CASENT0907293). Images by Z Lieberman (**A, B, D**) and C Klingenberg (**C**); available at AntWeb.org. Scale bars: 0.2 mm.

The species originally described as *Pseudoponerabutteli* Forel, 1913, based on specimens collected in Java, Indonesia, was recently assigned to the genus *Euponera* Forel by Schmidt and Shattuck (2014). However, the above characters setting *Leptogenys* apart from other ponerines are visible in images of its syntypes (Fig. 14; specimens CASENT0907293, FOCOL1012, FOCOL1013) or were mentioned in its description (Forel 1913), except for the shape of the pretarsal claws. On our behalf, entomologist and curator Dr B. Landry examined one of the syntypes deposited at the NHMG and confirmed the presence of three preapical teeth on the hindleg claws. Therefore, *E.butteli* becomes *Leptogenysbutteli* (Forel, 1913) **comb. nov.**

*Leptogenysbutteli* belongs to the *L.processionalis* species group (sensu Schmidt 2013), which contains ~15 taxa occurring in the Indomalayan and Australasian regions, viz.: *L.birmana* Forel, specimen CASENT0907581; *L.breviceps* Viehmeyer, CASENT0902610; *L.crassicornis* Emery, CASENT0903953; *L.chelifera* (Santschi), CASENT0915218; *L.dentilobis* Forel, CASENT0907365; *L.fallax* (Mayr), CASENT0915877; *L.fortior* Forel, CASENT0907393; *L.iridescens* (Smith), CASENT0901354; *L.lucidula* Emery, CASENT0235337; *L.mutabilis* (Smith), CASENT0901355; *L.myops* (Emery), CASENT0903952; *L.processionalis* (Jerdon), CASENT0270567; *L.processionalisdistinguenda* (Emery), CASENT0903955; *L.strena* Zhou; and *L.tricosa* Taylor. Members of this species group are characterized by a quadrate or subquadrate head; oblique mandibles armed with more than three teeth, without distinct basal and masticatory margins (somewhat subtriangular in *L.processionalisdistinguenda*); and scale-like petiole (somewhat nodiform in *L.crassicornis*, *L.tricosa*, and apparently also in *L.strena*).

**Figure 15.**
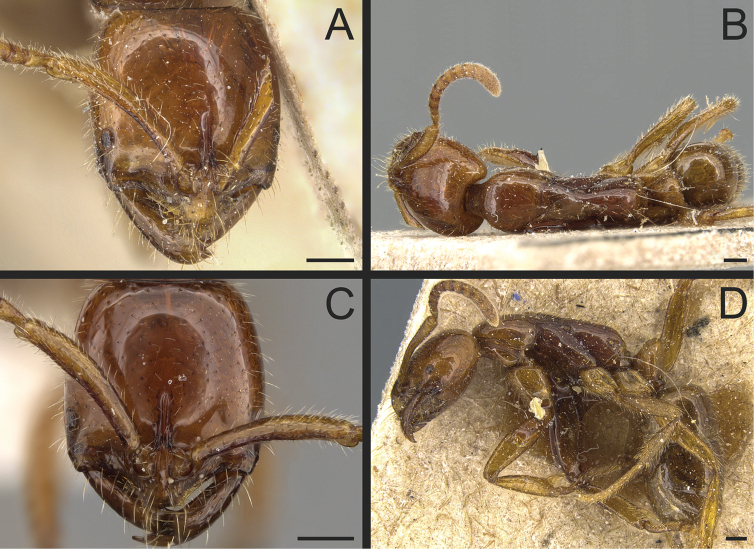
*Leptogenysmyops*; worker caste **A** head in full-face view; holotype (CASENT0903952) **B** dorsal view; holotype (CASENT0903952) **C** head in full-face view (CASENT0281925) **D** view in profile; holotype (CASENT0903952). Images by W Ericson (**A, B, D**) and S Hartman (**C**); available at AntWeb.org. Scale bars: 0.2 mm.

When we compared type specimen images and evaluated morphological variation among *L.processionalis* group members, we found several characters distinguishing between most species in the group. However, we could not find any significant differences between *L.butteli* and *L.myops* (Fig. 15). Their type specimens (all from Java, Indonesia) present the same head proportions and sculpture; shape of the torular lobes; shape of the clypeal anterior projection; width of the longitudinal protrusion on the clypeal median area; distance from the apex of the antennal scape to the posterior margin of the head; size, shape, and location of compound eyes on the head; sculpture and shape of the mandibles; dorsal outline of the mesosoma in profile; indistinctiveness of the metanotal sulcus; shape of the mesonotum in dorsal view; height of the petiole in comparison with the propodeum; shape of the petiolar tergite; body-color; and approximate length, amount, and inclination of standing setae. Consequently, we synonymize *Leptogenysbutteli* (Forel, 1913), **syn. nov.**, with *Leptogenysmyops* (Emery, 1887).

### ﻿Transfer from *Mesoponera* to *Bothroponera*

In the Afrotropics, the genera *Bothroponera* Mayr and *Mesoponera* Emery can be discriminated from other ponerines by a combination of characters in the worker caste (Fisher and Bolton 2016). The anterior portion of the head lacks a dorsolateral carina between the clypeal margin and the anterior margin of the eye. Torular lobes are closely approximated medially. The anterior margin of the eyes is located at or anteriad to the midlength of the head. Mandibles are triangular and inserted on the anterolateral corners of head; masticatory margins bear four or more teeth; basolateral or dorsal faces lack pits of any shape (an oblique dorsolateral sulcus may be present). The dorsoposterior area of the propodeum is devoid of spine-like or tooth-like projections. The mesotibia lacks stout spine-like setae on its dorsal face, and the metatibia bears two distinct spurs on its ventroapical face. The helcium is positioned ventrad the midlength of the anterior face of abdominal segment IV. Note that contrary to Schmidt and Shattuck (2014), only the basal portion of the masticatory margin of the mandibles is edentate in *M.subiridescens*; the apical portion is armed with four to seven, but sometimes more, teeth (the count includes denticles).

*Bothroponera* species have a propodeum with a broad dorsal face, a slit-shaped propodeal spiracle, and a nodiform petiole (Schmidt and Shattuck 2014). The metanotal sulcus is usually obliterated; however, intraspecific variation exists, and the sulcus is weakly impressed in some specimens [as *B.ilgii* (Forel), specimen CASENT0235600; and *B.kruegeri* (Forel), CASENT0235604]. Regardless, if present, the sulcus does not interrupt the dorsal outline of the mesosoma in profile. On abdominal segment IV, a strongly impressed constriction separates presclerites from postsclerites, as in *B.berthoudi* (Forel), specimen CASENT0902470; or moderately so, as in *B.silvestrii*CASENT0235599. Schmidt and Shattuck (2014) assigned *Bothroponera* species in two groups. The *B.sensustricto* group members share a strongly sculptured body, hypertrophied torular lobes, and a metapleural gland orifice closely skirted medially and posteriorly by a well-developed carina; these characters are absent in the *B.sulcata* group. Notwithstanding the differential diagnosis given by the authors above, several species of the *sensu stricto* group [e.g., *B.berthoudi*, CASENT0902470; *B.granosa* (Roger), CASENT0250375; and *B.laevissima* (Arnold), CASENT0902471] have the propodeal dorsum as narrow as those of the *sulcata* group members.

In *Mesoponera*, the propodeum is tectiform (i.e., roof-shaped), with its lateral surfaces diverging while sloping ventrad from the noticeably narrow dorsum. In most species, the propodeal dorsal face presents slightly or moderately bulging lateral margins, with the medial area slightly concave posteriorly; the lateral margins may be somewhat parallel to one another (as in *M.caffraria*, CASENT0915251) or diverge continuously posteriad (as in *M.ambigua*, CASENT0249194). In a few species, the propodeal dorsum is narrower and transversely convex (as in *M.subiridescens*, CASENT0003151). The propodeal spiracle is usually round to oval [except for *M.caffraria* and subspecies, *M.ingesta* (Wheeler), and *M.subiridescens*; see CASENT0906219]. The petiole is shaped like an upward-pointing wedge in profile, with the anterior and posterior faces of the tergite tapering to a thin dorsal margin. The metanotal sulcus is clearly distinct and usually deeply impressed, and it indents the mesosoma outline in profile. The constriction between the presclerites and postsclerites of abdominal segment IV may be obliterated to moderately impressed. The torular lobes are not hypertrophied. Body is mostly smooth and shiny or densely and uniformly sculptured by fine punctures. These characters are as described here in every *Mesoponera* species except one.

**Figure 16.**
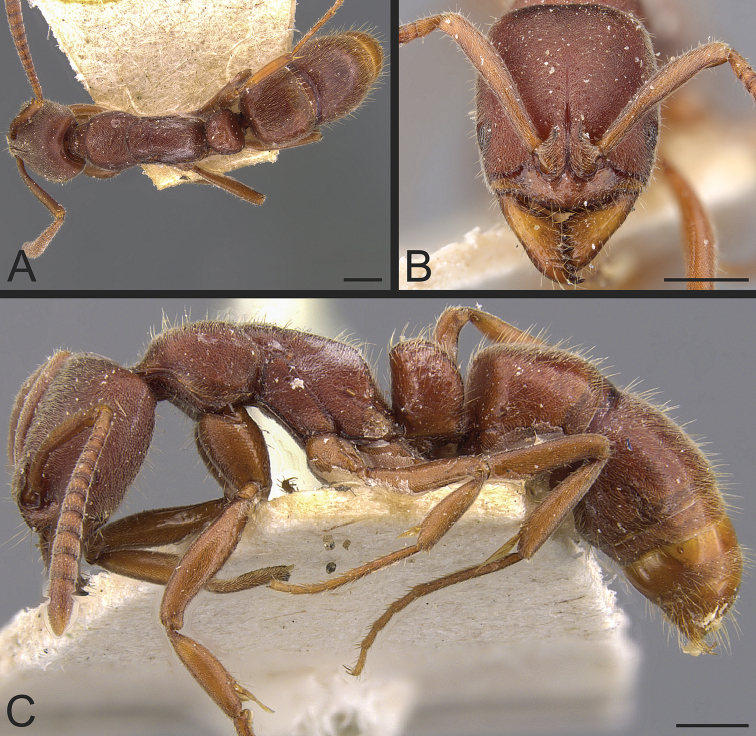
*Bothroponeraescherichi* comb. nov.; holotype, worker (CASENT0907252) **A** dorsal view **B** head in full-face view **C** view in profile. Images by W Ericson; available at AntWeb.org. Scale bars: 0.5 mm.

The species originally described as Pachycondyla (Bothroponera) escherichi Forel, 1910, based on one worker collected in Eritrea (Fig. 16), was recently combined in *Mesoponera* by Schmidt and Shattuck (2014). However, the species is clearly misassigned. *Mesoponeraescherichi* presents every character that sets *Bothroponera* and *Mesoponera* apart from the remaining Ponerinae genera in the Afrotropics, including the absence of a basolateral or dorsal pit on the mandibles (Forel 1910). The shape of the propodeal dorsal face corresponds to that of the *B.sulcata* group. The propodeal spiracle is slit-shaped. The petiolar tergite is unaligned in the image we evaluated; even so, it shows a somewhat nodiform petiole (clearly not cuneiform) that is reasonably accommodated by the variation seen in the *Bothroponera*. Moreover, Forel (1910) stated that the anterior and posterior faces of the petiole are vertical surfaces. The metanotal sulcus is present but weakly impressed, and does not interrupt the dorsal outline of the mesosoma in profile. The presclerites and postsclerites of abdominal segment IV are separated by a moderately impressed constriction. The torular lobes are not hypertrophied. Sculpture is not strongly impressed: most of the head and the dorsal face of the mesosoma is densely foveolate (i.e., covered by small pits that are wider than punctures); the lateral face of the mesosoma is mostly costulate; the petiole is somewhat shiny; the gaster is slightly punctate, and is shinier than the head and mesosoma. Therefore, *M.escherichi* becomes *Bothroponeraescherichi* (Forel, 1910), **comb. nov.**, a member of the *B.sulcata* species group.

**Figure 17.**
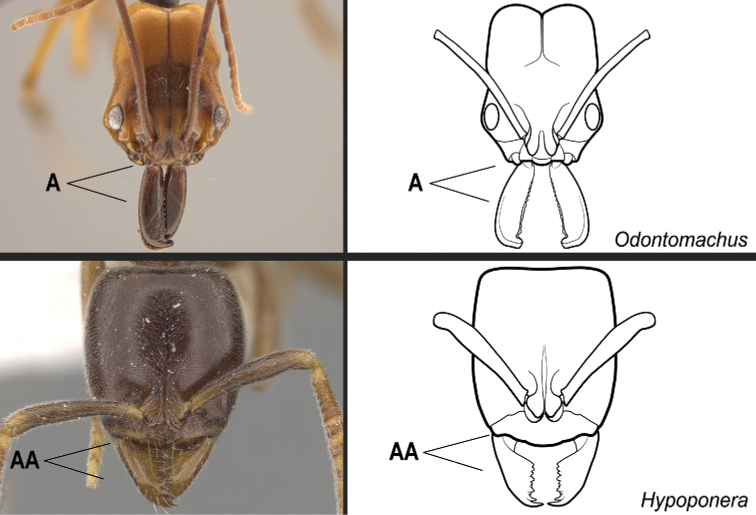
Identification key, couplet 1 **A***Odontomachuserythrocephalus*, worker (CASENT0649085) **AA***Hypoponeraaliena*, worker (CASENT0281913). Images by JT Longino and S Hartman, respectively; available at AntWeb.org. Illustrations by Jessica Huppi.

### ﻿Identification key for the Neotropical genera of Ponerinae based on workers

We did not consider *Pachycondylavieirai* Mackay & Mackay in this key, as it was not examined and the taxon description was uninformative for our purposes. The species was considered incertae sedis in *Pachycondyla* (Schmidt and Shattuck 2014).

**Table:** 

**1**	Mandibles long and linear in full-face view, inserted at the middle of the anterior margin of the head, their bases closely approximate (Fig. 17A)	**2**
–	Mandibles with variable shape, but always inserted at the anterolateral corners of the head, their bases conspicuously separated (Fig. 17AA)	**3**
**2**	Nuchal carina (i.e., carina that separates dorsal from posterior surfaces of the head) and paired dark posterior apophyseal lines converge in a V-shape at the midline of the posterior margin of the head (Fig. 18A, B), and join a sharp mediodorsal sulcus that runs longitudinally on the posterior half of the head. Dorsalmost tooth of apical mandibular series usually truncated. Apex of the petiolar node usually conical or pointed	***Odontomachus***
–	Nuchal carina forms an uninterrupted curve across the posterodorsal extremity of the head (Fig. 18AA); paired dark apophyseal lines absent (Fig. 18BB); median sulcus absent or ill-defined and shallow on the posterior half of the head. Dorsalmost tooth of apical mandibular series usually acute. Petiolar node with varying shape: subtriangular to scale-like, unarmed to bidentate	***Anochetus***
**3**	In full-face view, anterior part of the torular lobes widely separated and usually not confluent; separated by a rounded, truncated, or broadly triangular section of the clypeus (Fig. 19A). The lateral margins of the lobes variously shaped, but only rarely with a pinched-in appearance posteriorly (Fig. 19B)	**4**
–	In full-face view, anterior section of the torular lobes confluent or closely approximated; separated by a narrow triangular portion of the clypeus or by a very narrow cuticular strip, which extends posteriad between them (Fig. 19AA). The lateral margins of the lobes always with a pinched-in appearance posteriorly (Fig. 19BB)	**5**
**4**	Mandible triangular, with distinct basal and masticatory margins; masticatory margin edentate or with numerous short teeth (Fig. 20A). Metatibia with 2 distinctly pectinate spurs; the posterior spur usually much larger than the anterior (Fig. 20B). Helcium in profile located approximately at midheight of the anterior face of the first gastral segment (abdominal segment III), so that the first gastral segment does not have a long vertical anterior face in profile. Petiole subrectangular to subcylindrical; the posterior face usually with carinate lateral margins. Dorsal surfaces of the head and the mesosoma without erect/suberect setae. Fine, dense shagreened sculpture, with associated larger punctures	***Platythyrea***
–	Mandible pitchfork-like; with indistinct basal and masticatory margins; armed with 3 noticeably long, curved teeth; the apical tooth so long and curved that it reaches or surpasses the anterolateral corner of the head opposite from its insertion when the mandible is closed (Fig. 20AA). Metatibia with only one spur, pectinate (Fig. 20BB). Helcium in profile located ventrad to the midheight of the anterior face of the first gastral segment (abdominal segment III), so that the first gastral segment has a long vertical anterior face in profile. Shape of the petiole ranging from a thick, broad scale with sharp lateral margins to a somewhat cuboid node; never subrectangular to subcylindrical with carinate posterolateral margins. Dorsal surfaces of the head and the mesosoma usually with erect/suberect setae, at least partially. Sculpture varying from smooth and shiny to finely shagreened to finely punctate and rugulose	***Thaumatomyrmex***
**5**	Ventral apex of the metatibia with only one spur, which is large and pectinate (Fig. 21A)	**6**
–	Ventral apex of the metatibia with two spurs; the posterior spur always larger and pectinate (Fig. 21AA)	**9**
**6**	Dorsal face of the metabasitarsus with stout, spine-like setae amid regular, filiform setae (Fig. 22A); similar spine-like setae also present on mesobasitarsus and mesotibia	***Centromyrmex***
–	Dorsal face of the metabasitarsus vested with filiform setae; stout, spine-like setae absent (Fig. 22AA). Stout, spine-like setae may occur on either mesobasitarsus or mesotibia, but if so, they are absent from the metabasitarsus	**7**
**7**	Medial portion of the clypeus projected anteriad, overhanging the anterior clypeal margin in full-face view (Fig. 23A); projection frequently mucronate: anteromedian point with an abrupt, thin, conspicuous prominence (Fig. 23A, mid-image). Mandible subtriangular to falcate (Fig. 23B). Arolium usually well-developed (Fig. 23C)	***Simopelta***
–	Medial portion of the clypeus does not overhang the anterior clypeal margin in full-face view (Fig. 23AA); anterior clypeal margin slightly convex. Mandible triangular (Fig. 23BB). Arolia indistinct (Fig. 23CC)	**8**
**8**	Subpetiolar process in ventrolateral (oblique) view with a pair of angulate projections located posteriorly (Fig. 24A). In profile, subpetiolar process with an anterior translucent fenestra (Fig. 24B), and with a sharp posteroventral angle (Fig. 24C). Maxillary palps with 2 segments	***Ponera***
–	Subpetiolar process in ventrolateral (oblique) view without a pair of angulate projections located posteriorly (Fig. 24AA). In profile, subpetiolar process usually without an anterior fenestra (Fig. 24BB); with posteroventral portion rounded to acutely angulate (Fig. 24CC). Maxillary palps with 0–1 segments	***Hypoponera***
**9**	Hindlegs usually with pectinate pretarsal claws (Fig. 25A), rarely with only 1–2 small preapical teeth; if pretarsal claws not pectinate, then mandible with only 1–2 teeth; if mandibles with > 3 teeth, then pretarsal claws pectinate. Torular lobes distinctly fail to cover the entire antennal sockets in full-face view (Fig. 25B)	***Leptogenys***
–	Pretarsal claws of hindlegs never pectinate; the claws are simple or with a basal or preapical tooth (Fig. 25AA). Mandible edentate or with variable numbers of teeth; if basal or preapical teeth are present on pretarsal claws, then mandible with 4 or more teeth. The torular lobes may or may not conceal the antennal sockets in full-face view (Fig. 25BB)	**10**
**10**	Mandibles falcate, elongated, and slender, with long, conspicuous teeth; apical tooth much longer than other teeth (Fig. 26A)	***Belonopelta***
–	Mandibles triangular or subtriangular, without long, conspicuous teeth (Fig. 26AA)	**11**
**11**	Dorsal face of mesotibiae covered with abundant, stout, spine-like setae (Fig. 27A). Prora variable, usually reduced and hardly visible in profile, but never projected ventro-anteriorly as a long, acute prominence (Fig. 28A)	***Cryptopone***
–	Dorsal face of mesotibiae usually without abundant, stout, spine-like setae (Fig. 27AA). If spine-like setae present along dorsal face of mesotibia (Fig. 27AAA), then prora in profile projected ventro-anteriorly as a long, acute prominence (Fig. 28AA); otherwise prora with variable shape	**12**
**12**	Massive ants (head width greater than 4.0 mm). Anterior clypeal margin with a pair of large projecting teeth (Fig. 29A)	***Dinoponera***
–	Smaller ants (head width less than 4.0 mm). Anterior clypeal margin without a pair of large projecting teeth (Fig. 29AA)	**13**
**13**	In ventral view, petiolar sternite with a posterior spatulate projection folded posteriad over the remaining sternite, so that in profile, the posterior portion of the subpetiolar process presents a long, acute projection strongly directed posteriad (Fig. 30A). Anterior margin of clypeus truncated to emarginated, never entirely convex	***Rasopone***
–	Petiolar sternite usually without a posterior spatulate projection folded posteriad over the remaining sternite (Fig. 30AA). If a posterior spatulate projection is present and the posterior portion of the subpetiolar process is somewhat directed posteriad in profile, then the anterior clypeal margin is convex and angulate; otherwise, the anterior margin of clypeus is variable in shape	**14**
**14**	Mandible edentate (Fig. 31A). Clypeus in full-face view with a truncate anteromedial projection that overhangs the basal portion of the closed mandibles (Fig. 31B). Ventral face of the hypopygium (abdominal segment VII sternite) longitudinally concave, with posterior region bearing stout, hook-shaped setae (Fig. 31C, D); in profile, hook-shaped setae visible. Gaster in profile and in dorsal view without a girdling constriction (presclerites of abdominal segment IV, the second gastral segment, forming an even surface with postsclerites; Fig. 31E)	***Corrieopone* gen. nov.**
–	Mandible dentate (with > 4 teeth/denticles; Fig. 31AA). Clypeus variable in shape, but never with truncate anteromedial projection that overhangs the mandibles (Fig. 31BB). Ventral face of the hypopygium (abdominal segment VII sternite) without longitudinal concavity, and never bearing stout, hook-shaped setae (Fig. 31CC, DD). Gaster in profile and in dorsal view with or without a distinct impression between the presclerites and postsclerites of the second gastral segment (abdominal segment IV) that appears as a girdling constriction (Fig. 31EE)	**15**
**15**	Stridulitrum present on abdominal pretergite IV (Fig. 32A)	**16**
–	Stridulitrum absent from abdominal pretergite IV (Fig. 32AA)	**17**
**16**	Propodeal spiracle round or ovoid (Fig. 33A), never slit-shaped, and preocular carina absent (Fig. 33B)	***Mayaponera*** (part)
–	Propodeal spiracle usually slit-shaped (Fig. 33AA), but if round, the preocular carina is present (Fig. 33BB)	***Neoponera***
**17**	Metapleural gland orifice shaped as a curved slit aperture directed posterodorsally (Fig. 34A). In profile, prora projected ventro-anteriorly as a long, acute prominence (Fig. 34B); in anterior view, prora similar to a soup spoon (or a spatula), transverse on abdominal sternite III. Mandible with 6 or 7 teeth/denticles	***Pseudoponera***
–	Metapleural gland orifice variable, but never shaped as a curved slit aperture (Fig. 34AA). Prora variable, but never projected ventro-anteriorly as a long, acute prominence (Fig. 34BB). Mandibles usually with 9 or more teeth/denticles	**18**
**18**	Propodeal spiracles slit-shaped (Fig. 35A). In full-face view, lateral surfaces of torular lobes with large, smooth, shiny area, which is mostly or entirely glabrous (Fig. 35B). In profile, petiole usually cuboid (Fig. 36A). Hypopygium in profile with spine-like or aristate setae on posteriormost portion (Fig. 36B)	***Pachycondyla***
–	Propodeal spiracles round (Fig. 35AA); never slit-shaped. In full-face view, lateral surfaces of torular lobes covered uniformly by same sculpture and setae (Fig. 35BB). In profile, petiole shaped as an upward-pointing wedge (Fig. 36AA). Hypopygium in profile usually without spine-like or aristate setae on posteriormost portion (Fig. 36BB)	***Mayaponera*** (part)

**Figure 18.**
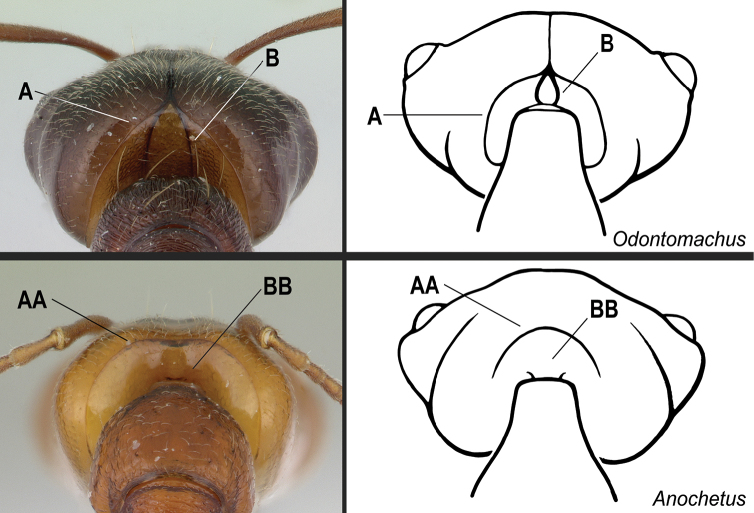
Identification key, couplet 2 **A, B***Odontomachusmeinerti*, worker (CASENT0178690) **AA BB***Anochetusdiegensis*, worker (CASENT0178673). Images by A Nobile; available at AntWeb.org. Illustrations by Jessica Huppi.

**Figure 19.**
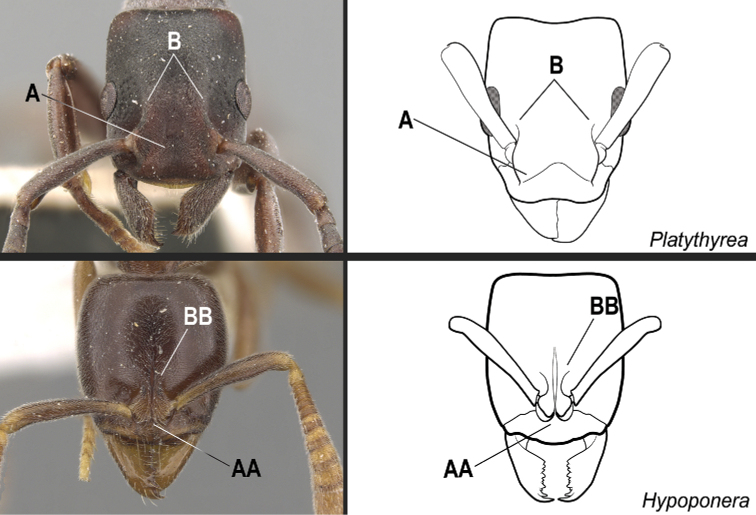
Identification key, couplet 3 **A, B***Platythyreaangusta*, worker (CASENT0907126) **AA, BB***Hypoponeraaliena*, worker (CASENT0281913). Images by W Ericson and S Hartman, respectively; available at AntWeb.org. Illustrations modified from artwork by Jessica Huppi (**A, B**), and by Jessica Huppi (**AA, BB**).

**Figure 20.**
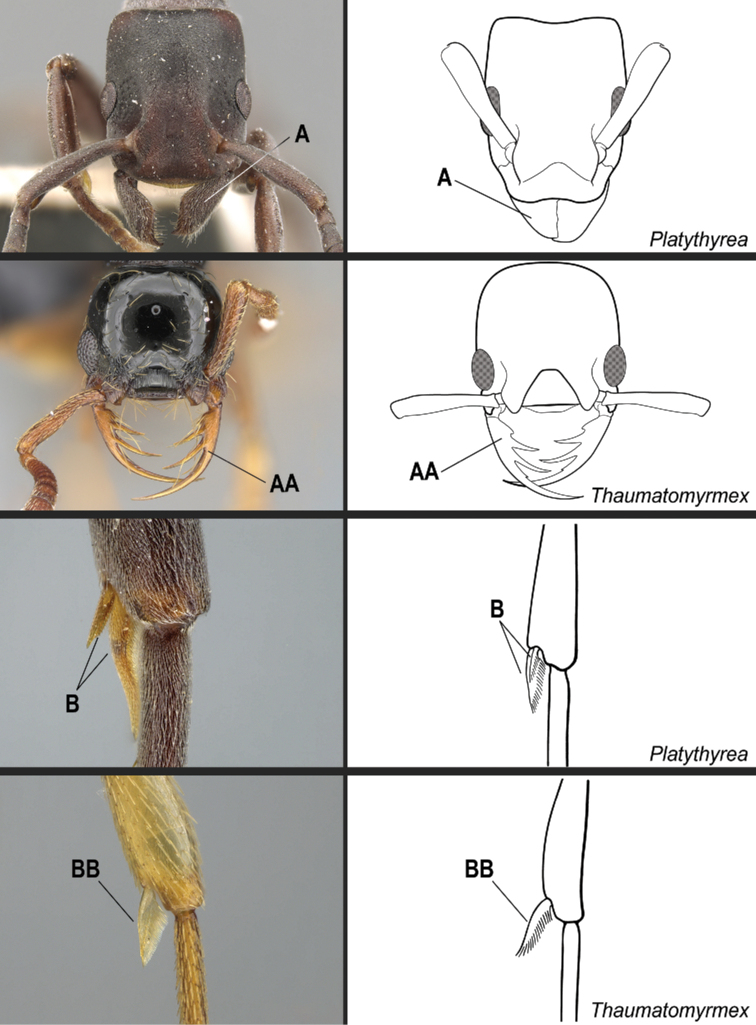
Identification key, couplet 4 **A***Platythyreaangusta*, worker (CASENT0907126) **AA***Thaumatomyrmexzeteki*, worker (CASENT0318451) **B***P.sinuata*, worker (CASENT0217573) **BB***T.zeteki*, worker (CASENT0318452). Images by W Ericson, M Esposito, W Ericson, and FA Esteves, respectively; available at AntWeb.org. Illustrations modified from artwork by Jessica Huppi (**A**), by FA Esteves (**B**), and Jessica Huppi (**B, BB**).

**Figure 21.**
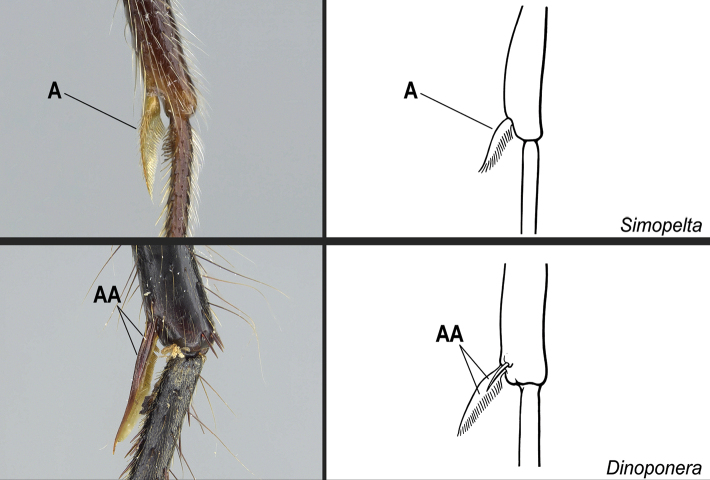
Identification key, couplet 5 **A***Simopeltapaeminosa*, worker (CASENT0217574) **AA***Dinoponeraquadriceps*, worker (CASENT0217519). Images by FA Esteves, available at AntWeb.org. Illustrations by Jessica Huppi.

**Figure 22.**
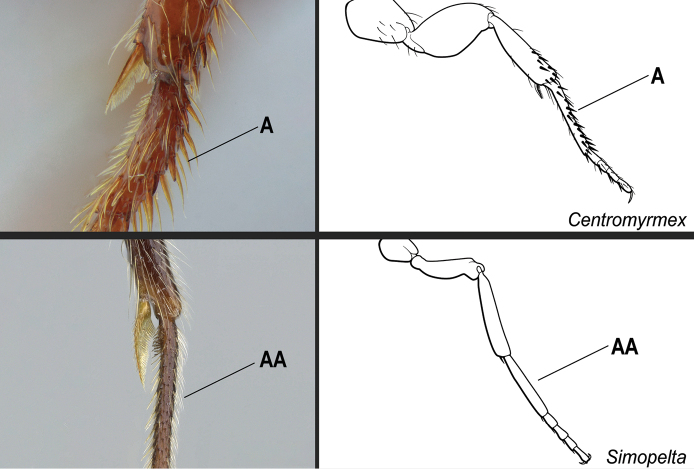
Identification key, couplet 6 **A***Centromyrmexalfaroi*, worker (ANTWEB1032026) **AA***Simopeltapaeminosa*, worker (CASENT0217574). Images by JCM Chaul and FA Esteves, respectively; available at AntWeb.org. Illustrations modified from artwork by Jessica Huppi (**A**), and by Jessica Huppi (**AA**).

**Figure 23.**
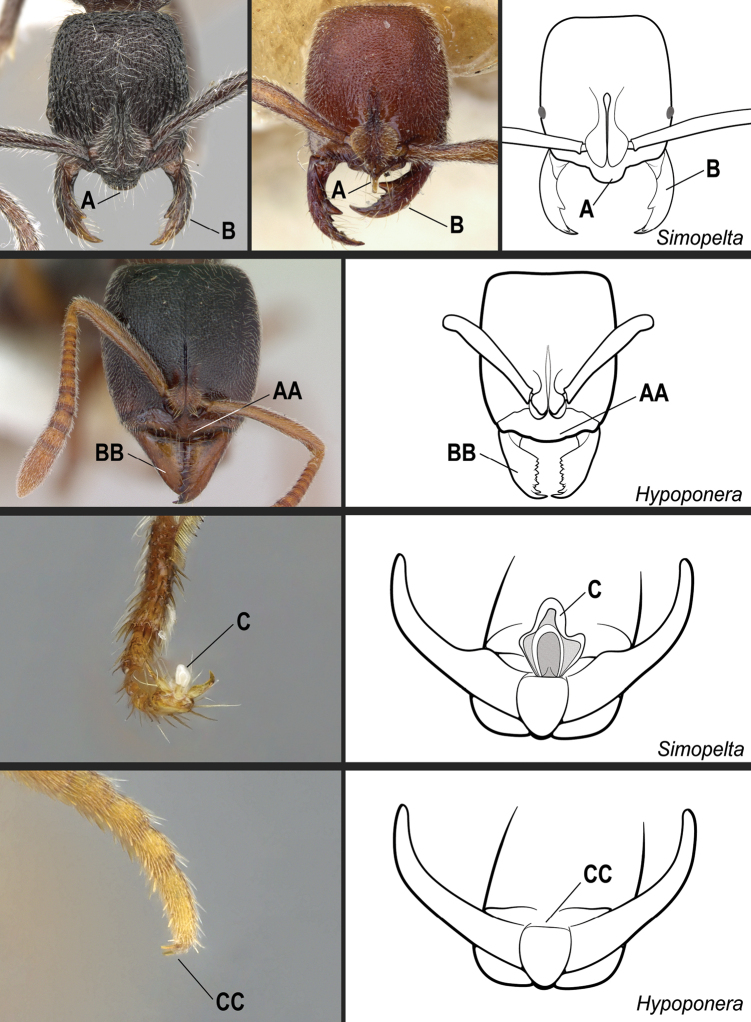
Identification key, couplet 7 **A, B** left image: *Simopeltapaeminosa*, paratype, worker (CASENT0902468); middle image: *S.pergandei*, holotype, worker (CASENT0907294) **AA, BB***Hypoponera* alw06, worker (CASENT0173727) **C***S.paeminosa* (CASENT0902468) **CC***Hypoponera* vc01, worker (CASENT0766197). Images by W Ericson, Z Lieberman, A Nobile, and FA Esteves, respectively; available at AntWeb.org. Illustrations by FA Esteves (**A, B**), Jessica Huppi (**AA, BB**), and modified from artwork by Jessica Huppi (**C, CC**).

**Figure 24.**
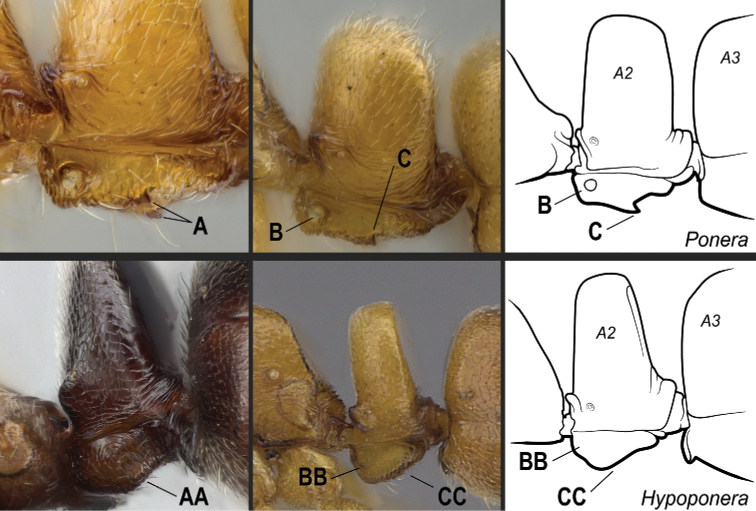
Identification key, couplet 8 **A, B, C***Poneraexotica*, worker (CASENT0100692) **AA***Hypoponera* vc01, worker (CASENT0766197) **BB, CC***H.parva* (CASENT0260431). Images by FA Esteves (**A, B, C, AA**) and S Hartman (**BB, CC**); available at AntWeb.org. Illustrations by Jessica Huppi. Abbreviations: **A2**, abdominal segment II; **A3**, abdominal segment III.

**Figure 25.**
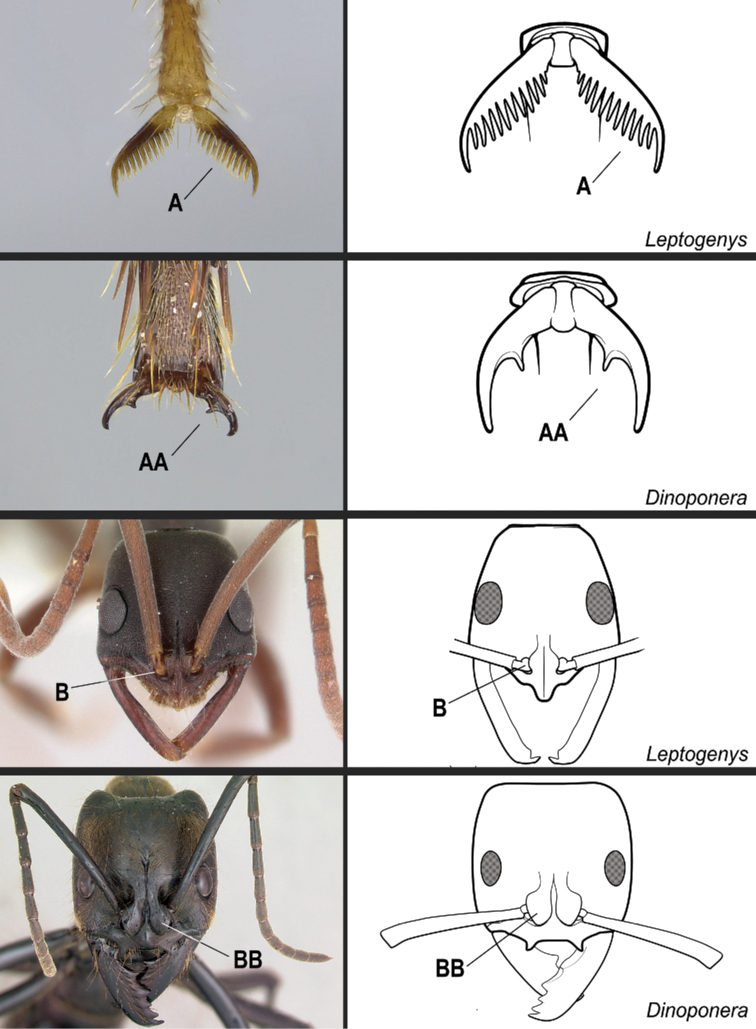
Identification key, couplet 9 **A***Leptogenys* pe02, worker (CASENT0372210) **AA***Dinoponeralongipes*, worker (CASENT0217518) **B***L.wheeleri*, worker (CASENT0178811) **BB***D.longipes*, worker (CASENT0004663). Images by FA Esteves (top two rows) and A Nobile (bottom two rows); available at AntWeb.org. Illustrations modified from artwork by Jessica Huppi (**A, B**), by Jessica Huppi (**AA**), and FA Esteves (**BB**).

**Figure 26.**
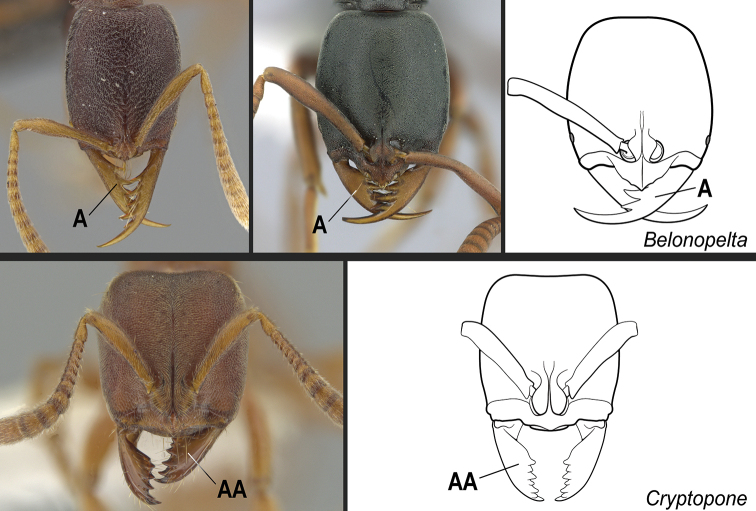
Identification key, couplet 10 **A** left image: *Belonopeltaattenuata*, worker (ICN100255); middle image: *B.deletrix*, worker (CASENT0260514) **AA**Cryptoponecf.guatemalensis (CASENT0646802). Images by JT Longino (top and bottom left) and S Hartman (top middle); available at AntWeb.org. Illustrations by FA Esteves (**A**), and modified from artwork by Jessica Huppi (**AA**).

**Figure 27.**
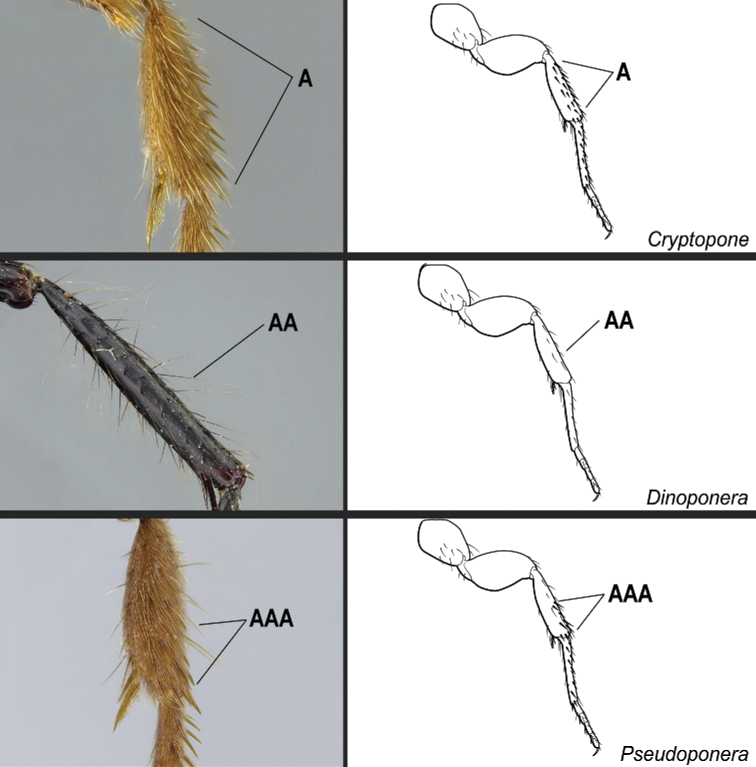
Identification key, couplet 11 **A***Cryptoponegilva*, worker (CASENT0006054) **AA***Dinoponeraquadriceps*, worker (CASENT0217519) **AAA***Pseudoponeracognata*, worker (CASENT0008155). Images by FA Esteves; available at AntWeb.org. Illustrations modified from artwork by Jessica Huppi.

**Figure 28.**
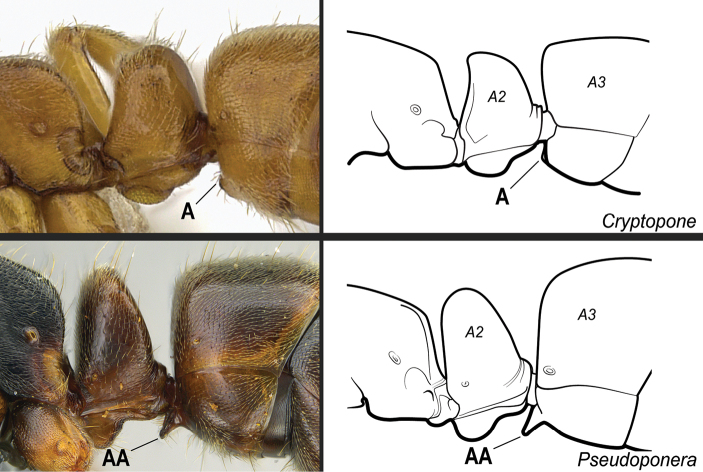
Identification key, couplet 11 **A***Cryptoponegilva*, worker (CASENT0260411) **AA***Pseudoponeragilberti*, worker (CASENT0828638). Images by S Hartman and FA Esteves, respectively; available at AntWeb.org. Illustrations modified from artwork by Jessica Huppi. Abbreviations: **A2**, abdominal segment II; **A3**, abdominal segment III.

**Figure 29.**
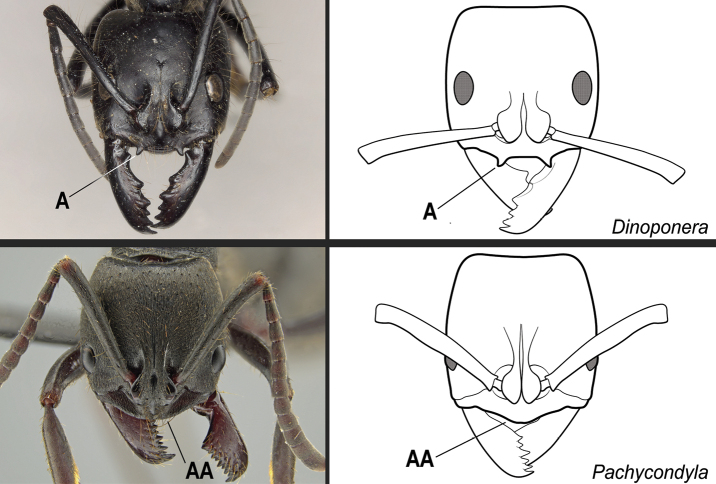
Identification key, couplet 12 **A***Dinoponeraquadriceps*, worker (CASENT0217519) **AA***Pachycondylastriata*, worker (UFV-LABECOL-000291). Images by S Hartman and JT Longino, respectively; available at AntWeb.org. Illustrations by FA Esteves(**A**), and modified from artwork by Jessica Huppi (**AA**).

**Figure 30.**
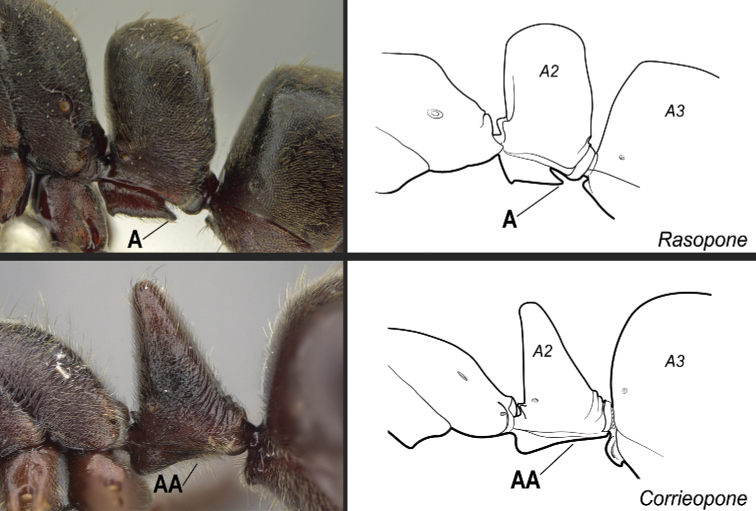
Identification key, couplet 13 **A***Rasoponepanamensis*, worker (CASENT0644252) **AA***Corrieoponenouragues*, holotype, worker (CASENT0830464). Images by JT Longino and W Lee, respectively; available at AntWeb.org. Illustrations modified from artwork by Jessica Huppi (**A**), and by FA Esteves (**AA**). Abbreviations: **A2**, abdominal segment II; **A3**, abdominal segment III.

**Figure 31.**
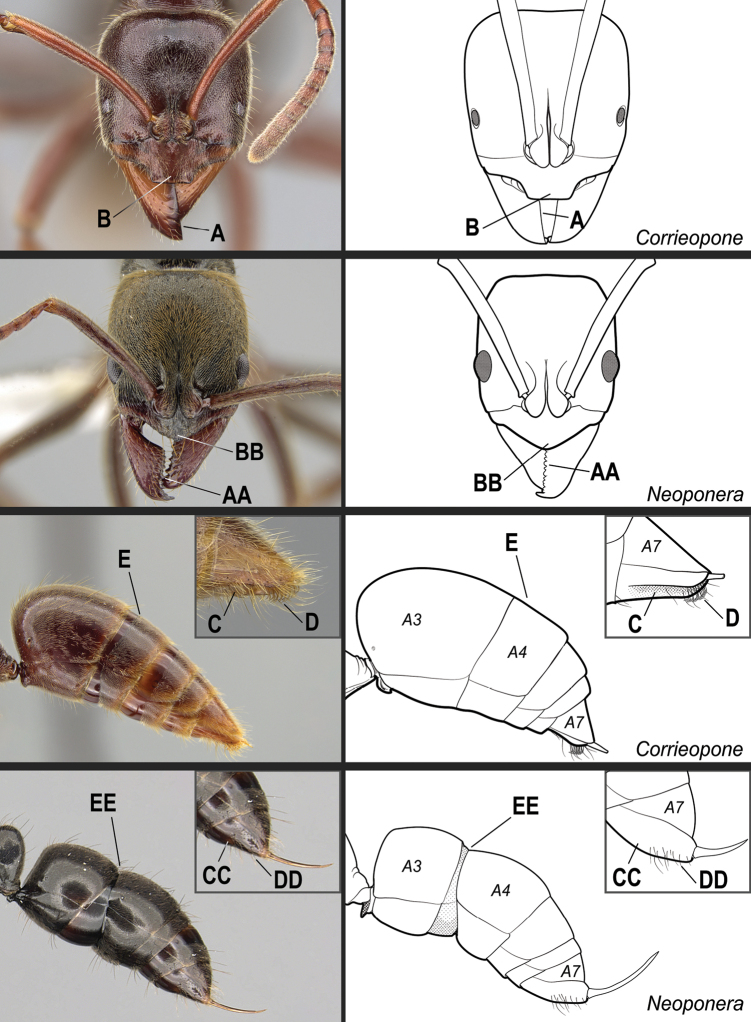
Identification key, couplet 14 **A, B***Corrieoponenouragues*, holotype, worker (CASENT0830464) **AA, BB***Neoponeraemiliae* (JTLC000015100) **C, D, E***Corrieoponenouragues*, paratypes, worker caste (CASENT0830465; CASENT0645962, insert) **CC, DD, EE***Neoponerafauveli*, worker (CASENT0249142). Images by W Lee (**A, B**), JT Longino (**AA, BB, E**), FA Esteves (**C, D**), and R Perry (**CC, DD, EE**); available at AntWeb.org. Illustrations by FA Esteves (**A–E**), and modified from artwork by Jessica Huppi (**AA–EE**). Abbreviations: **A3**, abdominal segment III; **A4**, abdominal segment IV; **A7**, abdominal segment VII.

**Figure 32.**
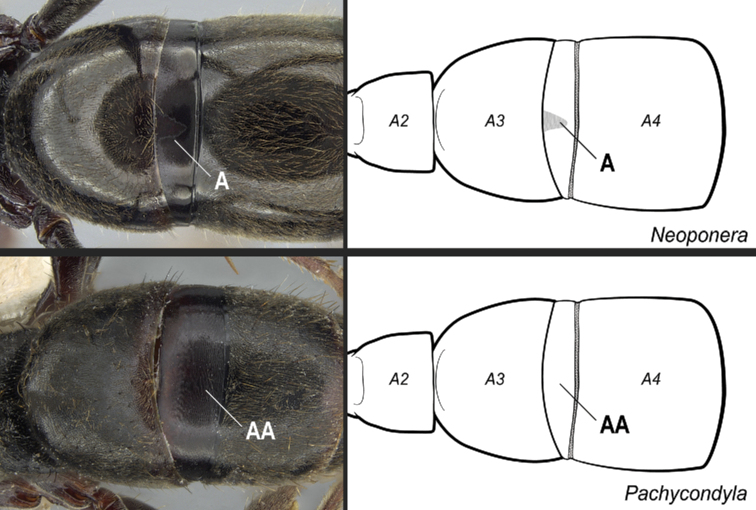
Identification key, couplet 15 **A***Neoponerafauveli*, worker (CASENT0428712) **AA***Pachycondyla* indet., worker (CASENT0006090). Images by FA Esteves; available at AntWeb.org. Illustrations modified from artwork by Jessica Huppi. Abbreviations: **A2**, abdominal segment II; **A3**, abdominal segment III; **A4**, abdominal segment IV.

**Figure 33.**
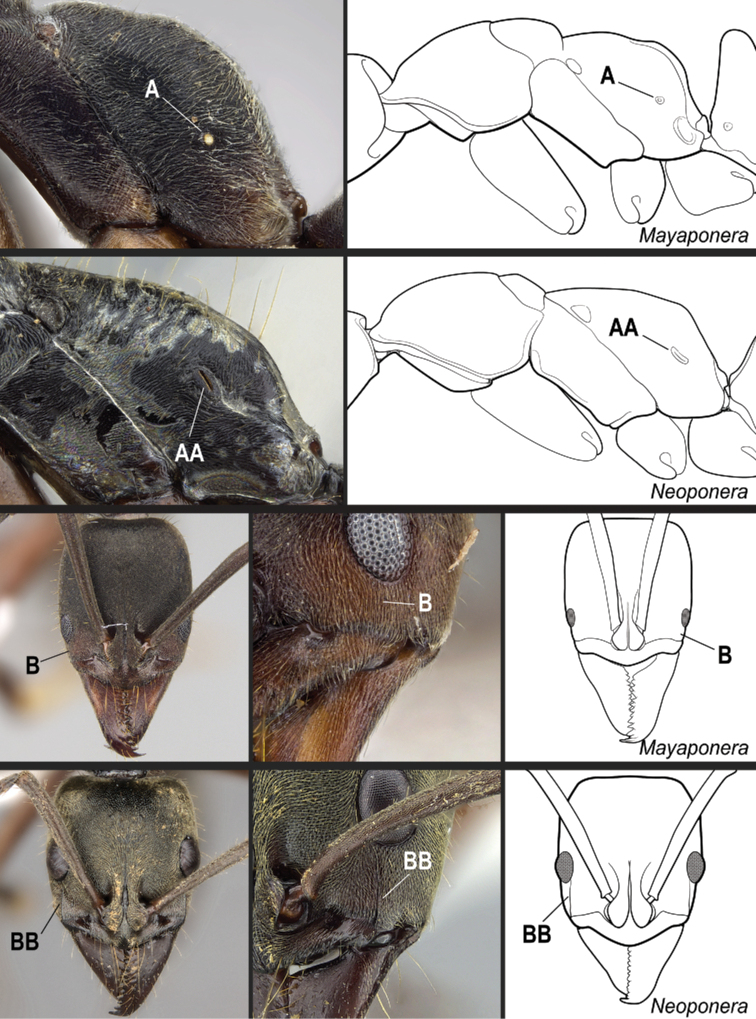
Identification key, couplet 16 **A***Mayaponeraconstricta* (CASENT0643470) **AA***Neoponerabugabensis* (CASENT0217570) **B***M.constricta* (CASENT0845824, left image; CASENT0643470, middle image) **BB***N.bugabensis* (CASENT0217570, left and middle images). Images by FA Esteves (**A, AA; B, BB**, middle images), W Lee (**B**, left image), and W Ericson (**BB**, left image); available at AntWeb.org. Illustrations modified from artwork by Jessica Huppi.

**Figure 34.**
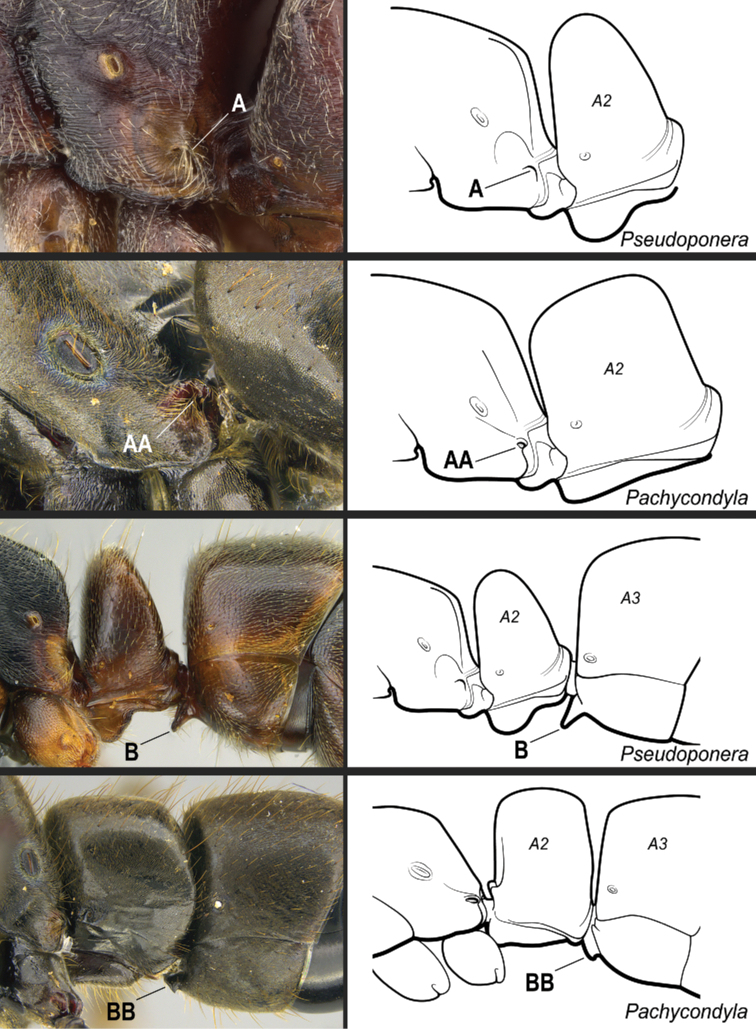
Identification key, couplet 17 **A***Pseudoponerastigma*, worker (CASENT0922570) **AA, BB***Pachycondylacrassinoda*, worker (CASENT0830390) **B***Pseudoponeragilberti*, worker (CASENT0828638). Images by FA Esteves; available at AntWeb.org. Illustrations modified from artwork by Jessica Huppi. Abbreviations: **A2**, abdominal segment II; **A3**, abdominal segment III.

**Figure 35.**
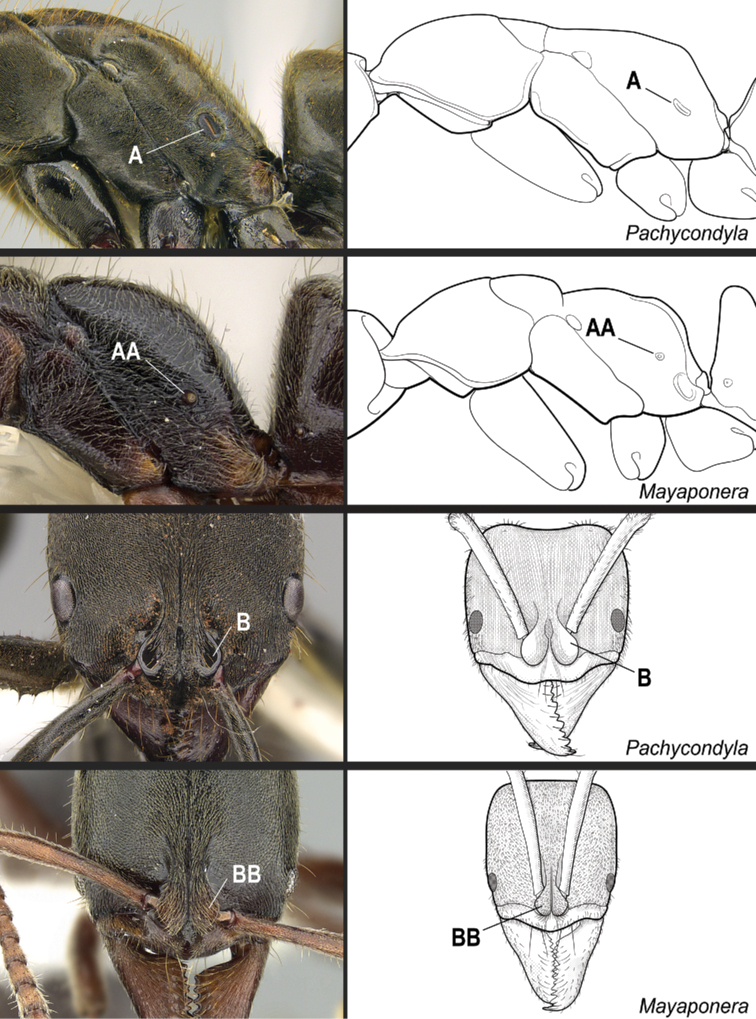
Identification key, couplet 18 **A***Pachycondylacrassinoda*, worker (CASENT0830390) **AA***Mayaponeraconicula*, paratype, worker (CASENT0923099) **B***P.striata*, worker (CASENT0923096) **BB***M.pergandei*, worker (CASENT0249156). Images by FA Esteves (**A**), W Lee (**AA, B**), and R Perry (**BB**); available at AntWeb.org. Illustrations modified from artwork by Jessica Huppi.

**Figure 36.**
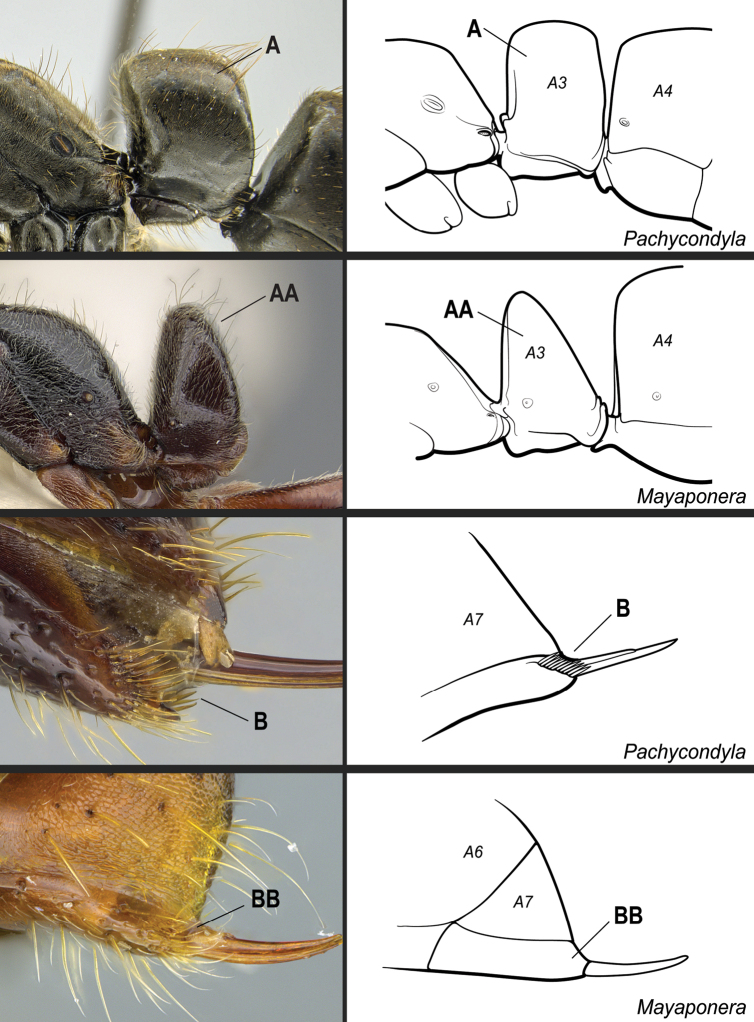
Identification key, couplet 18 **A***Pachycondylacrassinoda*, worker (CASENT0830390) **AA***Mayaponeraconicula*, paratype, worker (CASENT0923099); petiole disarticulated from gaster **B***P.lattkei*, paratype, worker (CASENT0217562) **BB***M.cernua*, worker (CASENT0923098). Images by FA Esteves (**A, B, BB**) and W Lee (**AA**); available at AntWeb.org. Illustrations modified from artwork by Jessica Huppi (**A**), by FA Esteves (**AA**), and Jessica Huppi (**B, BB**). Abbreviations: **A3**, abdominal segment III; **A4**, abdominal segment IV; **A6**, abdominal segment VI; **A7**, abdominal segment VII.

#### ﻿ Corrieopone

Taxon classificationAnimaliaHymenopteraFormicidae

685DD833-5F78-51DF-8D1D-9055218AB18C

http://zoobank.org/2B32773A-92E1-41B2-BC74-EC0B8ECD4A0A

Figs 7 , 8 , 37 , 38 , 39 , 40 , 41 , 42 , 43 , 44 , 52A , 53 , 54 , 55 , 56 , 57

##### Type species.

*Corrieoponenouragues* sp. nov., by present designation.

##### Diagnosis based on workers.

**Figure 37.**
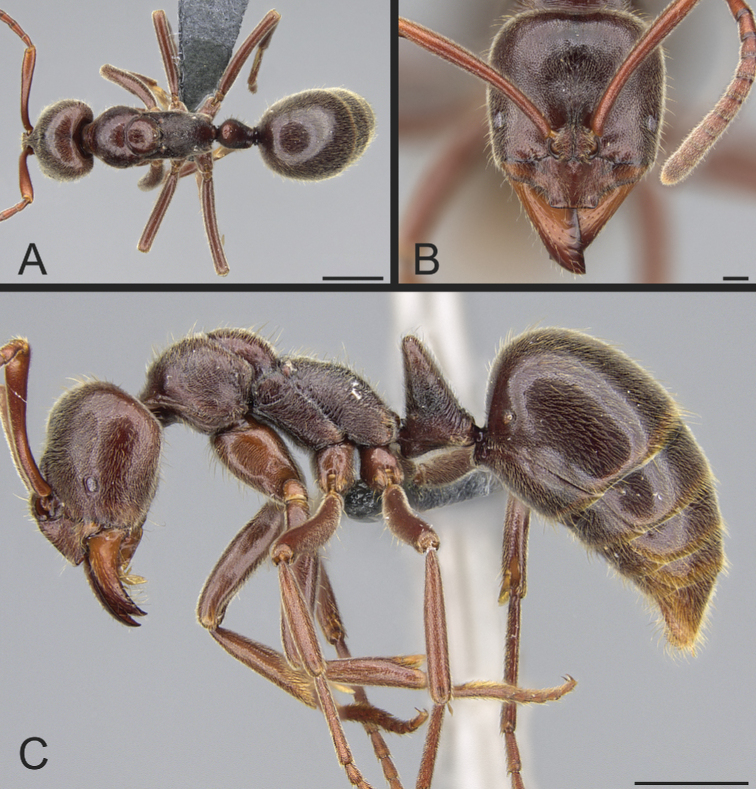
*Corrieoponenouragues*, holotype, worker (CASENT0830464) **A** body in dorsal view **B** head in full-face view **C** body in profile. Images by W Lee; available at AntWeb.org.

**Figure 38.**
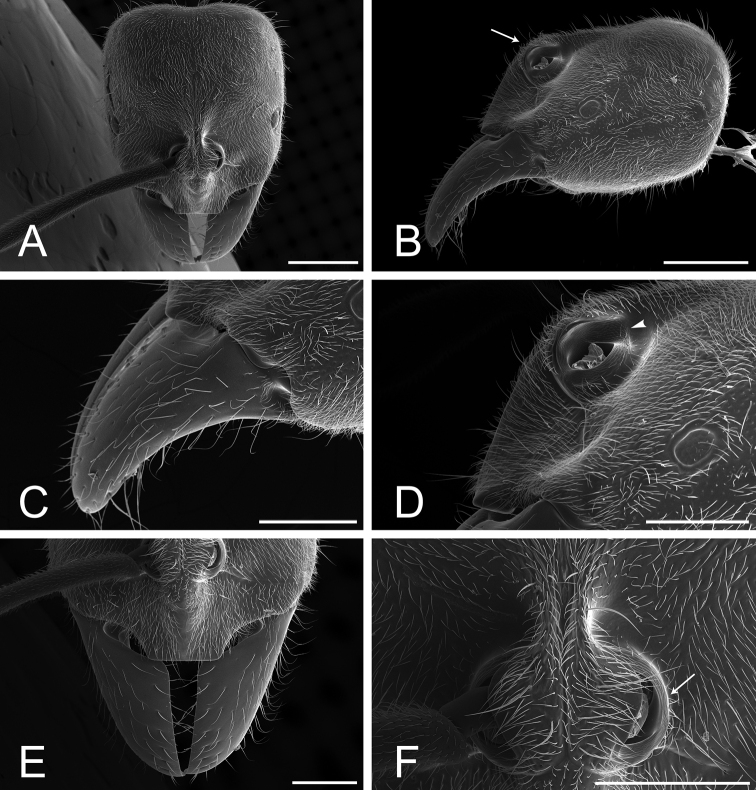
*Corrieoponenouragues*, paratype, worker (CASENT0872031) **A** head in full-face view **B** head in profile; arrow indicates the torular lobes atop a prominence formed by the clypeal median area and the frontal carinae **C** dorsolateral face of the mandible **D** clypeus and torulus in profile; the arrowhead indicates the discontinuity between the posterior margins of the median and lateral arches of the torulus **E** dorsal face of clypeus and mandibles **F** torular lobes in dorsal view; the arrow points to the exposed lateral arch of the torulus. Images by FA Esteves; available at AntWeb.org. Scale bars: 0.5 mm (**A, B**); 0.3 mm (**C, D, E, F**).

**Figure 39.**
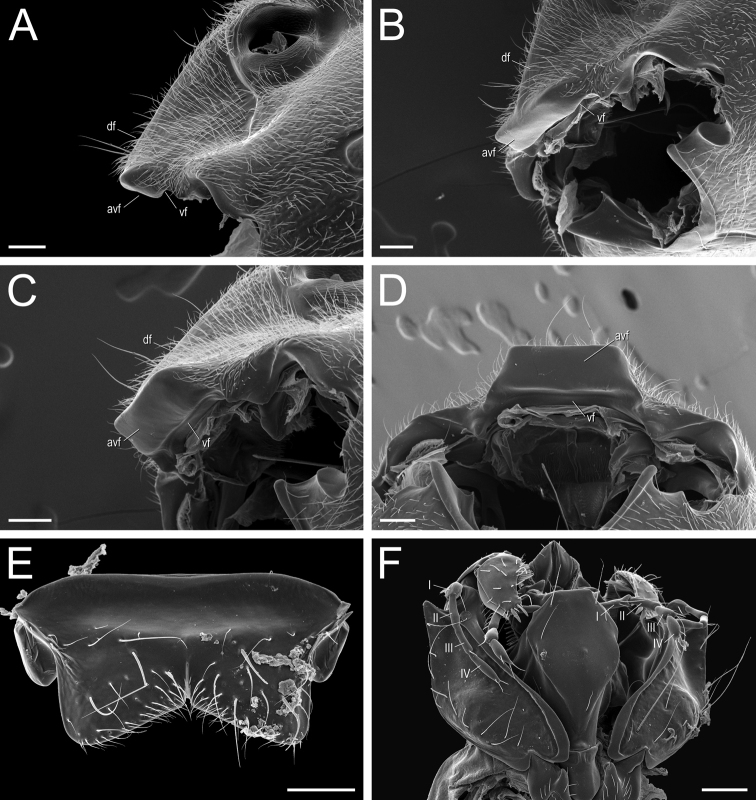
*Corrieoponenouragues*, paratype, worker caste **A** clypeus in profile; mandible removed **B** anterior part of the head in oblique anterior view; mandibles and maxilla-labial complex removed **C** clypeus in oblique anterior view; mandibles and maxilla-labial complex removed **D** anterior part of the head in ventral view; mandibles and maxilla-labial complex removed **E** outer face of the labrum; basal towards the top of the image **F** outer face of the maxillolabial complex; basal towards the bottom of the image; Roman numerals indicate the count of maxillary and labial palpomeres. Specimens imaged: CASENT0923158 (**E, F**); CASENT0872031 (**A–D**). Images by FA Esteves; available at AntWeb.org. Abbreviations: **avf**, clypeal anteroventral face; **df**, clypeal dorsal face; **vf**, clypeal ventral face. Scale bars: 0.1 mm.

Medium-sized, slender Neotropical ants (TL 6.5–7.1 mm; Fig. 37) with characters of Ponerinae (as in Fisher and Bolton 2016) and Ponerini (as in Schmidt and Shattuck 2014), in addition to the following (asterisks indicate putative autapomorphies):

1. Mandibles triangular, with distinct masticatory and basal margins; inserted at the anterolateral corners of the head (Fig. 38A, E).

2. Mandibles edentate (Fig. 38A, E).

3. Mandible devoid of any pit or sulcus: basolateral and dorsal pits and dorsolateral and dorsomasticatory sulci absent (Fig. 38A–C, E).

4. * Clypeus complex: In dorsal view, clypeus projected anteromedially as a broad, truncated prominence, overhanging the basal margins of the mandibles, and overlapping the basal portion of the masticatory margins of fully closed mandibles; anterior margin of the clypeal projection approximately as wide as the distance between the lateral arches of the toruli, devoid of stout setae or additional protrusions (Figs 37B, 38A, E). In profile, clypeal projection with a broad anteroventral face (avf, Fig. 39A–D), which extends ventroposteriorly (i.e., obliquely) from the clypeal dorsal face in almost 90 degrees; the ventralmost point of this anteroventral face meets the “true” clypeal ventral face (= surface of the clypeal infold in Boudinot et al. 2021; vf, Fig. 39A–D). In anteroventral view, clypeal anteroventral face subrectangular (avf, Fig. 39D). Median area of the clypeus bulging (Fig. 37A, E); seen in profile, it ascends steeply from the clypeal anterior margin to the torular lobes, with posterior portion slightly convex (Fig. 38B, D).

5. In dorsal view, torular lobes closely approximated.

6. In dorsal view, torular lobes medium- to small-sized: not concealing the lateral arches of the toruli (Fig. 38F). Median and lateral arches of the torulus with discontinuous posterior margins (Fig. 38D).

7. In profile, torular lobes located at the dorsalmost part of a prominence formed by the clypeal median area and the frontal carinae (Fig. 38B).

8. Compound eyes small and located immediately anterior to the midline of the head (Fig. 38A, B, D).

9. Ocelli absent (Fig. 38A).

10. Labrum apically bilobed; with a long, acute cleft at the midpoint of its apical margin. Lobes broadly rounded apicolaterally and unarmed (Fig. 39E).

11. Palpal formula: 4,4 (four maxillary, four labial palpomeres; Fig. 39F).

12. Mesonotum rounded, dome-shaped in profile (Fig. 40A), rounded in dorsal view (Fig. 37A).

13. Notopleural suture distinct (Fig. 40A, B).

14. Metanotal sulcus deeply impressed (Fig. 40A).

15. Mesopleuron in profile divided into anepisternum and katepisternum (Fig. 40A, B).

16. Metathoracic spiracle concealed by a spiracular lobe (Fig. 40B).

17. Orifice of the metapleural gland round, opening posterolaterally on the metapleuron, with its ventral margin atop the posteriormost portion of the metapleural carina (Fig. 40C, D).

18. Metapleural longitudinal flange absent (Fig. 40C, D).

19. Propodeal dorsum devoid of a median longitudinal groove or impression.

20. Propodeum unarmed: without dorsoposterior projections (Fig. 40A).

21. In profile, propodeal lobe round, not surpassing posteriorly the dorsoposterior-most point of the rim of the propodeal foramen (Fig. 40C).

22. Propodeal spiracle slit-shaped (Fig. 40C).

23. Mesosternal process bidentate; metasternal process bilobate, long (Fig. 40F).

24. Metacoxal cavities open; cavities tightly encircled by cuticle, but cuticular annulus not fused (Fig. 40E; see comment in the following section).

25. Calcar of strigil with a basoventral lamella (Fig. 41A, B).

26. Probasitarsus with anterior and ventral faces densely vested with spatulate-costate setae (Fig. 41A), except for the shorter, spatulate-bicuspid setae present on the area immediately anterior to the comb of strigil (Fig. 41B).

27. Row of stout, spine-like setae present on the posterior face of the probasitarsal notch, parallel to the comb of strigil (Fig. 41B).

28. Two well-developed mesotibial spurs: anterior spur simple, posterior spur serrate (Fig. 41C, D).

29. Apparent metatibial gland cuticular patch present on the apicoposterior face of the metatibia, next to the posterior metatibial spur (Fig. 41E).

30. Two well-developed metatibial spurs: anterior spur simple, posterior spur pectinate (Fig. 41F).

**Figure 40.**
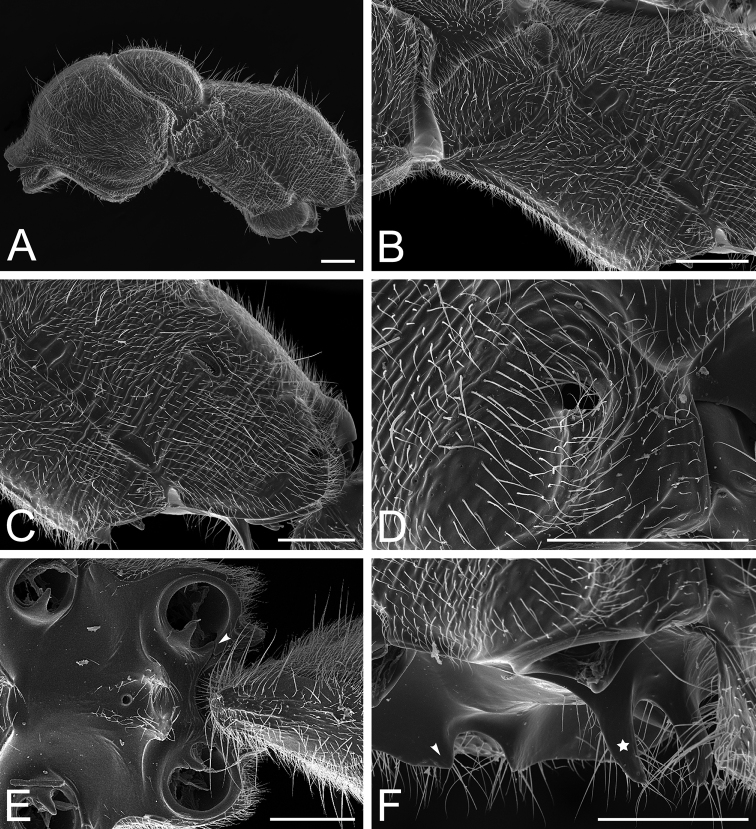
*Corrieoponenouragues*, paratypes, worker caste **A** mesosoma in profile **B** mesopleuron and part of metapleuron and propodeum in profile **C** katepisternum, metapleuron, and propodeum in profile **D** metapleural gland orifice in posterolateral view **E** metapleuron (and posterior part of the mesopleuron) in ventral view; arrowhead indicates the unfused annulus around the metacoxal cavity **F** mesosternal and metasternal processes in posterolateral view, highlighted by arrowhead and star, respectively. Specimens imaged: CASENT0872031 (**A**); CASENT0923158 (**B–F**). Images by FA Esteves; available at AntWeb.org. Scale bars: 0.2 mm.

31. Stout, spine-like setae absent from the dorsal face of mid- and hindlegs (Fig. 42A, B).

32. Ventral faces of the second, third, and fourth tarsomeres of fore-, mid-, and hindlegs with a paired row of stout, spine-like setae skirting the midline of each segment (Fig. 42C–E).

**Figure 41.**
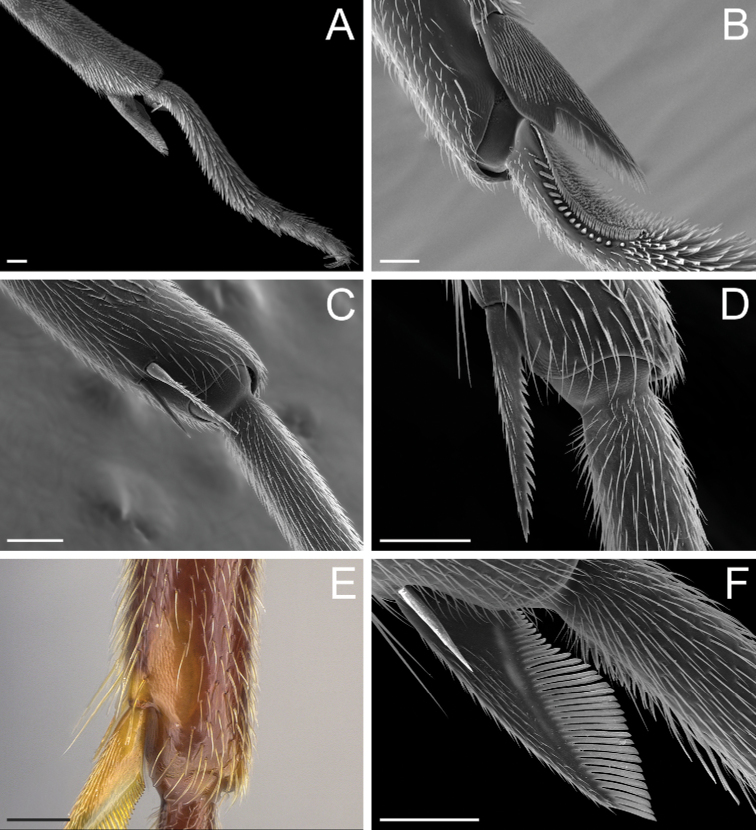
*Corrieoponenouragues*, worker caste **A** protibia and protarsus in anterior view **B** protibial apex and probasitarsus in posteroventral view **C** mesotibial apex and basal portion of the mesobasitarsus in ventral view **D** mesotibial apex and basal portion of the mesobasitarsus in posterior view **E** metatibial apex in posterior view; note the lighter, colliculate cuticular patch next to the posterior metatibial spur **F** metatibial spurs in anterior view. Specimens imaged: holotype CASENT0830464 (**E**); paratype CASENT0923158 (**A, C, D, F**); paratype CASENT0872031 (**B**). Images by FA Esteves (**A–D, F**) and M Esposito (**E**); available at AntWeb.org. Scale bars: 0.1 mm.

**Figure 42.**
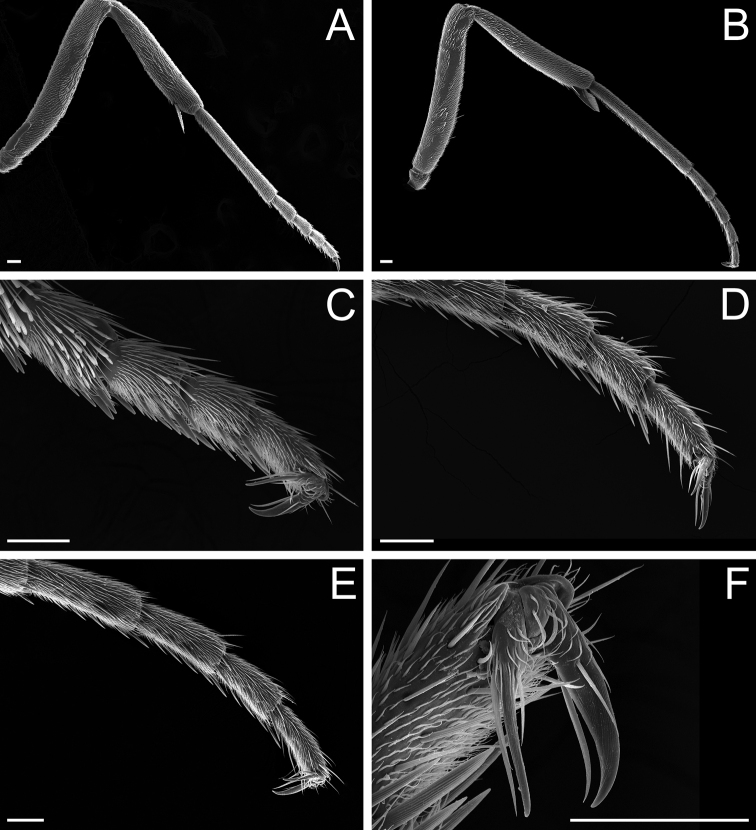
*Corrieoponenouragues*, paratypes, worker caste **A** midleg (minus coxa) in posterior view **B** hindleg (minus coxa) in anterior view **C** apex of the foreleg in anteroventral view **D** apex of the midleg in anteroventral view **E** apex of the hindleg in anteroventral view **F** apicalmost tarsomere and pretarsus of the foreleg in anteroventral view. Specimens imaged: CASENT0923158 (**A–C, E, F**); CASENT0872031 (**B**). Images by FA Esteves; available at AntWeb.org. Scale bars: 0.1 mm.

**Figure 43.**
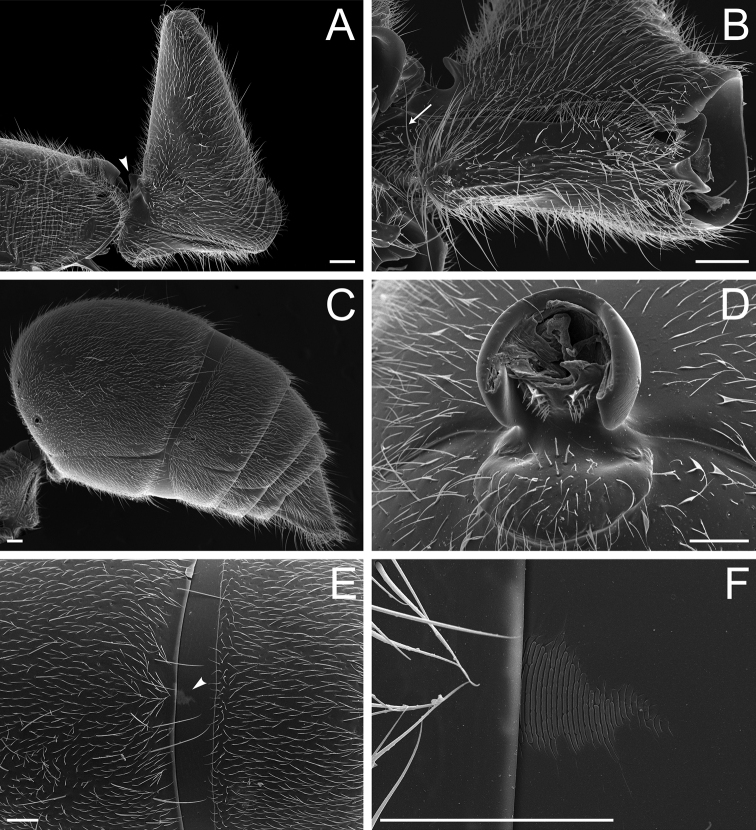
*Corrieoponenouragues*, paratypes, worker caste **A** petiole in profile; arrowhead indicates the anteroventral lateral carina of the petiolar tergite **B** petiolar sternite in postlateroventral view; arrow indicates the proprioceptor zone on the anterior disc of the sternite **C** gaster in profile **D** helcium and prora in anterior view **E** the posterior portion of the abdominal tergite III and the anterior portion of the abdominal tergite IV in dorsal view; arrowhead indicates the stridulitrum **F** stridulitrum on abdominal pretergite IV. Specimens imaged: CASENT0923158 (**A, B, D**); CASENT0872031 (**C, E, F**). Images by FA Esteves; available at AntWeb.org. Scale bars: 0.1 mm.

33. Pro-, meso-, and metapretarsi with simple claws (Fig. 42C–F).

34. Arolia indistinct (Fig. 42F).

35. Petiole sessile, with high, unarmed, conic, scale-like node (i.e., tergite narrow in profile and dorsal view; Figs 37A, C, 43A).

36. Petiolar tergite with anteroventral lateral carina (= lateral, dorsoventral carina in Ward 1990, character 29); posteroventral portion of tergite strigate (Fig. 43A).

37. Petiolar laterotergite distinct (Fig. 43A).

38. Proprioceptor zone on anterior disc of petiolar sternite shaped as a large circular area (Fig. 43B).

39. Petiolar sternite without posterior spatulate projection (Fig. 43B); articulation with helcium visible in ventral view.

40. Helcium infra-axial: positioned ventrad the midheight of the anterior face of abdominal segment III (Fig. 43C).

41. Prora present as a lip-shaped, transverse projection on the anterior portion of the abdominal poststernite III (Fig. 43C, D); projection not extending anteriorly to the area between the ventroposterior margins of helcial tergite (Fig. 43D).

42. Abdominal segment IV tubular: tergite and sternite with similar lengths; tergite not arched (Fig. 43C).

43. Presclerites of abdominal segment IV forming an even surface with postsclerites: girdling constriction absent (Fig. 43C).

44. Stridulitrum present on abdominal pretergite IV, small (Fig. 43E, F).

45. Pygidium devoid of stout, spine-like setae or spine-like microtrichia; dorsal face convex.

46. * Ventral face of hypopygium longitudinally concave (Fig. 44A, B). Longitudinal carina present at midline of concavity’s posterior portion, skirted laterally by stout, hook-shaped setae (Fig. 44C, D).

**Figure 44.**
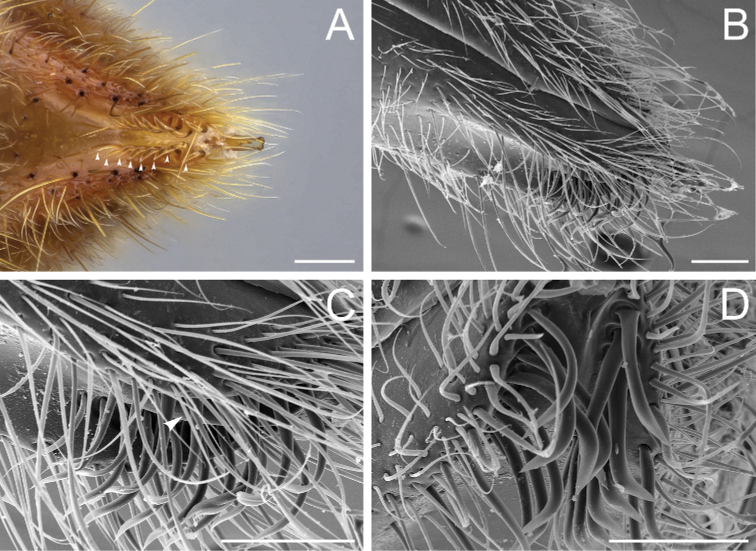
*Corrieoponenouragues*, worker caste **A** the concave ventral face of the hypopygium armed with stout, hook-shaped setae (see arrowheads) on its posteriormost portion **B** hypopygium in ventrolateral view **C** close-up of the median carina (see arrowhead) and hook-shaped setae of the hypopygium in ventrolateral view **D** hook-shaped setae of the hypopygium in posteroventral view. Specimens imaged: holotype CASENT0830464 (**A**); paratype CASENT0923158 (**B–D**). Images by FA Esteves; available at AntWeb.org. Scale bars: 0.1 mm.

47. Hypopygium posterolateral region without stout, spine-like setae or spine-like microtrichia.

***Comments on worker characters***

The enumeration below corresponds to character numbers presented above.

1. The mandibles articulate with the anterolateral corners of the head in virtually all Ponerinae and are either triangular or subtriangular in most genera (*N* = 29). Other mandibular shapes with little intrageneric variation are the oblique (*Boloponera*, *Buniapone*, *Dinoponera*, *Iroponera*, *Plectroctena*, and *Promyopias*), pitchfork-like (*Belonopelta*, *Emeryopone*, *Thaumatomyrmex*), elongate sub-oblique (*Streblognathus*), and scythe-shaped (*Harpegnathos*). The shape of the mandibles varies from triangular to elongate-triangular in *Centromyrmex*; from triangular to subtriangular, to oblique in *Cryptopone*; from subtriangular to oblique, to falcate, to bizarre forms in-between in *Leptogenys* and *Myopias*; from sub-oblique to falcate in *Psalidomyrmex*; and from subtriangular to oblique in *Simopelta*. *Anochetus* and *Odontomachus* are the only ponerines in which the mandibles insert near the midline of the anterior margin of the head.

2. The mandible of *Corrieopone* is completely edentate (i.e., devoid of any teeth, denticle, or projected apex). To our knowledge, this condition is virtually absent in other Ponerinae, apart from some species of *Leptogenys* (see Arimoto and Yamane 2018) and *Platythyrea* (see Fisher and Bolton 2016).

3. Ponerine may present mandibles ornamented with pits and sulci, which are relatively good diagnostic characters to genera.

**Figure 45.**
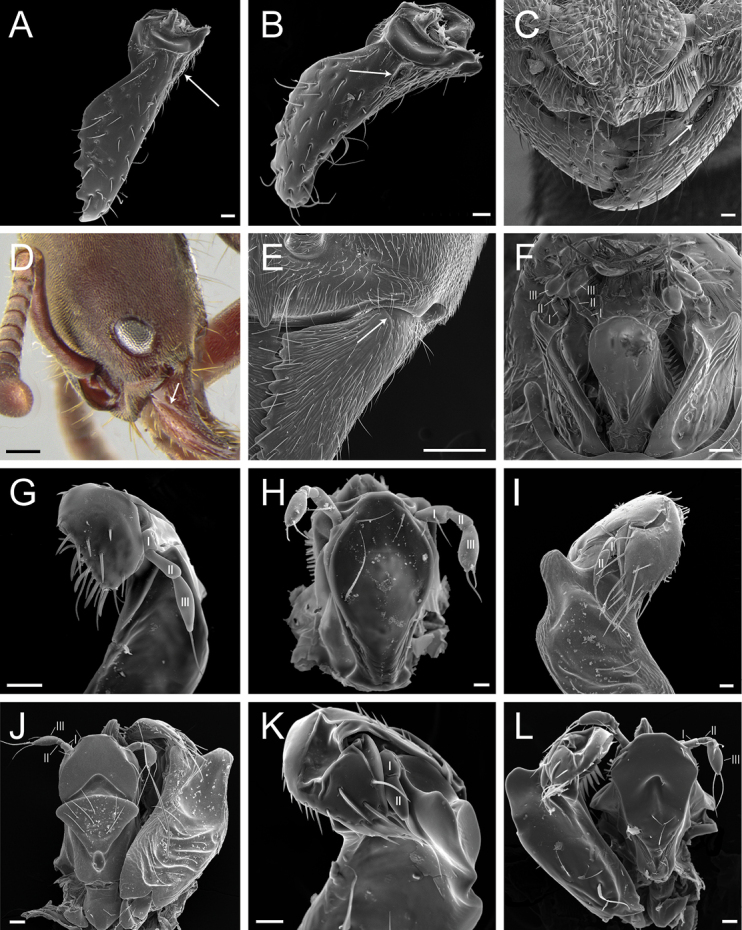
**A** left mandible of *Fisheroponeambigua* in dorsal view; worker (CASENT0906209); arrow indicates the basolateral pit **B** left mandible of *Fisheroponeambigua* in postdorsolateral view (CASENT0906209); arrow indicates the basolateral pit **C** anterior part of the head of *Iroponeraodax* in dorsal view; worker (ANTWEB1008537); arrow indicates the dorsal pit **D** head of *Rasoponepanamensis* in profile; worker (CASENT0644543); arrow indicates the dorsolateral sulcus **E** anterior portion of the clypeus and posterior portion of the mandible of *Austroponeracastanea* in dorsolateral view; worker (CASENT0097796); arrow indicates the short and shallow dorsolateral sulcus **F** the expanded maxillolabial complex of *Dolioponerafustigera* in ventral view; worker (CASENT0411307); Roman numerals indicate the count of maxillary and labial palpomeres **G** apical portion of the outer face of the left maxilla of *Fisheroponeambigua* (CASENT0906209); Roman numerals indicate the count of maxillary palpomeres **H** outer face of the labium of *Fisheroponeambigua* (CASENT0906209); Roman numerals indicate the count of labial palpomeres **I** apical portion of the outer face of the right maxilla of *Loboponeraobeliscata*; worker (ANTWEB1008545); Roman numerals indicate the count of maxillary palpomeres **J** outer face of the labium and left maxilla of *Loboponeraobeliscata* (ANTWEB1008545); Roman numerals indicate the count of labial palpomeres **K** apical portion of the outer face of the left maxilla of *Boloponeraikemkha*; paratype, ergatoid queen (CASENT0254321); Roman numerals indicate the count of maxillary palpomeres **L** outer face of the labium and right maxilla of *Boloponeraikemkha* (CASENT0254321); Roman numerals indicate the count of labial palpomeres. Images by FA Esteves (**A, B, E–H, K, L**), RA Keller (**C, I, J**), and JT Longino (**D**); available at AntWeb.org. Scale bars: 0.02 mm (**A–C, F, G, I, J–L**); 0.2 mm (**D, E**); 0.01 mm (**H**).

3.1. The basolateral pit (= basal pit in Fisher and Bolton 2016) is a round to oblong impression on the basal portion of the lateral face of the mandible. It occurs in most *Brachyponera* species (excluding species from Borneo, Bali, Krakatau, and Sumatra, according to Yamane 2007), *Cryptopone* [except for *C.guianensis* and, based on its original description, *C.mirabilis* (Mackay & Mackay), as far as we know], and *Euponera* (see Schmidt and Shattuck 2014). The pit is also present in *Fisheroponeambigua* (Fig. 45A, B), which disagrees with Schmidt and Shattuck (2014) and Fisher and Bolton (2016).

3.2. The dorsal pit resembles the basolateral pit, although more elongated and impressed on the dorsal face of the mandible. In Ponerinae, it is only present in *Dolioponera*, *Euponerafossigera* Mayr (see Mayr 1901), *Hagensia*, and *Iroponera* (Fig. 45C; contrary to Schmidt and Shattuck 2014).

3.3. The dorsolateral sulcus runs obliquely along the mandible, from the basal portion of the dorsal face towards the lateral face. It is widespread among the Ponerinae and may be shallowly or deeply impressed, restricted to the basal portion of the mandible, or present along almost the entire lateral face of the mandible. The sulcus is consistently present in species of *Asphinctopone*, *Boloponera*, *Buniapone*, *Centromyrmex*, most (perhaps all) *Ectomomyrmex*, *Feroponera*, *Loboponera*, *Myopias*, *Odontoponera*, *Paltothyreus*, *Phrynoponera*, *Plectroctena*, *Promyopias*, *Psalidomyrmex*, *Pseudoponera*, and *Streblognathus* (see Suppl. material 3: Table S3; Schmidt and Shattuck 2014; Fisher and Bolton 2016). On the other hand, the presence of this character varies among species of other genera, such as *Bothroponera* (present in *B.crassa*, absent in *B.pachyderma*), *Dinoponera* (weakly present in *D.lucida*; absent in *D.longipes*), *Leptogenys* (see Bolton 1975), *Mesoponera* (only present in *M.subiridescens*), *Neoponera* [present in *N.fauveli* (Emery), weakly impressed in *N.apicalis*, absent in *N.fisheri*], *Platythyrea* (present in *P.punctata*, absent in *P.turneri*), and *Pseudoneoponera* (present in *P.porcata*, absent in *P.denticulata*). Contrary to Schmidt and Shattuck (2014), the sulcus is distinct in all *Rasopone* species examined here (Fig. 45D); although short and constrained to the basal region of the mandibles, it is also present in *Austroponeracastanea* (Fig. 45E). Among the *Pachycondyla* species examined, the sulcus is a shallow and short basal impression save in *P.lenis*, where it is absent. In *Euponerasikorae* (and in all other Malagasy species, according to Rakotonirina and Fisher 2013), the lateral face of the mandible bears a longitudinal sulcus that runs apicad from the lateral margin of the dorsal mandibular articulation. Whether this sulcus is present in *Euponera* species occurring in other bioregions remains unknown, but it is absent in at least two Afrotropical species, *E.brunoi* and *E.sjostedti*.

3.4. *Plectroctena* species present a sulcus that skirts the mandibular masticatory margin dorsally (Fisher and Bolton 2016), which we refer to as the dorsomasticatory sulcus.

Among the specimens examined, the following taxa present mandibles devoid of any pit or sulcus, like *Corrieopone*: *Anochetusangolensis*, *A.emarginatus*, *Belonopeltadeletrix*, *Bothroponeracariosa*, *B.pachyderma*, *B.talpa*, *Cryptoponeguianensis*, *Diacammaceylonense*, *Dinoponeralongipes*, *Emeryoponebuttelreepeni*, *Harpegnathossaltator*, *Hypoponerapunctatissima*, *Mayaponera*, *Megaponeraanalis*, *Mesoponeraambigua*, *M.australis*, *M.caffraria*, *M.elisaerotundata*, *M.melanariamacra*, *M.papuana*, *M.rubra*, *Neoponeracommutata*, *N.fisheri*, *N.laevigata*, *N.luteola*, *N.verenae*, *N.villosa*, *Odontomachusbauri*, *Ophthalmoponeberthoudi*, *Pachycondylalenis*, *Parvaponeradarwiniimadecassa*, *Platythyreaturneri*, *Poneraalpha*, *P.pennsylvanica*, *Simopeltaoculata*, *S.transversa*, *Thaumatomyrmexfraxini*, and *T.zeteki*.

6. We assessed the size of the torular lobes across taxa examined according to the degree of connection between median and lateral arches of torulus (as in Keller 2011, character 08). Among material examined, the lobes are anteriorly and posteriorly continuous with the lateral torular arches in taxa whose antennal sockets are largely exposed in full-face view (*Belonopelta*, *Leptogenys*, and *Ophthalmopone*). On the other extreme, hypertrophied lobes are anteriorly and posteriorly discontinuous with the lateral arches of the torulus (as in *Boloponera*, *Bothroponera* sensu stricto group, *Loboponera*, *Platythyreapunctata*, *Plectroctena*, and *Psalidomyrmex*). Like *Corrieopone*, most ponerines present intermediate-sized torular lobes that are anteriorly continuous and posteriorly discontinuous with respective lateral arches. In this latter state, the lobes may conceal entirely or partially the lateral arches of the torulus.

For the record, the torular lobes in *Bothroponerasulcata* species-group members (sensu Schmidt and Shattuck 2014) resemble those of *Corrieopone*, except for *B.henryi* (Donisthorpe) (see specimen CASENT0902482) and *B.zumpti* Santschi (see CASENT0922370), where the lobes are hypertrophied as in the *Bothroponera* sensu stricto group.

9. Ocelli are invariably present on *Harpegnathos* workers; it is absent in the worker caste of other Ponerinae, apart from occasional workers.

11. A count of four maxillary and four labial palpomeres is consistently present in *Buniapone*, *Dinoponera*, *Hagensia*, *Harpegnathos*, *Mayaponera* (note that we did not examine *M.longidentata*), *Megaponera*, *Odontoponera*, *Ophthalmopone*, *Paltothyreus*, *Phrynoponera*, *Promyopias*, *Streblognathus* (see Suppl. material 3: Table S3; Bolton 2003; Fisher and Bolton 2016). We are hesitant to affirm that this is also the case in *Neoponera*, *Pachycondyla*, *Pseudoneoponera*, and *Rasopone*. These characters usually have been neglected in taxonomic reviews and descriptions of new species, and we did not examine every species in these genera. Yet, in every species we did examine (*Neoponeraaenescens*, *N.apicalis*, *N.bugabensis*, *N.carinulata*, *N.commutata*, *N.crenata*, *N.curiosa*, *N.dismarginata*, *N.fisheri*, *N.foetida*, *N.globularia*, *N.insignis*, *N.inversa*, *N.laevigata*, *N.luteola*, *N.moesta*, *N.obscuricornis*, *N.schoedli*, *N.striadinodis*, *N.unidentata*, *N.verenae*, *N.villosa*, *Pachycondylacrassinoda*, *P.harpax*, *P.impressa*, *P.lattkei*, *P.lenis*, *P.procidua*, *P.striata*, *Pseudoneoponeraporcata*, *P.tridentata*, *Rasoponecostaricensis*, *R.cryptergate*s, *R.cubitalis*, *R.guatemalensis*, *R.panamensis*, *R.pluviselva*, and *R.*politognatha), the palpal formula was 4,4. The count is also 4,4 in some species of genera with variable palpal formulae, such as *Anochetus*, *Bothroponera*, *Leptogenys*, *Mesoponera*, and *Myopias* (see Suppl. material 3: Table S3; Willey and Brown 1983; Bolton 2003; Fisher and Bolton 2016).

**Figure 46.**
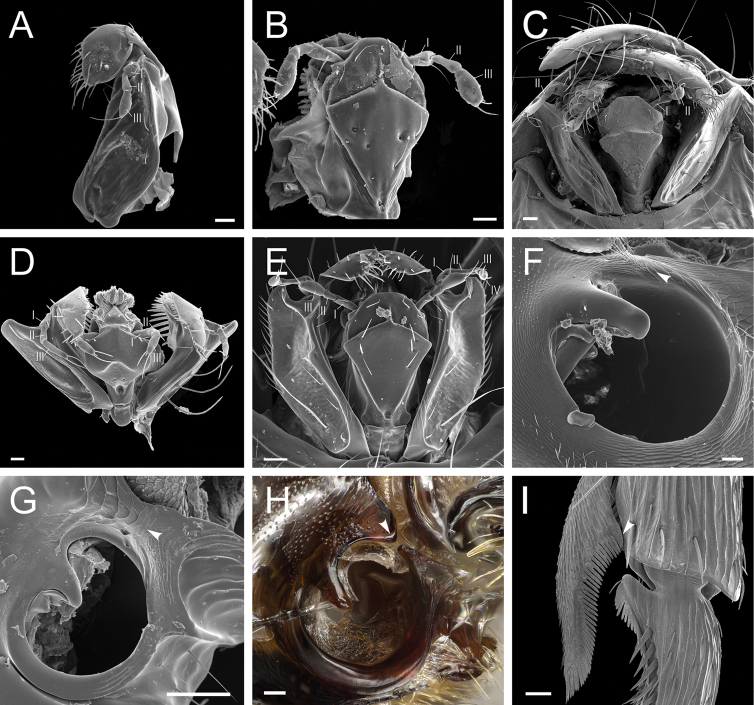
**A** outer face of the left maxilla of *Cryptoponehartwigi*; worker (CASENT0251956); Roman numerals indicate the count of maxillary palpomeres **B** outer face of the labium of *Cryptoponehartwigi* (CASENT0251956); Roman numerals indicate the count of labial palpomeres **C** expanded maxillolabial complex of *Simopeltatransversa* in ventral view; worker (ANTWEB1008589); Roman numerals indicate the count of maxillary and labial palpomeres **D** maxillolabial complex of *Thaumatomyrmexfraxini* in ventroapical view; worker (ANTWEB1008597); Roman numerals indicate the count of maxillary and labial palpomeres **E** expanded maxillolabial complex of *Parvaponeradarwiniimadecassa* in ventral view; worker (CASENT0389498); Roman numerals indicate the count of maxillary and labial palpomeres **F** right metacoxal cavity of *Platythyreacribrinodis* in ventral view; worker (CASENT0778160); arrowhead indicates the fused annular cuticle encircling the cavity externally **G** right metacoxal cavity of *Myopiasdarioi* in ventral view; paratype, worker (CASENT0810080); arrowhead indicates the fused annular cuticle encircling the cavity externally **H** right metacoxal cavity of *Phrynoponerapulchella* in ventral view; worker (CASENT0217034); arrowhead indicates the annular gap around the cavity **F** protibial apex and basal region of the probasitarsus of *Loboponeraobeliscata* in posterior view; worker (ANTWEB1008545); arrowhead indicates the minute basoventral lamella of the calcar. Images by FA Esteves (**A, B, E–H**) and RA Keller (**C, D, I**); available at AntWeb.org. Scale bars: 0.02 mm (**A, B, D**); 0.03 mm (**C**); 0.04 mm (**E–I**).

While conducting this study, we noticed that the palpal formula of some taxa was incorrectly reported or missing in the pertinent literature. Thus we correct or update the record here. Contrary to Fisher and Bolton (2016), the palpal formula in *Dolioponerafustigera* is 3,3 (not 2,2, as previously stated; Fig. 45F), and 3,3 in *Fisheroponeambigua* (not 3,2; Fig. 45G, H). Contrary to Bolton (2003), Keller (2011), and Fisher and Bolton (2016), the palpal formula in *Loboponeraobeliscata* is 2,3 (not 2,2; Fig. 45I, J); the formula in *L.vigilans* was correctly reported as 2,2 by these authors (verified on specimen CASENT0003102). We also report for the first time the palpal formula in *Boloponeraikemkha* (2,3; Fig. 45K, L), *Cryptoponehartwigi* (3,3; Fig. 46A, B), and *Simopeltatransversa* (2,2; Fig. 46C). The specimen of Thaumatomyrmexatrox examined by Keller (2011) was later determined to be *T.fraxini* by D’Esquivel et al. (2017) – note that the specimen is not in the list of material examined by the latter authors, but some SEM images taken by Keller (2011; specimen ANTWEB1008597) were used to illustrate the new taxon description. One detail not mentioned by D’Esquivel et al. (2017) was the palpal formula of *T.fraxini*, which is 3,3 (Fig. 46D; previously reported for *T.atrox* by Keller 2011). Finally, although Fisher and Bolton (2016) stated that the palpal formula in *Parvaponeradarwiniimadecassa* is unknown, Brown (1963) provided the correct count, which is 4,3 (Fig. 46E).

12–14. In *Corrieopone*, the mesonotum is dome-shaped in profile, with a round dorsal margin that is discontinuous with the outline of the pronotum (i.e., it is slightly higher than the pronotum). The promesonotum is much higher than the propodeum, and a deeply impressed metanotal sulcus separates the two. A distinct notopleural suture delimits the mesonotum from the mesopleuron. In dorsal view, the mesonotum is round. Taxa that bear some resemblance to *Corrieopone* in this combination of characters are: several *Anochetus* species (e.g., *A.altisquamis* Mayr, specimen CASENT0915154; *A.armstrongi* McAreavey, CASENT0902449; *A.brevis* Brown, CASENT0902439), *Asphinctopone*, *Austroponera* [except *A.rufonigra* (Clark), CASENT0249178], *Brachyponera*, *Euponerasikorae*, *Fisheroponeambigua*, *Hagensia*, some *Hypoponera* [e.g., *H.foreli* (Mayr), CASENT0173714; *H.herbertonensis* (Forel), CASENT0907320; *H.mesoponeroides* (Radchenko), CASENT0917250], some *Leptogenys* (e.g., *L.borivava* Rakotonirina & Fisher, CASENT0430091; *L.ixta*, *L.peruana*, *L.sonora*), *Mayaponera*, *Megaponeraanalis* (dome-shaped mesonotum in larger specimens, as CASENT0781129), most *Mesoponera* (e.g., *M.ambigua*, *M.australis*, *M.caffraria*, *M.elisaerotundata*, *M.melanaria*, *M.papuana*, *M.rubra*, *M.subiridescens*), several *Myopias* [e.g., *M.castaneicola* (Donisthorpe), CASENT0902520; *M.chapmani* Willey & Brown, CASENT0902533; *M.latinoda* (Emery), CASENT0270592], several *Neoponera* (e.g., *N.apicalis*, *N.aenescens*, *N.fisheri*, *N.schoedli*, *N.villosa*), some *Odontomachus* (e.g., *O.bauri*; *O.laticeps* Roger, CASENT0904008; *O.spissus* Kempf, CASENT0281868), *Odontoponeratransversa*, *Ophthalmopone*, *Rasoponerupinicola*, *R.cubitalis*, and *Streblognathus*.

16. The spiracular lobe is present in most ponerine genera; it is present in *Boloponeraikemkha* (CASENT0254321) and *B.vicans* (CASENT0401737), contrary to Fisher and Bolton (2016).

The lobe is absent in *Dolioponera*, *Fisheropone*, some *Loboponera* species, the Afrotropical and Malagasy *Hypoponera*, *H.punctatissima*, *Simopeltaoculata*, *S.transversa*, and *Thaumatomyrmexfraxini* (Suppl. material 3: Table S3; Fisher and Bolton 2016). Interestingly, while the lobe is absent in the Afrotropical *Cryptopone* (according to Fisher and Bolton 2016; here confirmed on *C.hartwigi*), it is present in the Neotropical *C.gilva* (ANTWEB1008514), *C.guianensis* (ANTWEB1008565), *C.holmgreni* (ECOFOG-IT14-0276-07), and *C.pauli* (CASENT0637806).

18. The metapleural longitudinal flange is a carina that extends along the metapleuron in profile, with its posterior end immediately dorsad the metapleural gland orifice. When well-developed, it projects laterad or ventrolaterad and may overhang the gland orifice (as defined by Keller 2011: character 62). In *Simopelta* species (ANTWEB1008589), this flange is strongly projected ventrolaterad and overlaps the gland orifice.

19. The propodeal dorsum presents a well-delimited, narrow, median longitudinal groove in *Psalidomyrmex* (Fisher and Bolton 2016; ANTWEB1008585). In addition, a vestigial longitudinal groove may be present on the propodeal dorsal face of specimens of the *Plectroctenaminor* group (see Bolton and Brown 2002). The propodeal dorsum is transversely concave along its entire length, or only posteriorly, in *Hagensia*, *Mayaponera* (CASENT0249137), several *Mesoponera* (CASENT0249194), and *Pseudoponera* (CASENT0923115).

20. Most ponerine genera present an unarmed propodeum. In profile, the propodeal dorsoposterior corner bears acute projections in several *Anochetus* species (e.g., CASENT0902431, CASENT0815182, CASENT0746783), *Phrynoponera* (CASENT0178230), *Streblognathus* (ANTWEB1008591), some *Platythyrea* (CASENT0281867, CASENT0900569, CASENT0903799), and *Pseudoneoponerabispinosa* (Smith). Also, some species of the *Loboponeravigilans* group (CASENT0003111), *Plectroctena*minor group (CASENT0915285), and *P.mandibularis* group (CASENT0102947) present the lateral margin of the propodeal declivity with a lamella that is dorsally toothed; in other species of the *L.vigilans* group, the lateral margin of the propodeal declivity is toothed dorsally, but the lamella is absent (CASENT0003098; see Bolton and Brown 2002).

22. In general, the shape of the propodeal spiracle is constant within genus in Ponerinae. A slit-shaped spiracle (i.e., external atrial opening > 2 × longer than wide; as in Keller 2011, character 65) is present in *Asphinctopone*, *Austroponera*, *Bothroponera*, *Buniapone*, *Corrieopone*, *Diacamma*, *Dinoponera*, *Ectomomyrmex*, *Euponera*, *Feroponera*, *Fisheropone*, *Hagensia*, *Harpegnathos*, *Megaponera*, *Odontoponera*, *Ophthalmopone*, *Pachycondyla*, *Paltothyreus*, *Phrynoponera*, *Pseudoneoponera*, and *Streblognathus*. A round to oval spiracle occurs in *Belonopelta*, *Boloponera*, *Dolioponera*, *Emeryopone*, *Hypoponera*, *Iroponera*, *Loboponera*, *Mayaponera*, *Myopias*, *Odontomachus*, *Plectroctena*, *Ponera*, *Promyopias*, *Psalidomyrmex*, *Rasopone*, *Simopelta*, and *Thaumatomyrmex*. The propodeal spiracle is round to oval in most *Brachyponera* species but varies from oval to slit-shaped in *B.atrata* and *B.sennaarensis*.

24. We classified the metacoxal cavities as closed or open by integrating the definition given by Keller (2011: character 69) with that in Fisher and Bolton (2016; see also Bolton 2003: character 41). If closed, the medial surface of the metacoxal acetabulum does not have a fenestra, and thus, the metacoxal cavity is not connected internally with the propodeal foramen; the annulus externally encircling the cavity is fused. If open, the internal medial surface of the cavity is fenestrate and connects with the propodeal foramen, and the annulus is unfused. In this case, the annulus may encircle the cavity with its free ends overlapping next to the propodeal foramen; or there may be a gap in the annulus.

An unfused cuticular annulus tightly encircles the metacoxal cavities in most Ponerinae we dissected. This condition occurs in *Phrynoponerapulchella* specimens from Kenya (CASENT0178203, CASENT0178204, CASENT0217125), which is in accord with Bolton and Fisher (2008a). However, a specimen from Tanzania possesses an annular gap in both metacoxal cavities (Fig. 46H); we are uncertain if those were natural or dissection artifacts, as unfortunately, only one specimen from that population was available to us. Metacoxal cavities also present an annular gap in *Phrynoponeratransversa* and *Platythyreapunctata*. On the other hand, the annulus is fused and uninterrupted in *Harpegnathossaltator*, *Platythyreacribrinodis* (Fig. 46F; contrary to Fisher and Bolton 2016), and *Myopiasdarioi* (Fig. 46G).

25. The calcar of strigil presents a basoventral lamella in most Ponerinae evaluated (as in Keller 2011, character 74). For clarification, we consider the calcar to present a small lamella in *Loboponeraobeliscata* (Fig. 46I) and that it is entirely pectinate in *Platythyreapunctata* and *P.turneri* (specimens ANTWEB1008574 and ANTWEB1008575, respectively), which is contrary to Keller (2011). The lamella is also absent in *Boloponera*, *Brachyponerasennaarensis*, *Diacammaceylonense*, *Dolioponerafustigera*, *Emeryoponebuttelreepeni*, *Harpegnathossaltator*, *Hypoponerapunctatissima*, *Leptogenysixta*, *L.peruana*, *L.pucuna*, *L.sonora*, *L.wheeleri*, *Loboponeravigilans*, *Mesoponeraambigua*, *M.elisaerotundata*, *Myopiasdarioi*, *Platythyreacribrinodis*, *Poneraalpha*, *P.pennsylvanica*, *Promyopiassilvestrii*, *Simopeltaoculata*, *S.transversa*, *Thaumatomyrmexfraxini*, and *T.zeteki*.

27. A row of stout, spine-like setae occurs along the posterior face of probasitarsal notch, parallel to the comb of strigil, in most Ponerinae taxa examined. We adopted the definition of “row” analogous to that of a line, whose existence requires at least two points in space. Thus, the row is present if two or more spine-like setae are aligned longitudinally along the posterior face of the probasitarsal notch; less than two setae make the row absent. Among material examined, the row is absent on the posterior face of the probasitarsal notch in *Dolioponerafustigera*, *Leptogenys*, *Myopiasdarioi*, and *Thaumatomyrmexfraxini*.

28. Most Ponerinae taxa present two mesotibial spurs. Among taxa examined, the anterior spur is simple, and the posterior spur is serrate in *Brachyponerachinensis*, *B.croceicornis*, *B.lutea*, *B.luteipes*, *B.obscurans*, *Dinoponeralongipes*, *D.lucida*, *Hagensiahavilandimarleyi*, *Harpegnathossaltator*, *Leptogenysperuana*, *L.pucuna*, and *Ophthalmoponeberthoudi*.

**Figure 47.**
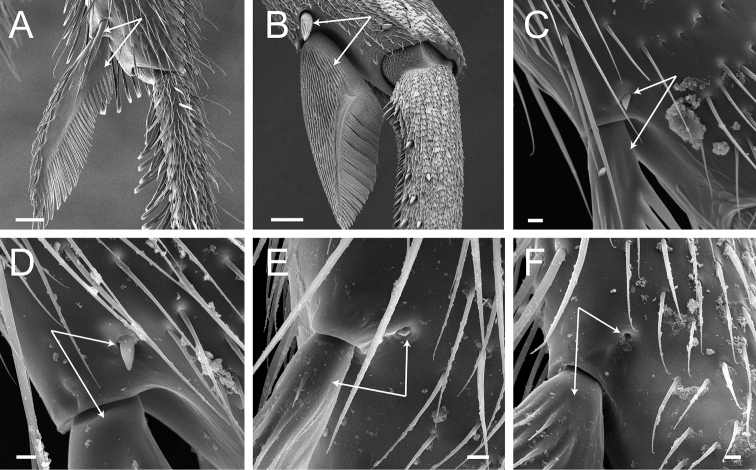
**A** apex of the metatibia and basal region of the metabasitarsus of *Cryptoponegilva* in anterior view; worker (ANTWEB1008514); arrows highlight the metatibial spurs **B** apex of the metatibia and basal region of the metabasitarsus of *Belonopeltadeletrix* in anterior view; worker (ANTWEB1008507); arrows highlight the metatibial spurs **C** ventroapical portion of the mesotibia of *Asphinctoponesilvestrii* in anteroventral view; worker (CASENT0824505); arrows highlight the minute anterior and much larger posterior spurs **D** ventroapical portion of the metatibia of *Asphinctoponesilvestrii* in anteroventral view (CASENT0824505); arrows highlight the minute anterior and much larger posterior spurs **E** ventroapical portion of the mesotibia of *Fisheroponeambigua* in ventral view; worker (CASENT0906216); arrows highlight the vestigial anterior and well-developed posterior spurs **F** ventroapical portion of the metatibia of *Fisheroponeambigua* in oblique anteroventral view (CASENT0906216); arrows highlight a fovea at the place the anterior spur would be located and the large posterior spur. Images by RA Keller (**A, B**) and FA Esteves (**C–F**); available at AntWeb.org. Scale bars: 0.03 mm (**A, B**); 0.004 mm (**C–F**).

For the record, only one mesotibial spur is visible under a stereomicroscope in *Asphinctoponesilvestrii* and *Fisheroponeambigua*; however, SEM images reveal that a vestigial anterior spur is present in both taxa (Fig. 47C, E); *A.differens* also seems to bear a minute anterior spur on the mesotibia.

29. The metatibial gland has been confirmed in *Bothroponeratesseronoda* (Emery), several species of *Diacamma* Mayr, *Harpegnathossaltator*, *Neoponeracrenata*, *N.marginata* (Roger), *Paltothyreustarsatus*, *Pseudoneoponerarufipes* (Jerdon), and *P.tridentata* (see Hölldobler et al. 1996; Billen 2009). The gland is associated with a cuticular pore plate, which may be covered by a brush of stouter, distinctly-shaped setae (as in *Diacamma* and *Paltothyreustarsatus*), or it may be an oblong, glabrous, distinctly colored, smooth, or distinctly sculptured cuticular patch (Hölldobler et al. 1996). Here, we list taxa that present the latter condition, which is what is seen in *Corrieopone*. Among the taxa examined, the cuticular patch is present on the apicoposterior face of the metatibia in *Bothroponeracrassa*, *B.silvestrii*, *Brachyponeracroceicornis*, *B.lutea*, *B.luteipes*, *B.obscurans*, *B.sennaarensis*, *Cryptoponehartwigi*, *Ectomomyrmexjavanus*, *Emeryoponebuttelreepeni*, *Euponerasikorae*, *E.sjostedti*, *Feroponeraferox*, *Hagensiahavilandimarleyi*, *Mesoponeracaffraria*, *Pseudoneoponeraporcata*, and *P.tridentata*.

30. Most Ponerinae taxa present two metatibial spurs. Here, all specimens examined present a pectinate posterior spur, and in the majority, the anterior spur is simple, as in *Corrieopone* (see Suppl. material 3: Table S3).

For the record, Schmidt and Shattuck (2014: 185) and Fernandes and Delabie (2019: 411) stated that *Cryptopone* species present one metatibial spur (excluding *C.guianensis*, and in the case of the latter authors, also *C.pauli*). Those assertions are puzzling, as several *Cryptopone* species have two spurs on the metatibia [e.g., *C.arabica* Collingwood & Agosti, *C.gilva*, *C.holmgreni* (Wheeler), *C.ochracea* Mayr; see Fig. 47A; Kempf 1961b; Bernard 1967; Collingwood and Agosti 1996]. *Belonopeltadeletrix* has two spurs on the metatibia (Fig. 47B), as correctly stated by Baroni Urbani (1975), but contrary to Schmidt and Shattuck (2014). *Asphinctoponesilvestrii* presents a minute additional metatibial spur (Fig. 47D), anterior to the much larger pectinate spur; *A.differens* seems to present the same. The metatibia of *Fisheroponeambigua*, which bears a single spur, long and pectinate, also presents a fovea where the anterior spur would be located (Fig. 47F).

31. Taxa without stout, spine-like setae on the dorsal face of the mid- and hindlegs are *Anochetusangolensis*, *A.emarginatus*, *Asphinctoponedifferens*, *As.silvestrii*, *Belonopeltadeletrix*, *Boloponeraikemkha*, *B.vicans*, *Bothroponerasilvestrii*, *Brachyponerachinensis*, *B.croceicornis*, *B.luteipes*, *B.obscurans*, *B.sennaarensis*, *Diacammaceylonense*, *Dolioponerafustigera*, *Emeryoponebuttelreepeni*, *Euponerasikorae*, *E.sjostedti*, *Hagensiahavilandimarleyi*, *Harpegnathossaltator*, *Iroponeraodax*, *Leptogenysixta*, *L.peruana*, *L.pucuna*, *L.sonora*, *L.wheeleri*, *Loboponeraobeliscata*, *L.vigilans*, *Mayaponeraconicula*, *Myopiasdarioi*, *M.maligna*, *Neoponerabugabensis*, *N.carinulata*, *N.cavinodis*, *N.crenata*, *N.dismarginata*, *N.fiebrigi* cf., *N.fisheri*, *N.foetida*, *N.globularia*, *N.insignis*, *N.inversa*, *N.luteola*, *N.moesta*, *N.obscuricornis*, *N.striadinodis*, *N.unidentata*, *N.villosa*, *Odontomachusbauri*, *Odontoponeratransversa*, *Ophthalmoponeberthoudi*, *Platythyreacribrinodis*, *P.punctata*, *P.turneri*, *Simopeltaoculata*, *S.transversa*, *Thaumatomyrmexfraxini*, and *T.zeteki*.

**Figure 48.**
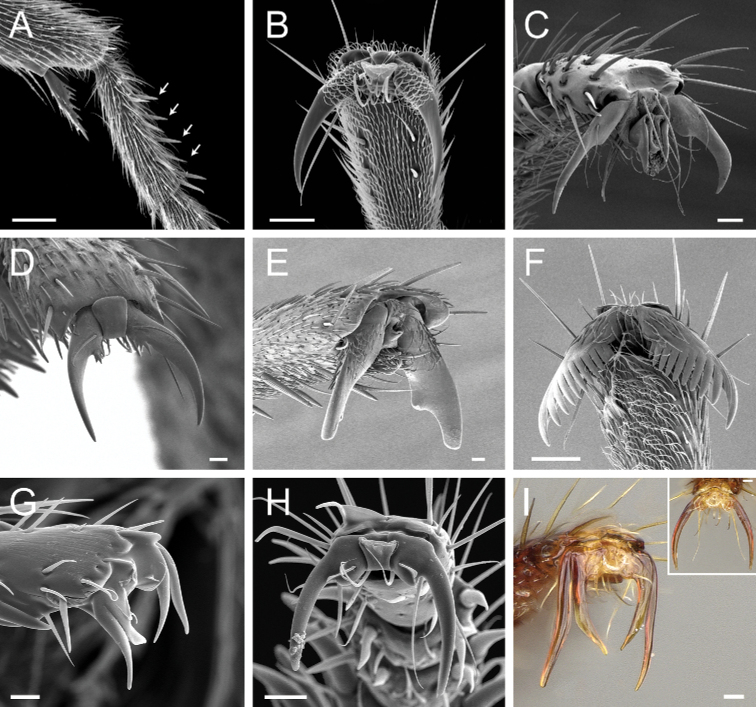
**A** mesobasitarsus of *Fisheroponeambigua* in anterior view; worker (CASENT0906209); arrows indicate the stout, spine-like setae on the dorsal face of the tarsomere **B** pretarsus of *Mayaponeraconstricta* in ventral view; worker (CASENT0260254) **C** pretarsus of *Simopeltatransversa* in oblique apicolateral view; worker (ANTWEB1008589) **D** pretarsus of *Bothroponerapachyderma* in oblique dorsal view; worker (ANTWEB1008567) **E** pretarsus of *Hagensiahavilandimarleyi* in apicolateral view; worker (ANTWEB1008566) **F** pretarsus of *Leptogenyswheeleri* in ventral view; worker (ANTWEB1008541) **G** propretarsus of *Promyopiassilvestrii* in dorsolateral view; worker (CASENT00178751) **H** metapretarsus of *Promyopiassilvestrii* in apical view (CASENT00178751) **I** propretarsus of *Buniaponeamblyops* in apicolateral view; worker (CASENT0384973); insert: metapretarsus of *Buniaponeamblyops* in dorsoapical view (CASENT0384973). Images by FA Esteves (**A, B, G–I**) and RA Keller (**C–F**); available at AntWeb.org. Scale bars: 0.04 mm (**A, B, F**); 0.02 mm (**C–E, G–I**).

Contrary to Fisher and Bolton (2016), *Fisheroponeambigua* presents stout, spine-like setae on the dorsal face of the mesobasitarsus (Fig. 48A).

33. We categorized the shape of pro-, meso-, and metapretarsal claws according to the presence, location, and number of acute projections on their inner margins (modified from Keller 2011, character 82). A simple pretarsal claw is devoid of prominences (Fig. 48B) or presents a blunt basal angle or rounded swell (Fig. 48C), and is found in the following taxa: *Anochetusangolensis, A.emarginatus*, *Asphinctoponedifferens*, *A.silvestrii*, *Austroponeracastanea*, *Belonopeltadeletrix*, *Boloponeraikemkha*, *B.vicans*, *Bothroponeracrassa*, *B.silvestrii*, *Brachyponerachinensis*, *B.croceicornis*, *B.lutea*, *B.luteipes*, *B.obscurans*, *B.sennaarensis*, *Centromyrmexbrachycola*, *C.decessor*, *C.ereptor*, *C.raptor*, *Cryptoponegilva*, *C.guianensis*, *C.hartwigi*, *Diacammaceylonense*, *Dolioponerafustigera*, *Ectomomyrmexjavanus*, *Emeryoponebuttelreepeni*, *Euponerabrunoi*, *E.sikorae*, *E.sjostedti*, *Feroponeraferox*, *Fisheroponeambigua*, *Hypoponerapunctatissima*, *Iroponeraodax*, *Loboponeraobeliscata*, *L.vigilans*, *Mayaponerabecculata*, *M.cernua*, *M.conicula*, *M.constricta*, *Mesoponeraambigua*, *M.australis*, *M.caffraria*, *M.elisaerotundata*, *M.melanariamacra*, *M.papuana*, *M.rubra*, *M.subiridescens*, *Myopiasdarioi*, *M.maligna*, *Neoponeraaenescens*, *N.apicalis*, *N.bugabensis*, *N.cavinodis*, *N.crenata*, *N.dismarginata*, *N.eleonorae*, *N.fiebrigi* cf., *N.fisheri*, *N.foetida*, *N.globularia*, *N.insignis*, *N.inversa*, *N.laevigata*, *N.luteola*, *N.obscuricornis*, *N.schoedli*, *N.striadinodis*, *N.unidentata*, *N.verenae*, *N.villosa*, *Odontomachusbauri*, *Odontoponeratransversa*, *Pachycondylalattkei*, *Parvaponeradarwiniimadecassa*, *Plectroctenastrigosa*, *Poneraalpha*, *P.pennsylvanica*, *Psalidomyrmexprocerus*, *Pseudoneoponeraporcata*, *Pseudoponeragilberti*, *P.stigma*, *Rasoponecostaricensis*, *R.cryptergates*, *R.cubitalis*, *R.guatemalensis*, *R.panamensis*, *R.pluviselva*, *R.politognatha*, *Simopeltaoculata*, *S.transversa*, and *Streblognathuspeetersi*.

A claw with a basal tooth bears an acute prominence on the basal third of its inner margin (Fig. 12B). The basal tooth overhangs the outline of the inner margin of the claw and may bear several small, acute projections (Fig. 48D). This type of claw occurs in *Bothroponeracariosa*, *B.pachyderma*, *B.talpa*, *Mayaponeraarhuaca*, *M.pergandei*, *Megaponeraanalis*, *Neoponeracarinulata*, *N.commutata*, *N.moesta*, *Ophthalmoponeberthoudi*, *Pachycondylacrassinoda*, *P.harpax*, *P.impressa*, *P.lenis*, *P.procidua*, *P.striata*, *Phrynoponerapulchella*, *P.transversa*, and *Pseudoneoponeratridentata*. A claw with a preapical tooth has an acute projection rising from the apical two-thirds of its inner margin (Fig. 48E). It is present in *Dinoponeralongipes*, *D.lucida*, *Hagensiahavilandimarleyi*, *Harpegnathossaltator*, *Paltothyreustarsatus*, *Platythyreacribrinodis*, *P.punctata*, *P.turneri*, *Thaumatomyrmexfraxini*, and *T.zeteki*. Pectinate pretarsal claws are shaped like a comb and are unique to *Leptogenys* among Ponerinae (Fig. 48F).

Finally, we found shape variations among the claws of the fore-, mid-, and hindlegs only in *Buniaponeamblyops* and *Promyopiassilvestrii*. These taxa present a propretarsal claw with a long basal tooth, while their meso- and metapretarsi claws are simple (Fig. 48G–I). This condition contradicts Bolton and Fisher (2008b) and Fisher and Bolton (2016), who stated the claws are simple in *P.silvestrii*.

34. The arolium was absent or reduced to a membranous cuticular flap between the pretarsal claws of most taxa examined (Figs 12B, 48D–I). Exceptions were *Belonopeltadeletrix*, *Diacammaceylonense, Harpegnathossaltator*, *Iroponeraodax*, *Mayaponera*, *Neoponera*, *Parvaponeradarwiniimadecassa*, *Platythyreacribrinodis*, *P.punctata*, *P.turneri*, *Rasoponeguatemalensis*, *Simopeltaoculata*, *S.transversa* (Fig. 48C), *Thaumatomyrmexfraxini*, and *T.zeteki*.

Contrary to Schmidt and Shattuck (2014), we consider that *Mayaponeraconstricta* has indistinct arolia like all its congeners (Fig. 48B). In this species, the arolium is reduced to a cuticular flap, although more developed than what we classified as indistinct in other taxa. However, this feature was not noticeable under our stereo microscope.

35. The petiolar node of *Corrieopone* lacks a spine-like or any other acute projection and is narrow in profile, with its anterior and posterior surfaces tapering to an insignificant dorsal surface.

Ponerine taxa with an unarmed scale-like petiole are: several *Anochetus* (see CASENT0915154); *Asphinctopone* (CASENT0915481); *Austroponera* (FOCOL0965); *Brachyponera* (CASENT0915660); *Buniapone* (CASENT0903944); some *Cryptopone* (ANTWEB1008000); some *Ectomomyrmex* (CASENT0907270); *Euponerafossigera* species group [viz.: *E.brunoi*, *E.fossigera* Mayr (SAM-HYM-C002649B), *E.malayana* (Wheeler), *E.sharpi* Forel, *E.wroughtonii* Forel, *E.wroughtoniicrudelis* Forel, and probably also *E.sakishimensis* (Terayama)]; *Fisheropone* (CASENT0906215); *Hagensia* (CASENT0256487); several *Hypoponera* (CASENT0281911); some *Leptogenys* (CASENT0902609); *Mayaponera* (USNMENT00442104); *Mesoponera* (CASENT0249169); some *Neoponera* (ANTWEB1014009); very few *Odontomachus* (CASENT0281868); very few *Pachycondyla* (UFV-LABECOL-000002); some *Parvaponera* (CASENT0915276); some *Ponera* (CASENT0235336); and *Pseudoponera* [CASENT0902509; except *P.pachynoda* (Clark), ANTWEB1008183].

**Figure 49.**
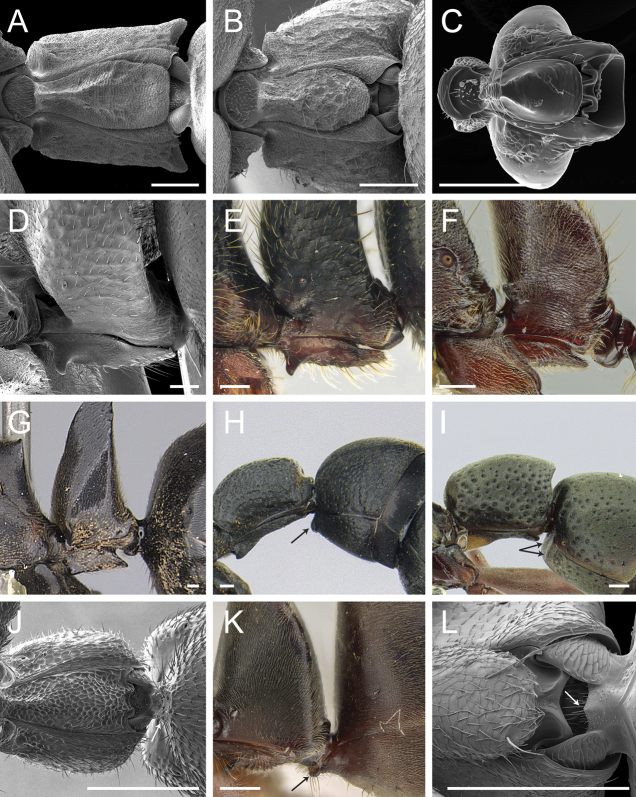
**A** petiole of *Platythyreapunctata* in ventral view; worker (ANTWEB1008574) **B** petiole of *Platythyreaturneri* in ventral view; worker (ANTWEB1008575) **C** petiole of *Asphinctoponesilvestrii* in ventral view; worker (CASENT0824505) **D** petiole of *Phrynoponerapulchella* in profile; worker (ANTWEB1008573) **E** petiole of *Phrynoponeragabonensis* in profile; worker (CASENT0250060) **F** petiole of *Rasopone* jtl030 in profile; worker (CASENT0633075) **G** petiole of *Streblognathuspeetersi* in profile; worker (CASENT0258947) **H** petiole and abdominal segment IV of *Platythyrealamellosa* in profile; worker (CASENT0252018); arrow indicates the prora **I** petiole and abdominal segment IV of *Platythyreapillosula* in profile; worker (CASENT0260481); arrows indicate the prora **J** petiole and anterior portion of the abdominal segment IV of *Iroponeraodax* in ventral view; worker (ANTWEB1008537); arrow indicates the prora **K** petiole and anterior portion of the abdominal segment IV of *Mayaponeraconstricta* in profile; worker (CASENT0643469); arrow indicates the prora **L** posterior portion of the petiole and the helcium of *Brachyponeracroceicornis* in ventral view; worker (ANTWEB1008564); arrow indicates the minute prora projected from the area in between the ventral margins of the helcium tergite. Images by RA Keller (**A, B, D, J, L**), FA Esteves (**C,K**), B Reynolds (**E, H**), JT Longino (**F**), M Esposito (**G**), and W Ericson (**I**); available at AntWeb.org. Scale bars: 0.2 mm.

36. The strigation on the posteroventral portion of the petiolar tergite of *Corrieopone* resembles that of *Asphinctopone* (CASENT0178221).

39. The spatulate projection rises from the posterior portion of the petiolar sternite and extends posteriad, overlapping either partially or entirely the remaining sternite. This definition departs from Keller (2011, character 111) in not requiring (a) the projection to extend beyond the posterior margin of the petiolar sternite and (b) close proximity between projection and remaining sternite. The reason is the character varies continuously, which invalidates both requirements. The variation comprises projections that are long and conceal the helcial sternite almost completely to partially (as in *Phrynoponerapulchella*, specimen ANTWEB1008573, and *Platythyreapunctata*, respectively; Fig. 49A); those that reach or almost reach the posterior margin of the petiolar sternite (as in *Platythyreaturneri*, Fig. 49B); and those even shorter (as in *Asphinctoponesilvestrii*; Fig. 49C). In addition, projections may tightly envelop the remaining sternite (as in *Phrynoponerapulchella* and *Platythyreapunctata*, ANTWEB1008574; Fig. 49D); or a slight gap may be present [as in *Platythyreaturneri*, specimen ANTWEB1008575, and *Phrynoponeragabonensis* (André); Fig. 49E]; or the gap may be slightly broader (as in *Rasopone* jtl030; Fig. 49F) to much broader (as in *Streblognathuspeetersi*; Fig. 49G).

Thus, according to our definition, the spatulate projection of the petiolar sternite is present in *Austroponera*, *Asphinctopone*, *Brachyponera*, *Megaponeraanalis*, *Ophthalmopone*, *Phrynoponera*, *Platythyrea*, *Rasopone*, and Streblognathus; see also Fisher and Bolton (2016) for the description of the sternite in some of these taxa. The petiolar sternite in *Belonopeltadeletrix*, immediately anterior to its posterior margin, is projected ventrad and slightly posteriad; the same seems to happen in *Thaumatomyrmexfraxini*. According to the species redescription given by Mackay and Mackay (2010), *Neoponeramagnifica* may also present the character.

40. Like *Corrieopone*, most ponerine genera present an infra-axial helcium (i.e., positioned ventrad the midheight of the anterior face of abdominal segment III; see Keller 2011, character 114). A few taxa examined present an axial helcium (i.e., positioned at midheight of the anterior face of abdominal segment III): *Boloponera*, *Buniapone*, *Centromyrmex*, *Cryptopone* (except *C.guianensis*), *Dolioponera*, *Feroponera*, *Iroponera*, *Platythyrea*, *and Promyopias*.

41. As far as we know, the prora is present and well-developed in most Ponerinae. It is usually indistinct in *Platythyrea*, but contrary to Fisher and Bolton (2016), it is well-developed in some species [as in *P.lamellosa* (Roger); Fig. 49H], and weakly projected but still visible in others [as in *P.pilosula* (Smith); Fig. 49I]. According to Schmidt and Shattuck (2014), the prora is absent in *Iroponera*, but SEM images of *I.odax* reveal that it is present, albeit weakly projected (Fig. 49J). According to Fisher and Bolton (2016), the trait is absent in *Dolioponera*, which agrees with what we saw in most specimens. However, SEM images of specimen ANTWEB1008521 show a weak projection on the anterior face of abdominal sternite III; we are unsure whether that detail is natural or an image artifact.

We consider the prora present in *Mayaponera* and *Rasopone*, in disagreement with Longino and Branstetter (2020). The trait, although small, is distinct in the profile view of *M.becculata*, *M.conicula*, *M.constricta*, and *R.panamensis*. In the first two species and *R.panamensis*, the prora rises from the anterior portion of the abdominal poststernite III (see specimens CASENT0249130, CASENT0644252). In *M.constricta*, it projects from the area in between the ventral margins of the helcial tergite arch and the anterior portion of the abdominal poststernite III (Fig. 49K) and resembles the condition seen in *Dinoponera*, *Pachycondyla*, and *Streblognathus* (ANTWEB1014000, CASENT0249148, and Fig. 49G, respectively). In *M.arhuaca*, *M.cernua*, *M.pergandei*, and other *Rasopone* species, the prora is indistinct in the profile view of undissected specimens, for it is a minute prominence located in the area between the ventral margins of the helcial tergite (as in *Brachyponera*, Fig. 49L), and may be fused entirely with it (as described for *Phrynoponera* by Bolton and Fisher 2008a).

43. We considered the girdling constriction present if the surface between pre- and postsclerites of abdominal segment IV was interrupted by a shallow or deep impression on the integument. A line, if present, only constituted a constriction if the integument was depressed.

Among taxa examined, the constriction is absent in *Asphinctopone*, *Brachyponerasennaarensis* (weakly impressed in other species), *Corrieopone*, *Mesoponera* (except *M.caffraria*), *Odontomachus*, *Odontoponera*, *Simopelta*, *Streblognathus*, and *Thaumatomyrmexfraxini*.

44. The stridulitrum is consistently present on abdominal pretergite IV in *Austroponera*, *Belonopelta*, *Dinoponera*, *Harpegnathos*, *Megaponera*, *Neoponera*, *Odontoponera*, *Ophthalmopone*, *Streblognathus*, and *Thaumatomyrmex*. The trait’s occurrence is variable in *Anochetus*, *Bothroponera* (present in members of the *sulcata* group), *Brachyponera*, *Hypoponera*, *Mayaponera* (only present in *M.constricta*), *Mesoponera*, *Myopias*, *Odontomachus*, *Phrynoponera* (only present in *P.pulchella*), and *Ponera* (Suppl. material 3: Table S3; see also Markl 1973; Schmidt and Shattuck 2014; Fisher and Bolton 2016). Schmidt and Shattuck (2014) state that it is universally present in *Leptogenys* and *Platythyrea*. However, Markl (1973), after a comprehensive taxa examination, affirmed that the occurrence of the trait is variable among species in both genera.

**Figure 50.**
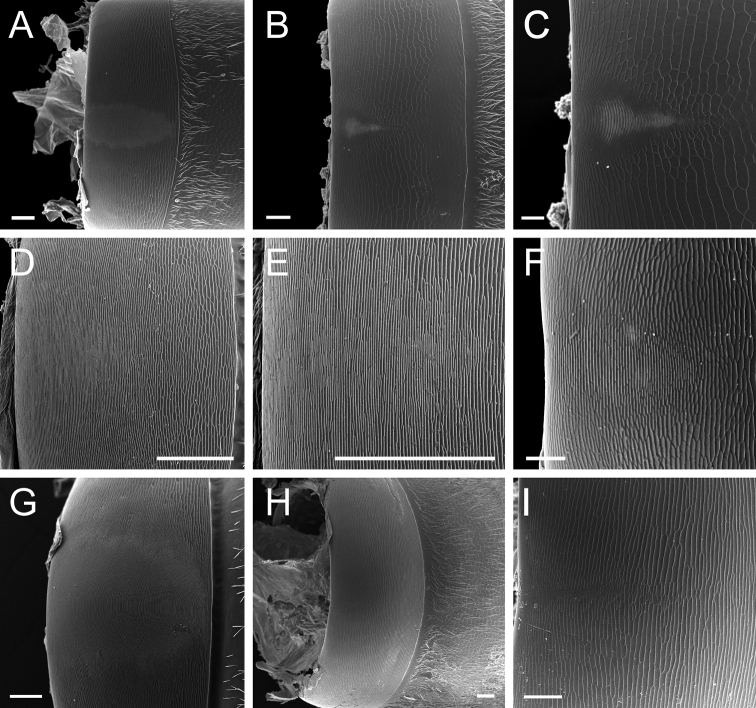
**A** abdominal pretergite IV of *Brachyponeraobscurans* in dorsal view; worker (CASENT0059638) **B** abdominal pretergite IV of *Mesoponerasubiridescens* in dorsal view; worker (CASENT0906219) **C** stridulitrum of *Mesoponerasubiridescens* in dorsal view (CASENT0906219) **D** medial area of the abdominal pretergite IV of *Euponerasikorae*; worker (CASENT0497109) **E** close-up of the medial area of the abdominal pretergite IV of *Euponerasikorae*; worker (CASENT0134370) **F** medial area of the abdominal pretergite IV of *Mesoponeracaffraria*; worker (CASENT0408614) **G** medial area of the abdominal pretergite IV of *Mayaponerapergandei*; male (CASENT0317474) **H** abdominal pretergite IV of *Mayaponerapergandei* in dorsal view; worker (CASENT0845437) **I** close-up of the medial area of the abdominal pretergite IV of *Mayaponerapergandei*; worker (CASENT0845437). Images by FA Esteves; available at AntWeb.org. Scale bars: 0.06 mm (**A, F, G, I**); 0.09 mm (**B**); 0.04 mm (**C**); 0.2 mm (**D, E**); 0.08 mm (**H**).

Contrary to Fisher and Bolton (2016), the stridulitrum is present in *Brachyponeraobscurans* (Fig. 50A) and at least two Afrotropical and Malagasy species of *Mesoponera* (Fig. 50B, C). For the record, it is absent in *Boloponeraikemkha*. In addition, the midline of the fourth abdominal pretergite is strigulate and dissimilar to the surrounding sculpture in *Euponerasikorae* (Fig. 50D, E) and *Mesoponeracaffraria* (Fig. 50F); the ridges are set farther apart and form a narrower area in the latter species. However, whether they have a stridulatory function in those species is unclear. Finally, the stridulitrum is present in males of *Mayaponerapergandei* (Fig. 50G; contrary to Mackay and Mackay 2010) but absent in the worker caste (Fig. 50H, I). Assuming that W. P. Mackay correctly determined the two males we examined, this intercaste variation is interesting because (1) it has not been observed in ants (see Markl 1973), and (2) it suggests that this trait has a reproductive function in *M.pergandei*.

47. The hypopygium (abdominal sternite VII) is armed with spine-like setae that flank the sting in some Ponerinae. According to previous studies, the setae are present in *Dinoponera*, *Ophthalmopone*, *Pachycondyla*, *Paltothyreus*, some *Leptogenys*, and few *Ponera* (Schmidt and Shattuck 2014; Fisher and Bolton 2016). However, in *Pachycondyla*, we found that the hypopygial setae may be spine-like or aristate (Figs 10C–F, 12C, D; see details in the preceding subsection “Transfers between *Neoponera* to *Pachycondyla*”).

**Figure 51.**
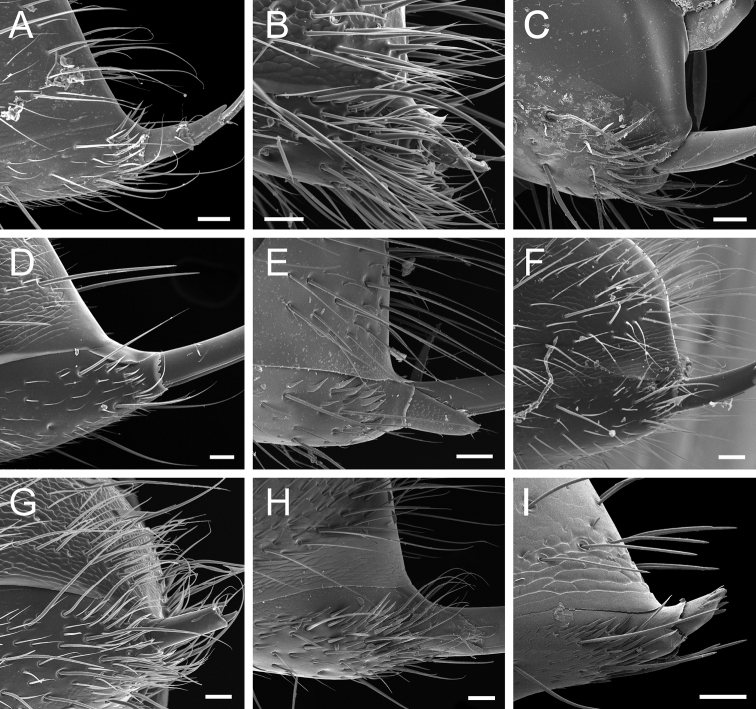
Posterior portion of abdominal segment IV in profile (only the hypopygium is seen in image C); in each image, a sample of stout, spine-like seta (or microtrichia, in image I) is highlighted in orange **A***Brachyponerachinensis*; worker (CASENT0104738) **B***Buniaponeamblyops*; worker (CASENT0384786) **C***Mayaponerabecculata*; worker (CASENT0428720) **D***Mesoponeramelanariamacra*; worker (CASENT0159302) **E***Myopiasdarioi*; worker (CASENT0810080) **F***Parvaponeradarwiniimadecassa*; worker (CASENT0389498) **G***Promyopiassilvestrii*; worker (CASENT00178751) **H***Rasoponepanamensis*; worker (CASENT0644252) **I***Thaumatomyrmexfraxini*; worker (ANTWEB1008597). Images by FA Esteves (**A–H**) and RA Keller (**I**); available at AntWeb.org. Scale bars: 0.04 mm.

Hypopygial spine-like setae occur in several *Brachyponera* (Fig. 51A), *Buniaponeamblyops* (Fig. 51B), *Mayaponerabecculata* (Fig. 51C), some *Mesoponera* (Fig. 51D), *Myopiasdarioi* (Fig. 51E), *M.maligna*, some *Neoponera* (Fig. 10A, B; see subsection “Transfers between *Neoponera* to *Pachycondyla*”), *Parvaponeradarwiniimadecassa* (Fig. 51F), *Promyopiassilvestrii* (Fig. 51G), and *Rasoponepanamensis* (Fig. 51H; see also Suppl. material 3: Table S3). In *Thaumatomyrmexfraxini* and *T.zeteki*, the hypopygium vestiture is composed of minute spine-like microtrichia (Fig. 51I).

### ﻿Comparison with similar genera

*Corrieopone* gen. nov. is morphologically distinctive and unlikely to be confused with any other Ponerinae genus. To our knowledge, no other Ponerinae has a clypeal medial area that projects anteriorly as a broad, truncated prominence that overlaps the mandibles dorsally and presents a broad anteroventral face, which is subrectangular in ventro-anterior view (Figs 37B, 38A–E, 39A–D). In addition, no other ant presents the ventral face of the hypopygium with a longitudinal concavity that posteriorly bears a longitudinal carina and stout, hook-shaped setae.

**Figure 52.**
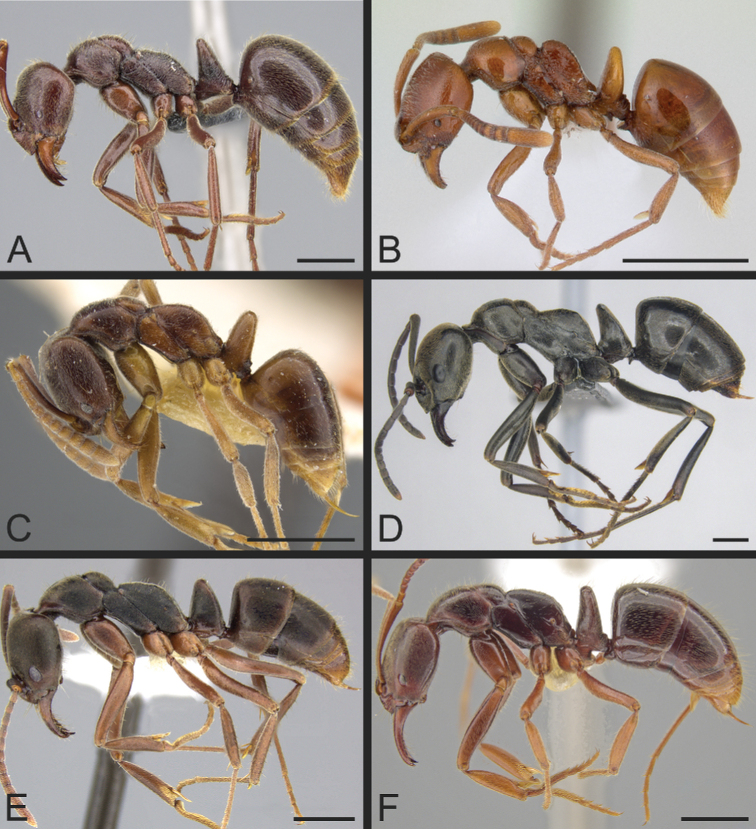
**A***Corrieoponenouragues*; holotype, worker (CASENT0830464) **B***Asphinctoponesilvestrii*; worker (CASENT0406793) **C***Brachyponeraarcuata*; syntype, worker (CASENT0916828) **D***Hagensiaperingueyi*; worker (CASENT0256487) **E***Mayaponeraconstricta*; worker (CASENT0249137) **F**Mesoponeracf.ambigua; worker (CASENT0629612). Images by W Lee (**A**), A Nobile (**B**), K Martynova (**C**), B Reynolds (**D**), R Perry (**E**), and JT Longino (**F**); available at AntWeb.org. Scale bars: 1 mm.

Despite these features, workers of *Corrieopone* superficially resemble *Asphinctopone*, *Brachyponera*, some *Hagensia*, some *Mayaponera*, and most *Mesoponera* species, because they share the following characters: the eyes are located on the anterior part of the head; the mesonotum is dome-shaped; the metanotal sulcus is deeply impressed; the notopleural suture is conspicuous; the metathoracic spiracles are concealed by a cuticular lobe; the dorsoposterior area of the propodeum lacks spines or tubercles; the petiole is unarmed and scale-like; the constriction between the presclerites and postsclerites of abdominal segment IV is shallowly impressed or absent (Fig. 52A–F).

However, *Corrieopone* differs from these genera in many characters (Tables 2, S3), including those of the mandibles (only in *Corrieopone* it is edentate), clypeus (only *Corrieopone* has the clypeal anterior projection shaped as described above), and hypopygium (only *Corrieopone* has a hypopygium as described above). In *Corrieopone*, (1) the mandible is devoid of a dorsolateral sulcus or basolateral or dorsal pits (the mandible presents a dorsal pit in *Hagensia*, a basolateral pit in *Brachyponera*, and a dorsolateral sulcus in *Asphinctopone* and *Mesoponerasubiridescens*); (2) the palpal formula is 4,4 (it is 3,3 in *Asphinctopone*, *Brachyponera*, and *Mesoponeraambigua*); (3) the mesopleuron is distinctively divided into anepisternum and katepisternum (it is undivided in *Mesoponera*, excluding *M.subiridescens*, and *Mayaponera*, excluding *M.conicula*); (4) the propodeal spiracle is slit-shaped (it is round to oval in *Brachyponera* save *B.sennaarensis*, *Mayaponera*, and *Mesoponera*, excluding *M.caffraria* and subspecies, *M.ingesta*, and *M.subiridescens*); (5) the apicoposterior area of the metatibia presents an apparent metatibial gland cuticular patch (it is absent in *Asphinctopone*, *Brachyponerachinensis*, *Mayaponera*, and *Mesoponera* save *M.caffraria*, and likely its subspecies, and *M.ingesta*); (6) meso- and metatibiae bear two well-developed spurs (*Asphinctopone* only has one well-developed spur on each tibia); (7) pretarsal claws are simple (the claws present a preapical tooth in *Hagensia*); (8) the posterior portion of the petiole sternite is not projected (it presents a spatulate projection that folds posteriad over the remaining sternite in *Asphinctopone* and *Brachyponera*); (9) the prora is conspicuous in profile (it is indistinct in profile of undissected specimens in *Brachyponera*, and some *Mayaponera* species); (10) the pretergite of abdominal segment IV presents a stridulitrum (the stridulitrum is absent in *Asphinctopone*, *Brachyponerasennaarensis*, *Hagensia*, *Mayaponera* save *M.constricta*, and *Mesoponeraambigua*); and (11) the posterolateral portion of the hypopygium lacks stout, spine-like setae (the setae are present in *Mayaponerabecculata*, and *Mesoponera* save species occurring in the afrotropics). Moreover, *Asphinctopone* has an oblong, smooth, bright, yellowish cuticular patch on the dorsal face of its subtriangular mandibles (Hawkes 2010); the cuticular patch is absent from the triangular mandibles of *Corrieopone*.

**Table 2.** Character matrix for ponerine genera that superficially resemble *Corrieopone*.

Genus	2	3.1	3.2	3.3	4	11	15	22
*Asphinctopone*	1	0	0	1	0	3,3	1	0
*Brachyponera*	1	1**^a^**	0	0	0	3,3	0,1	1**^f^**
*Corrieopone*	0	0	0	0	1	4,4	1	0
*Hagensia*	1	0	1	0	0	4,4	1	0
*Mayaponera*	1	0	0	0	0	4,4	0**^d^**	1
*Mesoponera*	1	0	0	0**^b^**	0	3,3; 4,3; 4,4**^c^**	0,1**^e^**	1**^g^**
**Genus**	**29**	**30**	**33**	**39**	**41**	**44**	**46**	**47**
*Asphinctopone*	0	1	0	1	1	0	0	0
*Brachyponera*	1**^h^**	2	0	1	2	0,1**^j^**	0	1
*Corrieopone*	1	2	0	0	1	1	1	0
*Hagensia*	1	2	2	0	1	0	0	0
*Mayaponera*	0	2	0,1	0	1,2	0**^k^**	0	0**^m^**
*Mesoponera*	0**^i^**	2	0	0	1	0,1**^l^**	0	1**^n^**

Character numbers correspond to characters used in genus diagnosis (see genus diagnosis and “Comments on worker characters” for character definition): **2** Mandibles, dentition: (0) edentate, (1) dentate. **3.1** Mandibles, basolateral pit: (0) absent; (1) present (as in Fig. 45A, B). **3.2** Mandibles, dorsal pit: (0) absent; (1) present (as in Fig. 45C). **3.3** Mandibles, dorsolateral sulcus: (0) absent; (1) present (as in Fig. 45D). **4** Clypeus, anteroventral face: (0) absent; (1) present (avf, Fig. 39A–D). **11** Palpal formula: number of maxillary palps, number of labial palps. **15** Mesopleuron, division into anepisternum and katepisternum: (0) absent, (1) present. **22** Propodeal spiracle, shape: (0) slit-shaped, (1) round to oval. **29** Metatibia, apparent metatibial gland cuticular patch: (0) absent; (1) present (as in Fig. 41E). **30** Metatibia, number of well-developed spurs (i.e., number of spurs seen under a stereomicroscope). **33** Pretarsal claws, shape: (0) simple (as in Fig. 48B, C); (1) basal tooth (as in Figs 12B, 48D); (2) pre-apical tooth (as in Fig. 48E). **39** Petiolar poststernite, posterior spatulate projection: (0) absent; (1) present (as in Fig. 49A–G). **41** Abdominal poststernite III, prora: (1) present and conspicuous; (2) present (Fig. 49L), but indistinct in the profile of undissected specimens. **44** Abdominal pretergite IV, stridulitrum: (0) absent, (1) present. **46** Abdominal sternite VII (hypopygium), ventral face: (0) convex or flat; (1) distinctively concave longitudinally (Fig. 44). **47** Abdominal sternite VII (hypopygium), stout, spine-like setae on posterolateral portion: (0) absent, (1) present. Superscripts: **^a^** Absent in species from Borneo, Bali, Krakatau, and Sumatra (Yamane 2007). **^b^** Present in *Mesoponerasubiridescens.***^c^** The palpal formula is 4,4 in all species in which the character was assessed save *Mesoponeraambigua*, whose formula is 3,3. According to Fisher and Bolton (2016), Afrotropical and Malagasy species may present palpal formula 3,3; 4,3; 4,4. **^d^** Present in *Mayaponeraconicula*. **^e^** Present in *Mesoponerasubiridescens* and very shallowly impressed in some *M.melanariamacra* specimens and Indomalayan and Australasian species. **^f^** Oval to slit-shaped in *Brachyponerasennaarensis* among species examined; slit-shaped in images of *B.sennaarensis* subspecies on AntWeb.org. According to Schmidt and Shattuck (2014: 79), the character is round in all other species. **^g^** Slit-shaped in *Mesoponeracaffraria* and subspecies, *M.ingesta*, and *M.subiridescens.***^h^** Among species examined (*N* = 6), the character is absent only in *Brachyponerachinensis*. It is visible in images of *B. arcuata, B.atrata*, and *B.sennaarensisruginota* on AntWeb.org. **^i^** Among species examined (*N* = 8), the character is present only in *Mesoponeracaffraria*; it is visible in images of specimens identified as *M.ingesta* on AntWeb.org. **^j^** Among species examined (*N* = 6), the stridulitrum is absent only in *Brachyponerasennaarensis*, but see Schmidt and Shattuck (2014: 79). **^k^** Present in *Mayaponeraconstricta*. **^l^** Among species examined (*N* = 8), the stridulitrum is only absent in *Mesoponeraambigua*. The character was not assessed in *M.elisaerotundata*; it is unclear in *M.caffraria*; but see Schmidt and Shattuck (2014: 109). **^m^** Among species examined (*N* = 8), the character is only present in *Brachyponerabecculata*. **^n^** Among material examined, the character is absent in the Afrotropical species.

Preliminary phylogenetic analysis based on molecular data inferred a sister relationship between *Corrieopone* and *Asphinctopone* (The Ants of the World Project, unpublished data); however, analysis of a more comprehensive dataset is still ongoing and upcoming results may challenge this hypothesis.

### ﻿Other castes and stages of development

**Figure 53.**
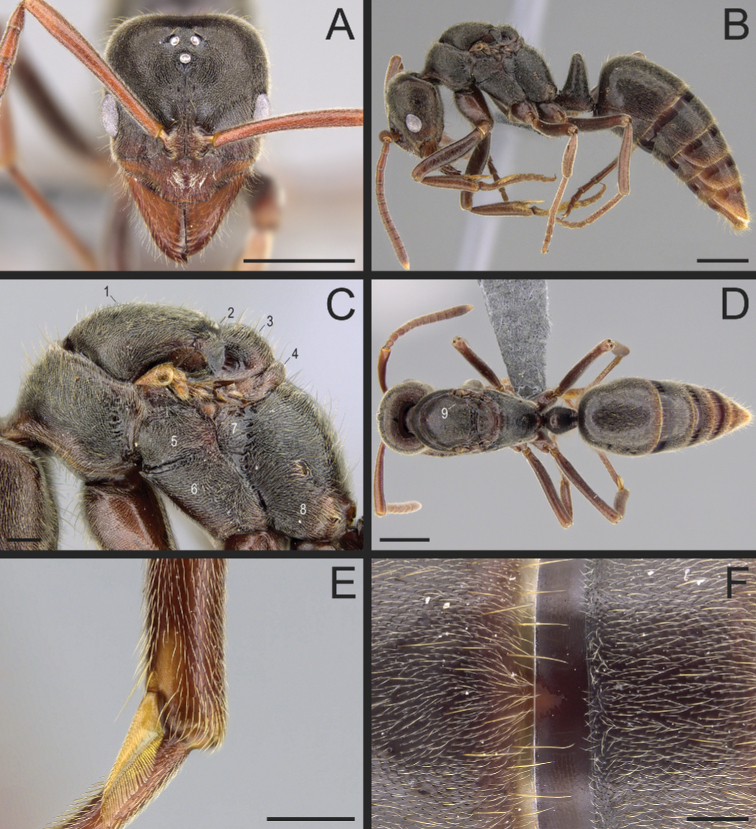
*Corrieoponenouragues*, paratype, dealated queen (CASENT0923157) **A** head in full-face view **B** body in profile **C** mesosoma in profile **D** body in dorsal view **E** metatibial apex and basal portion of the metabasitarsus in posterior view **F** medial area of abdominal segments III and IV in dorsal view. Images by M Esposito (**A, B, D, F**) and FA Esteves (**C, E**); available at AntWeb.org. Numbers: **1**, mesoscutum; **2**, indentation of the scutoscutellar sulcus; **3**, mesoscutellum; **4**, metanotum; **5**, anepisternum; **6**, katepisternum; **7**, upper section of the metapleuron; **8**, lower section of the metapleuron; **9**, parapsidal line. Scale bars: 1 mm (**A, B, D**); 0.2 mm (**C, E, F**).

***Queen*** (Fig. 53A–F): Winged. Similar to the worker caste but for the slightly greater body length (HL: 1.65; TL: 8.48), larger compound eyes, presence of ocelli, differences in the mesosoma due to the presence of wings, and darker color. Clypeus as described in the definition of the genus. Parapsidal lines present on the mesoscutum (Fig. 53D). In profile, mesoscutum bulging; higher than the pronotum; slightly higher than the mesoscutellum (Fig. 53C). In profile, scutoscutellar sulcus indenting the dorsal outline of the mesoscutum and mesoscutellar-axillar complex. Mesoscutellum dome-shaped in profile; higher than the metanotum and the propodeum (Fig. 53C). Mesopleuron in profile divided into anepisternum and katepisternum. In profile, metapleuron divided into upper and lower sections; upper metapleuron separated from propodeum by sulcus; lower metapleuron indistinct from propodeum (Fig. 53C). Propodeal spiracle slit-shaped. Legs as in the worker caste, including the presence of the metatibial gland cuticular patch on the apicoposterior face of the metatibia (Fig. 53E). Petiole and gaster shaped as in the worker caste (Fig. 53B). Prora present; lip-shaped. Stridulitrum small, as in workers (Fig. 53F). Hypopygium as described in the definition of the genus.

***Male***: Unknown.

***Larva***: Unknown.

**Etymology.** The genus name *Corrieopone* is feminine. It is a tribute to Dr. Corrie Saux Moreau, a myrmecologist, professor of Arthropod Biosystematics and Biodiversity at Cornell University, and director and curator of the Cornell University Insect Collection, in Ithaca, N.Y. A flagbearer for gender inclusion and diversity in our field, audacious (see Wilson 2013, chapter 13), upbeat, and the owner of a contagious laugh, Corrie inspires everybody with her trailblazing, glass ceiling- and stereotype-breaking approach to science. Moreover, the discovery of *Corrieopone* only occurred because, back in 2017, Corrie Moreau and Christophe Duplais spurred us to house Ant Course 2018 at Nouragues Research Station in French Guiana. The suffix “pone” is derived from the subfamily name Ponerinae.

**Distribution and ecology.** To date, *Corrieopone* is only known from the Natural Reserve of Nouragues in French Guiana (Figs 1, 2). Specimens were discovered in leaf litter at the base of an *Astrocaryum* palm in an area referred to as Petit Plateau, which is covered by old-growth lowland terra-firme equatorial evergreen forest (Fig. 2F; Poncy et al. 2001). The forest seemed undisturbed, and according to Charles-Dominique (2001), anthropogenic impact has been limited since settlements disappeared from the area in the eighteenth century.

#### ﻿ Corrieopone nouragues

Taxon classificationAnimaliaHymenopteraFormicidae

8CDBCD76-EFFF-5CA1-A830-80354D1BEF82

http://zoobank.org/A82C546A-D3B5-421C-BF00-BFF998F86F02

Figs 7 , 8 , 37 , 38 , 39 , 40 , 41 , 42 , 43 , 44 , 52A , 53 , 54 , 55 , 56 , 57

##### Type locality.

French Guiana: Cayenne, Nouragues Natural Reserve, Nouragues Research Station, Inselberg Station, Petit Plateau grid; 4.08354° N, 52.68368° W, ± 5 m; alt. 145 m; old-growth lowland terra-firme equatorial evergreen forest; in leaf litter at the base of an *Astrocaryum* palm (Arecaceae).

##### Type specimens.

***Holotype***: worker, pinned; “French Guiana: Rés. Nouragues, Inselberg Stn; 4.08354°, –52.68368°, ±5m; 145m; rainforest; hand collection; 29 Aug. 2018; B.L. Fisher, M. Fichaux leg.; BLF41460”; CASC, CASENT0830464. ***Paratypes***: French Guiana • 1 worker; same data as for holotype; JTLC, CASENT0645962 • 1 worker; same data as for holotype; MNHN, CASENT0872028 • 1 worker; same data as for holotype; MZSP, CASENT0872029 • 3 workers; same data as for holotype; CASC, CASENT0830465, CASENT0923158, CASENT0872031 • 1 dealated queen; same data as for holotype; CASC, CASENT0923157.

##### Worker description.

**Measurements** (*N* = 4; holotype values within parentheses): HL: 1.54–1.57 (1.57); HW: 1.33–1.41 (1.41); SL: 1.59–1.67 (1.67); WL: 2.14–2.26 (2.26); TL: 6.54–7.10 (7.06); CI: 87–90 (89); SI: 119–120 (119).

Medium-sized, slender ants (TL 6.54–7.1 mm) with characters as described for *Corrieopone* and the following:

**Figure 54.**
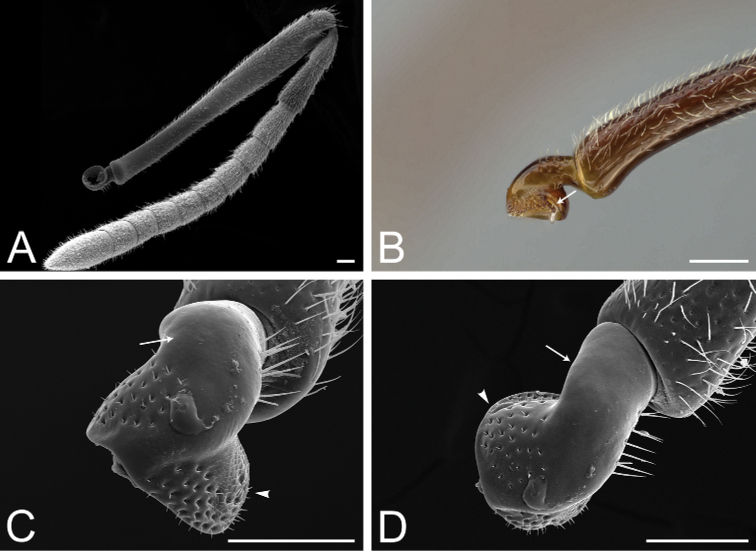
Antenna of *Corrieoponenouragues*; paratypes, worker caste **A** right antenna in ventral view (CASENT0923158) **B** lateral face of the bulbus, bulbus neck, and basal portion of the main shaft of the scape; arrow indicates the notched lateral margin of the bulbus (CASENT0872031) **C** bulbus (arrowhead) and bulbus neck (arrow) in anterior view, with the lateral side facing the right of the image (CASENT0923158) **D** bulbus (indicated by arrowhead) and bulbus neck (indicated by arrow) in dorsal view with lateral side facing the right of the image (CASENT0923158). Images by FA Esteves; available at AntWeb.org. Scale bars: 0.1 mm.

**Color**: reddish brown; lateral surfaces of clypeus and appendages slightly lighter; apex of gaster yellowish (Fig. 37).

**General vestiture**: Head, mesosoma, petiole, and gaster vested with densely arranged, short, subdecumbent to suberect filiform setae, and with much sparser, longer, suberect to erect filiform setae; posterior region of abdominal segment VII with abundant longer setae (Figs 38A, B, 40A, 43A, C). Dorsal face of mandible with sparse, appressed, filiform setae, grading laterad to buoyant, flexuous, longer filiform setae (Fig. 38A, E). Legs dressed with densely arranged, short, subdecumbent to suberect filiform setae.

**Sculpture**: Head in dorsal view mostly punctate (Fig. 8D). Mandibles sparsely punctate, with shallow fossulae skirting the apical two-thirds of the dorsal face of the masticatory margin (Fig. 8C). Clypeus mostly smooth; region adjacent to median area weakly costulate. Pronotum weakly punctate dorsally, grading to costulate laterally (Fig. 8B). Mesonotum weakly punctate. Anepisternum mostly smooth to sparsely costulate (Fig. 40B). Katepisternum costulate dorsally, grading to obliquely strigulate ventrally (Fig. 40B). In profile, metapleuron obliquely strigulate (Fig. 40C). Propodeum strigulate-punctate dorsally; obliquely strigulate laterally (Fig. 40C); declivitous face strigulate laterally, mostly smooth medially (Fig. 8F). Petiolar tergite anterior face mostly smooth; lateral face mostly smooth, sparsely costulate, or confused rugose (Figs 8E, 43A); posterior face mostly smooth dorsally, grading to strigate ventrally (Figs 8F, 43A). Gaster weakly punctate; mostly smooth (Fig. 43C).

**Figure 55.**
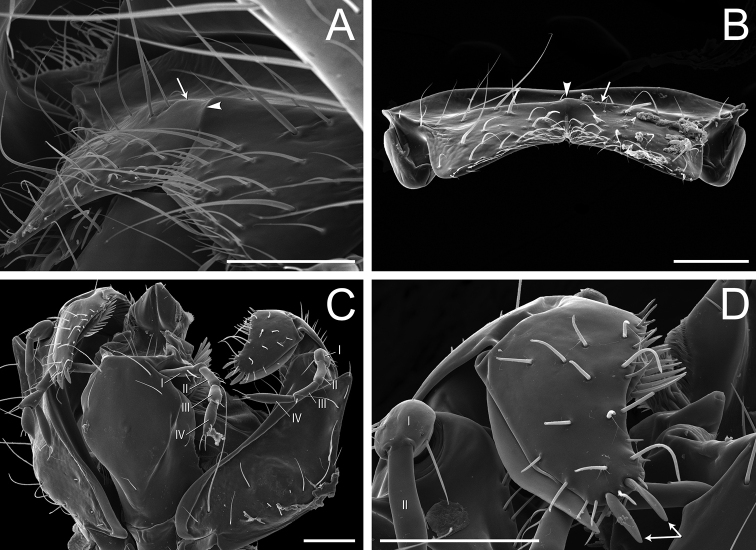
Labrum and maxillolabial complex of *Corrieoponenouragues*; paratypes, worker caste **A** outer face of the labrum in ventrolateral view (CASENT0872031); arrow indicates transverse protrusion; arrowhead indicate median carina **B** outer face of the labrum in apical view (CASENT0923158); arrow indicates transverse protrusion; arrowhead indicate weak median carina **C** outer face of the maxillolabial complex (CASENT0923158); basal towards the bottom of the image; Roman numerals indicate the count of maxillary and labial palpomeres **D** outer face of the galea (CASENT0923158); galeal comb in orange; arrows indicate elliptic seta on the galeal crown; Roman numerals indicate maxillary palpomeres. Images by FA Esteves; available at AntWeb.org. Scale bars: 0.1 mm.

**Head**: Head slightly longer than broad (CI: 87–90). In dorsal view, posterior margin of the head slightly concave medially (Figs 37B, 38A). In profile, posteroventral curve of the head without a projecting flange (Figs 37C, 38B). Mandibles triangular, edentate, devoid of basolateral and dorsal pits or dorsolateral and dorsomasticatory sulci (Figs 37B, 38A, E). Basal three-fourths of the mandibular masticatory margin skirted ventrally by two rows of long, flexuous, helicoid setae, with a row of filiform setae present in between. Mandible with a row of filiform setae running obliquely from the basolateral area of the ventral face to the apex of the lateral face; with setae increasing in length towards mandibular apex; apicalmost seta helicoid. Torular lobes closely approximated (Fig. 38A, F); torular lateral arches visible in dorsal view (Fig. 38F). In profile, torular lobes located at the dorsalmost part of a prominence formed by the clypeal median area and the frontal carinae (Fig. 38B). Lateral arch of the torulus projected dorsad, with a somewhat constricted rim (Fig. 38D, F). Twelve antennomeres (Fig. 54A); bulbus hemispherical, notched on the lateral margin (Fig. 54B); bulbus neck tubular (Fig. 54C, D). Antennal scape (antennomere I) surpassing the posterior margin of the head (SI: 119–120); antennomeres III and IV longer than all other preapical antennomeres save the scape; antennomere III almost as long as the apicalmost antennomere (Fig. 54A). Compound eyes present, located just anterior to the midlength of the head, surrounded by a sulcus; widest diameter of compound eyes: six or seven ommatidia (Fig. 38A, B, D). Ocelli absent. Labrum apically bilobed, with lobes broadly rounded apicolaterally (Fig. 39E); long, acute cleft at midpoint of apical margin. Middle of outer face of the labrum bulging transversely, with a short, median carina extending basad from the median cleft; the carina may be a weak, blunt protrusion or a somewhat developed ridge (Fig. 55A, B). Palpal formula 4,4 (Fig. 55C); basalmost maxillary palpomere short, bulbous. Outer face of maxillary stipes without a transverse sulcus (Fig. 55C). Galeal comb present (Fig. 55D). Galeal crown armed with two or three elliptic setae (Fig. 55D); inner face without a translucid comb of setae (Fig. 55C, D). Premental shield without a transverse sulcus (Fig. 55C).

**Figure 56.**
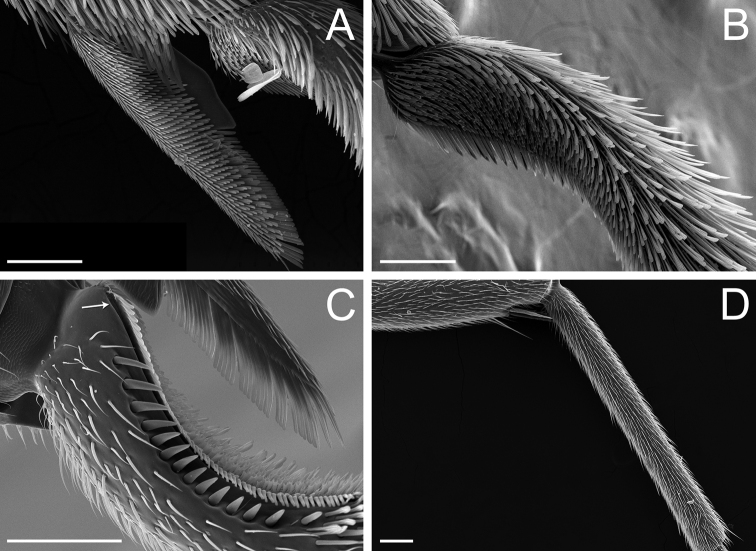
Leg of *Corrieoponenouragues*; paratypes, worker caste **A** calcar of strigil in anterior view (CASENT0872031) **B** probasitarsus in ventroanterior view (CASENT0923158) **C** probasitarsal notch in posterior view (CASENT0923158); arrow indicates longitudinal sulcus next to comb of strigil **D** ventral area of the mesotibia apical half and mesobasitarsus (CASENT0872031). Images by FA Esteves; available at AntWeb.org. Scale bars: 0.1 mm.

**Figure 57.**
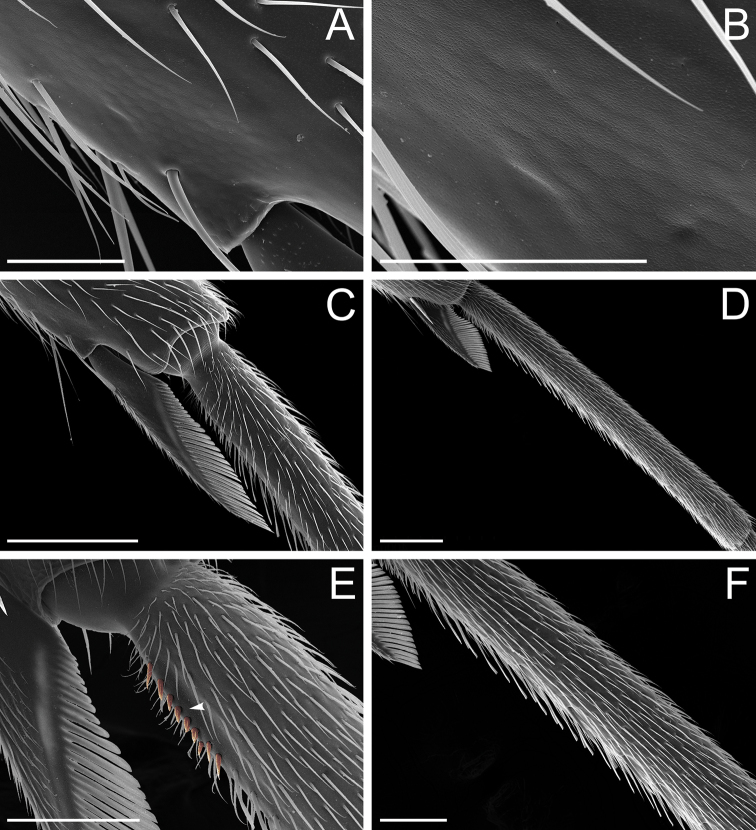
Leg of *Corrieoponenouragues*; paratypes, worker caste **A** colliculate cuticular patch on the posterior face of the metatibial apex (CASENT0872031) **B** close-up of the colliculate cuticular patch on the metatibia (CASENT0872031) **C** posterior metatibial spur in posterior view (CASENT0872031) **D** metatibial spurs and metabasitarsus in anterior view (CASENT0923158) **E** basal portion of the metabasitarsus in anterior view (CASENT0923158); note the short, median carina (arrowhead) armed with a row of short, spine-like setae (colored in orange) **F** portion of the metabasitarsus anterior to its midlength, in anterior view (CASENT0923158). Images by FA Esteves; available at AntWeb.org. Scale bars: 0.05 mm (**A, B**); 0.2 mm (**C, D**); 0.1 mm (**E, F**).

**Mesosoma**: In dorsal view, mesonotum round, wider than the dorsal face of the propodeum but narrower than the pronotum (Fig. 37A); metanotal sulcus mostly smooth, conspicuous. In profile, mesonotum dome-shaped; notopleural suture separating mesonotum from mesopleuron; promesonotum higher than propodeum; metanotal sulcus deeply impressed (Figs 37C, 40A). In profile, mesopleuron divided into anepisternum and katepisternum (Fig. 40A, B). Mesometapleural suture present (Fig. 40A–C). Metathoracic spiracle concealed by a spiracular lobe (Fig. 40A, B). Posterolateral surface of the metapleuron devoid of a longitudinal flange (Fig. 40A, C). Orifice of the metapleural gland round, opening posterolaterad, with ventral margin atop a carina located on the posterolateral margin of the metapleuron (= metapleural carina; Fig. 40C, D). Propodeal dorsum devoid of a median longitudinal impression, propodeal spines absent (Fig. 40A), propodeal posterolateral margin not carinate (Fig. 40A, C). In profile, propodeal lobe rounded, not surpassing posteriorly the dorsoposterior-most point of the rim of the propodeal foramen (Fig. 40C); propodeal spiracle slit-shaped (Fig. 40A, C). Mesosternal process bidentate; metasternal process bilobate, long (Fig. 40F). Metacoxal cavities tightly encircled by unfused annuli (Fig. 40E).

**Legs**: Apico-anterior portion of protibia with a brush of spatulate-costate setae, next to calcar of strigil (Fig. 41A); apicoposterior portion of protibia devoid of stout, spine-like setae (Fig. 41B). Basal third of the ventral margin of the calcar lamellate; lamella glabrous, devoid of any notch (Fig. 41B). From base to apex, microtrichia on anterior face of calcar grades from tubiform to spatulate (Fig. 56A); posterior face of calcar with lanceolate microtrichia (Fig. 41B). Anterior and ventral faces of probasitarsus densely vested with spatulate-costate setae (Fig. 41A), except for the area immediately anterior to the comb of strigil, which bears shorter, spatulate-bicuspid setae (Fig. 56B). Comb of strigil skirted posteriorly by a sulcus (Fig. 56C). Row of stout, spine-like setae on posterior face of probasitarsal notch, parallel to comb of strigil (Fig. 56C). Counting from base to apex: anterior face of the second tarsomere of foreleg with spatulate-costate setae; ventral faces of second, third, and fourth tarsomeres with stout, spine-like setae (Fig. 42C); fourth tarsomere cylindrical. Mesotibia with two spurs (Fig. 41C); anterior spur simple (Fig. 56D); posterior spur serrate, with lanceolate microtrichia on dorsal face (Fig. 41D). Mesobasitarsus devoid of any longitudinal sulcus (Fig. 56D); two rows of spine-like setae present along apical half of ventral face of mesobasitarsus and along entire ventral face of second, third, and fourth tarsomeres (Fig. 42D). Metacoxal dorsum devoid of any spine-like projection. Posterior portion of metatibial apex with a glabrous, yellowish, oblong, weakly colliculate cuticular patch (Figs 41E, 57A, B); cuticular patch with densely arranged, minute pores (visible under magnifications above 4700×; Fig. 57B); pores round to slit-shaped (~0.29 um length; visible at 8500× magnification); cuticle surrounding oblong cuticular patch with sparser, longer slit-shaped pores (~0.45 um length). Ventral face of metatibial apex with two spurs (Fig. 57D); anterior spur simple and glabrous. Metatibial posterior spur pectinate; with lanceolate microtrichia on anterior face; with mostly glabrous posterior face, except for lanceolate microtrichia on dorsal surface (Fig. 57C, D). Metabasitarsus devoid of any longitudinal sulcus (Fig. 57D). Midline of the ventrobasal portion of metabasitarsus with a short, longitudinal carina armed with a row of short, spine-like setae (Fig. 57E); the carina is followed apically by a longitudinal row of spatulate setae that extends to the midlength of the segment (Fig. 57F); ventral face of metabasitarsus with the apical two-thirds bearing two rows of spine-like setae that skirt the midline row of spatulate setae. Dorsal faces of the mid- and hindlegs devoid of stout, spine-like setae (Fig. 42A, B). Ventral faces of the second, third, and fourth tarsomeres with two rows of spine-like setae that skirt the midline of each segment (Fig. 42E). Simple pretarsal claws on fore-, mid-, and hindlegs (Fig. 42C–F). Arolium indistinct on pro-, meso-, and metapretarsi (Fig. 42C–F).

**Petiole**: Sessile; surmounted by a high, unarmed, conic scale-like node that is narrow in profile and in dorsal view (Figs 37A, C, 43A). Anteroventral portion of the tergite with a lateral carina (Fig. 43A); spiracle orifice directed dorsolaterally; posterior face of the tergite with the ventral portion strigate; laterotergite delineated by a longitudinal suture (Fig. 43B). Subpetiolar process keel-like, with a round anteroventral projection (Fig. 43A), fenestra absent. Anterior disc of sternite with a round proprioceptor zone (Fig. 43B). Sternite without a posterior spatulate projection (Fig. 43B), articulation with the helcium visible in ventral view.

**Gaster**: Helcium infra-axial: positioned ventrad to the midheight of the anterior face of abdominal segment III (Fig. 43C). Prora well-developed on the anterior face of the sternite of abdominal segment III, lip-shaped (Fig. 43C, D). Abdominal segment IV tubular: tergite and sternite with similar lengths, tergite not arched (Fig. 43C). Presclerites of the abdominal segment IV forming an even surface with postsclerites (Fig. 43C), no girdling constriction. Small stridulitrum present on the pretergite of the abdominal segment IV (Fig. 43E, F). Pygidium not armed with spine-like setae, no acute cuticular projections, dorsum convex. Hypopygium as described for the genus (Fig. 44).

##### Queen description.

**Measurements** (*N* = 1): HL: 1.65; HW: 1.54; SL: 1.74; WL: 2.51; TL: 8.48; CI: 93; SI: 113.

Dealate. Color dark brown; clypeus and appendages lighter, reddish; apex of gaster yellowish (Fig. 53A–D). Similar to the worker caste but for the darker body color and slightly larger body length (TL 8.48), larger compound eyes, presence of ocelli, and differences in the mesosoma due to the presence of wings (Fig. 53). As described for the genus.

##### Etymology.

The specific epithet *nouragues* is a non-Latin noun in apposition. It honors the Nouragues (or Norak) people, a Tupi Amerindian population who once inhabited the area (Grenand 1982), and the Nouragues Natural Reserve and Research Station.

## Supplementary Material

XML Treatment for Corrieopone

XML Treatment for Corrieopone nouragues

## Supplementary materials

Supplementary material 1 Table S1 Data type Specimen data. Explanation note Here , we provide specimen data of material examined (unique identifiers, collection and specimen information, depositories, and links to each specimen webpage on AntWeb.org). This material was used to contrast the morphology of *Corrieoponenouragues* gen. nov. with 129 species or subspecies representing all 47 currently valid extant Ponerinae genera (see also Tables S2, S3). https://binary.pensoft.net/file/617799This dataset is made available under the Open Database License (http://opendatacommons.org/licenses/odbl/1.0/). The Open Database License (ODbL) is a license agreement intended to allow users to freely share, modify, and use this Dataset while maintaining this same freedom for others, provided that the original source and author(s) are credited.Flavia A. Esteves, Brian L. Fisher

Supplementary material 2 Table S2 Data type Мorphological. Explanation note Here, we list and define morphological characters used to compare the morphology of *Corrieoponenouragues* gen. nov., sp. nov. with other Ponerinae genera. When producing this list and the associated character matrix (Table S3), our goals were to document and organize our observations. It only contains characters which we could unambiguously discretize. We share it to disclose our methodology and foster validation, replication, and reinterpretation of our results. https://binary.pensoft.net/file/617800This dataset is made available under the Open Database License (http://opendatacommons.org/licenses/odbl/1.0/). The Open Database License (ODbL) is a license agreement intended to allow users to freely share, modify, and use this Dataset while maintaining this same freedom for others, provided that the original source and author(s) are credited.Flavia A. Esteves, Brian L. Fisher

Supplementary material 3 Table S3 Data type Morphological. Explanation note Table S3 documents states of characters we evaluated across ponerine genera (see character statements in Table S2). When producing this matrix, our goals were to document and organize our observations. It only contains characters which we could unambiguously discretize. We share it to disclose our methodology and foster validation, replication, and reinterpretation of our results. Character numbers correspond to those in *Corrieopone* genus diagnosis. https://binary.pensoft.net/file/617801This dataset is made available under the Open Database License (http://opendatacommons.org/licenses/odbl/1.0/). The Open Database License (ODbL) is a license agreement intended to allow users to freely share, modify, and use this Dataset while maintaining this same freedom for others, provided that the original source and author(s) are credited.Flavia A. Esteves, Brian L. Fisher

Supplementary material 4 Table S4 Data type Morphometric. Explanation note All measurements were taken at 80× power with a Leica MZ125 microscope using an orthogonal pair of micrometers, recorded to the nearest 0.001 mm, and rounded to two decimal places, as following. Head length (HL): maximum longitudinal length of the head, measured from the anteriormost portion of the projecting clypeus to the midpoint of an imaginary line traced across the posterior margin of the head. Head width (HW): maximum width of head, excluding eyes. Scape length (SL): maximum chord length of the main shaft of the antennal scape, excluding basal bulbus and bulbus neck. Weber’s length (WL): with the mesosoma in profile, the diagonal length from the posteroventral corner of the propodeum to the farthest point on the anterior face of the pronotum, excluding the neck. Total length (TL): sum of HL + WL + length of segments A2 to A7. A2 to A7 is measured as follows: maximum length of the petiole in profile (PL) + A3 to A7, or gaster length (GL). Cephalic index (CI): HW/HL × 100. Scape index (SI): SL/HW × 100. https://binary.pensoft.net/file/617802This dataset is made available under the Open Database License (http://opendatacommons.org/licenses/odbl/1.0/). The Open Database License (ODbL) is a license agreement intended to allow users to freely share, modify, and use this Dataset while maintaining this same freedom for others, provided that the original source and author(s) are credited.Flavia A. Esteves, Brian L. Fisher

## References

[B1] AntCat (2021) An online catalog of the ants of the world. http://antcat.org. [Accessed: March 01, 2021]

[B2] Arimoto K Yamane S (2018) Taxonomy of the *Leptogenyschalybaea* species group (Hymenoptera, Formicidae, Ponerinae) from Southeast Asia. Asian Myrmecology 10: e010008. 10.20362/am.010008

[B3] Baroni Urbani C (1975b) Contributo alla conoscenza dei generi *Belonopelta* Mayr e *Leiopelta* gen. n. (Hymenoptera: Formicidae). Mitteilungen der Schweizerischen Entomologischen Gesellschaft 48: 295–310. 10.5281/zenodo.26787

[B4] Bernard F (1967) Faune de l’Europe et du Bassin Méditerranéen 3. Les fourmis (HymenopteraFormicidae) d’Europe occidentale et septentrionale. Masson, Paris, 411 pp.

[B5] Billen J (2009) Occurrence and structural organization of the exocrine glands in the legs of ants. Arthropod Structure & Development 38: 2–15. 10.1016/j.asd.2008.08.002 18775512

[B6] Bolton B (1975) A revision of the ant genus *Leptogenys* Roger (Hymenoptera: Formicidae) in the Ethiopian region with a review of the Malagasy species. Bulletin of the British Museum (Natural History). Entomology 31: 235–305. 10.5962/bhl.part.29487

[B7] Bolton B Brown Jr WL (2002) *Loboponera* gen. n. and a review of the Afrotropical *Plectroctena* genus group (Hymenoptera: Formicidae). Bulletin of the Natural History Museum. Entomology Series 71: 1–18. 10.1017/S0968045402000019

[B8] Bolton B (2003) Synopsis and classification of Formicidae. Memoirs of the American Entomological Institute 71. American Entomological Institute, Gainesville, 370 pp.

[B9] Bolton B Fisher BL (2008a) The Afrotropical ponerine ant genus *Phrynoponera* Wheeler (Hymenoptera: Formicidae). Zootaxa 1892: 35–52. 10.11646/zootaxa.1892.1.3

[B10] Bolton B Fisher BL (2008b) Afrotropical ants of the ponerine genera *Centromyrmex* Mayr, *Promyopias* Santschi gen. rev. and *Feroponera* gen. n., with a revised key to genera of African Ponerinae (Hymenoptera: Formicidae). Zootaxa 1929: 1–37. 10.11646/zootaxa.1929.1.1

[B11] Boudinot BE Perrichot V Chaul JCM (2020) *Camelosphecia* gen. nov., lost ant-wasp intermediates from the mid-Cretaceous (Hymenoptera, Formicoidea). ZooKeys 1005: 21–55. 10.3897/zookeys.1005.57629 33390754 PMC7762752

[B12] Boudinot BE Moosdorf OTD Beutel RG Richter A (2021) Anatomy and evolution of the head of *Dorylushelvolus* (Formicidae: Dorylinae): Patterns of sex- and caste-limited traits in the sausagefly and the driver ant. Journal of Morphology 282: 1616–1658. 10.1002/jmor.21410 34427942

[B13] Brown Jr WL (1963) Characters and synonymies among the genera of ants. Part III. Some members of the tribe Ponerini (Ponerinae, Formicidae). Breviora 190: 1–10. 10.5281/zenodo.26976

[B14] Charles-Dominique P (2001) The Field Station. In: Bongers F Charles-Dominique P Forget PM Théry M (Eds) Nouragues: Dynamics and plant-animal interactions in a Neotropical rainforest.Monographiae Biologicae, vol. 80. Springer, Dordrecht, 1–8. 10.1007/978-94-015-9821-7

[B15] Collingwood CA Agosti D (1996) Formicidae (Insecta: Hymenoptera) of Saudi Arabia (part 2). Fauna of Saudi Arabia 15: 300–385. https://ia802304.us.archive.org/23/items/ants_08420/8420_text.pdf

[B16] D’Esquivel MS Jahyny BJB Oliveira ML Lacau LSR Delebie JHC Lacau S (2017) *Thaumatomyrmexfraxini* sp. nov. (Hymenoptera: Formicidae), a new ant species from the Brazilian Atlantic Forest. Sociobiology 64(2): 159–165. 10.13102/sociobiology.v64i2.1615

[B17] Dinerstein E Olson DP Joshi A Vynne C Burgess N Wikramanayake E Hahn N Palminteri S Hedao P Noss R Hansen M Locke H Ellis EC Jones BS Barber CV Hayes R Kormos C Martin V Crist E Sechrest W Price L Baillie J Weeden D Suckling KF Davis C Sizer N Moore R Thau D Birch T Potapov P Turubanova S Tyukavina A Souza ND Pintea L Brito JC Llewellyn O Miller AG Patzelt A Ghazanfar S Timberlake J Klöser H Shennan-Farpón Y Kindt R Lillesø JP Breugel PV Graudal L Voge M Al-Shammari KF Saleem M (2017) An ecoregion-based approach to protecting half the terrestrial realm. Bioscience 67: 534–545. 10.1093/biosci/bix014 28608869 PMC5451287

[B18] Fernandes IO Oliveira ML de Delabie JHC (2014) Description of two new species in the Neotropical *Pachycondylafoetida* complex (Hymenoptera: Formicidae: Ponerinae) and taxonomic notes on the genus. Myrmecological News 19: 133–163. https://www.myrmecologicalnews.org/cms/index.php?option=com_content&view=category&id=589&Itemid=363

[B19] Fernandes IO Delabie JHC (2019) A new species of *Cryptopone* Emery (Hymenoptera: Formicidae: Ponerinae) from Brazil with observations on the genus and a key for new word species. Sociobiology 66: 408–441. 10.13102/sociobiology.v66i3.4354

[B20] Fisher BL (2006) *Boloponeravicans* gen.n. and sp.n. and two new species of the *Plectroctena* genus group (Hymenoptera: Formicidae). Myrmecologische Nachrichten 8: 111–118. http://antbase.org/ants/publications/21106/21106.pdf

[B21] Fisher BL Guenard BS Robson S (2015) Borneo, fANTastique! Asian Myrmecology 7: 171–174. 10.20362/am.007018

[B22] Fisher BL Bolton B (2016) Ants of Africa and Madagascar: A guide to the genera. University of California Press, Berkeley, 514 pp. 10.1525/9780520962996

[B23] Forel A (1910) Ameisen aus der Kolonie Erythräa. Gesammelt von Prof. Dr. K. Escherich (nebst einigen in West-Abessinien von Herrn A. Ilg gesammelten Ameisen). Zoologische Jahrbücher. Abteilung für Systematik, Geographie und Biologie der Tiere 29: 243–274. https://www.zobodat.at/pdf/Zoologische-Jahrbuecher-Syst_29_0243-0274.pdf

[B24] Forel A (1913) Wissenschaftliche Ergebnisse einer Forschungsreise nach Ostindien ausgeführt im Auftrage der Kgl. Preuss. Akademie der Wissenschaften zu Berlin von H. v. Buttel-Reepen. II. Ameisen aus Sumatra, Java, Malacca und Ceylon. Gesammelt von Herrn Prof. Dr. v. Buttel-Reepen in den Jahren 1911–1912. Zoologische Jahrbücher. Abteilung für Systematik, Geographie und Biologie der Tiere 36: 1–148. https://biostor.org/reference/181439

[B25] Gibson GAP (1985) Some pro- and mesothoracic structures important for phylogenetic analysis of Hymenoptera, with a review of the terms used for the structures. Canadian Entomologist 117: 1395–1443. 10.4039/Ent1171395-11

[B26] Gibson GAP Read JD Fairchild R (1998) Chalcid wasps (Chalcidoidea): Illustrated glossary of positional and morphological terms. http://www.canacoll.org/Hym/Staff/Gibson/apss/chglintr.htm

[B27] Gibson JC Larabee FJ Touchard A Orivel J Suarez AV (2018) Mandible strike kinematics of the trap-jaw ant genus *Anochetus* Mayr (Hymenoptera: Formicidae). Journal of Zoology 306: 119–128. 10.1111/jzo.12580

[B28] Greenberg JA Mattiuzzi M (2020) gdalUtils: Wrappers for the Geospatial Data Abstraction Library (GDAL) Utilities. R package version 2.0.3.2. https://CRAN.R-project.org/package=gdalUtils

[B29] Grenand P (1982) Ainsi parlaient nos ancêtres. Essai d’ethnohistoire Wayapi. ORSTOM, Paris, 408 pp.

[B30] Grimaldi M Riéra B (2001) Geography and Climate. In: Bongers F Charles-Dominique P Forget PM Théry M (Eds) Nouragues: Dynamics and plant-animal interactions in a Neotropical rainforest.Monographiae Biologicae, vol. 80. Springer, Dordrecht, 9–18. 10.1007/978-94-015-9821-7

[B31] Hansen MC Potapov PV Moore R Hancher M Turubanova SA Tyukavina A Thau D Stehman SV Goetz SJ Loveland TR Kommareddy A Egorov A Chini L Justice CO Townshend JRG (2013) High-Resolution Global Maps of 21^st^-Century Forest Cover Change. Science 342(6160): 850–853. 10.1126/science.1244693 24233722

[B32] Harris RA (1979) A glossary of surface sculpturing. California Department of Food and Agriculture, Bureau of Entomology 28: 1–31. 10.5281/zenodo.26215

[B33] Hawkes PG (2010) A new species of *Asphinctopone* (Hymenoptera: Formicidae: Ponerinae) from Tanzania. Zootaxa 2480: 27–36. 10.11646/zootaxa.2480.1.2

[B34] Hijmans R (2021) raster: Geographic Data Analysis and Modeling. R package version 3.4–13. https://CRAN.R-project.org/package=raster

[B35] Hölldobler B Obermayer M Peeters C (1996) Comparative study of the metatibial gland in ants (Hymenoptera, Formicidae). Zoomorphology 116: 157–167. 10.1007/BF02527156

[B36] Keller RA (2011) A phylogenetic analysis of ant morphology (Hymenoptera: Formicidae) with special reference to the poneromorph subfamilies. Bulletin of the American Museum of Natural History 355: 1–90. 10.1206/355.1

[B37] Kempf WW (1960) Miscellaneous studies on Neotropical ants (Hymenoptera, Formicidae). Studia Entomologica 3: 417–466. 10.5281/zenodo.26016

[B38] Kempf WW (1961a) As formigas do gênero *Pachycondyla* Fr. Smith no Brasil (Hymenoptera: Formicidae). Revista Brasileira de Entomologia 10: 189–204. 10.5281/zenodo.26019

[B39] Kempf WW (1961b) A survey of the ants of the soil fauna in Surinam (Hymenoptera: Formicidae). Studia Entomologica 4: 481–524. 10.5281/zenodo.26018

[B40] Kempf WW (1964) Miscellaneous studies on Neotropical ants III (Hymenoptera: Formicidae). Studia Entomologica 7: 45–71. 10.5281/zenodo.26030

[B41] Kempf WW (1975) A revision of the Neotropical ponerine ant genus *Thaumatomyrmex* Mayr (Hymenoptera: Formicidae). Studia Entomologica 18: 95–126. 10.5281/zenodo.26052

[B42] Kronauer DJC (2004) Trophic parasitism of a wasp (Hymenoptera: Ampulicidae: *Ampulex* sp.) on the ant *Ectatommaruidum* (Roger, 1860) (Hymenoptera: Formicidae). Myrmecologische Nachrichten 6: 77–78. https://myrmecologicalnews.org/cms/index.php?option=com_content&view=category&id=153&Itemid=350

[B43] LaPolla J Cover S Mueller U (2002) Natural history of the mealybug-tending ant, *Acropygaepedana*, with descriptions of the male and queen castes. Transactions of the American Entomological Society 128: 367–376. https://antwiki.org/wiki/images/d/d2/Cover_2002.pdf

[B44] Larabee FJ Gronenberg W Suarez AV (2017) Performance, morphology and control of power-amplified mandibles in the trap-jaw ant *Myrmoteras* (Hymenoptera: Formicidae). Journal of Experimental Biology 220: 3062–3071. 10.1242/jeb.156513 28855320

[B45] Larabee FJ Smith AA Suarez AV (2018) Snap-jaw morphology is specialized for high-speed power amplification in the Dracula ant, *Mystriumcamillae*. Royal Society Open Science 5: e181447. 10.1098/rsos.181447 PMC6304126 30662749

[B46] Lattke JE Delsinne T (2016) Revisionary and natural history notes on some species of the genus *Gnamptogenys* Roger, 1863 (Hymenoptera: Formicidae). Myrmecological News 22: 141–147. https://myrmecologicalnews.org/cms/index.php?option=com_content&view=category&id=635&Itemid=366

[B47] Lehner B Döll P (2004) Development and validation of a global database of lakes, reservoirs and wetlands. Journal of Hydrology 296: 1–22. 10.1016/j.jhydrol.2004.03.028

[B48] Longino JT Branstetter MG (2020) Phylogenomic species delimitation, taxonomy, and ‘bird guide’ identification of the Neotropical ant genus *Rasopone* (Hymenoptera: Formicidae). Insect Systematics and Diversity 4: 1–33. 10.1093/isd/ixaa004

[B49] Mackay WP Mackay E (2010) The systematics and biology of the New World ants of the genus *Pachycondyla* (Hymenoptera: Formicidae). Edwin Mellen Press, Lewiston (New York): 642 pp. 10.13140/2.1.4271.8726

[B50] Markl H (1973) The evolution of stridulatory communication in ants. In: Butler CG Howse PE (Eds) Proceedings of the International Union for the Study of Social Insects.VII International Congress, London, September 1973. University of Southampton, Southampton, 258–265. https://cataglyphis.fr/Actes-SF-UIEIS/IUSSI-Londres-1973/VII-Congres-IUSSI-Londres-Septembre-1973.htm

[B51] Mayr G (1901) Südafrikanische Formiciden, gesammelt von Dr. Hans Brauns. Annalen des Kaiserlich-Königlichen Naturhistorischen Museums in Wien 16: 1–30. 10.5281/zenodo.25873

[B52] NASA JPL (2013) NASA Shuttle Radar Topography Mission Water Body Data Shapefiles & Raster Files. Distributed by NASA EOSDIS Land Processes DAAC. 10.5067/MEaSUREs/SRTM/SRTMSWBD.003

[B53] NASA JPL (2020) NASADEM Merged DEM Global 1 arc second V001, distributed by NASA EOSDIS Land Processes DAAC. 10.5067/MEaSUREs/NASADEM/NASADEM_HGT.001

[B54] Ouellet Dallaire C Lehner B Sayre R Thieme M (2019) A multidisciplinary framework to derive global river reach classifications at high spatial resolution. Environmental Research Letters 14(2): e024003. 10.1088/1748-9326/aad8e9

[B55] Padgham M Rudis B Lovelace R Salmon M (2017) osmdata. Journal of Open Source Software 2(14): e305. 10.21105/joss.00305

[B56] Pebesma E (2018) Simple features for R: Standardized support for spatial vector data. The R Journal 10: 439–446. 10.32614/RJ-2018-009

[B57] Peeters C (2017) Independent colony foundation in *Paraponeraclavata* (Hymenoptera: Formicidae): First workers lay trophic eggs to feed queen’s larvae. Sociobiology 64: 417–422. 10.13102/sociobiology.v64i4.2092

[B58] Peeters C Fuminori I Wiwatwitaya D Keller RA Hashim R Molet M (2017) Striking polymorphism among infertile helpers in the arboreal ant *Gesomyrmex*. Asian Myrmecology 9: e009015. 10.20362/am.009015

[B59] Poncy O Sabatier D Prevost MF Hardy I (2001) The lowland high forest: structure and tree species diversity. In: Bongers F Charles-Dominique P Forget PM Théry M (Eds) Nouragues: Dynamics and plant-animal interactions in a Neotropical rainforest.Monographiae Biologicae, vol. 80. Springer, Dordrecht, 31–46. 10.1007/978-94-015-9821-7

[B60] R Core Team (2021) R: A language and environment for statistical computing. R Foundation for Statistical Computing, Vienna, Austria. URL https://www.R-project.org/

[B61] Rakotonirina JC Fisher BL (2013) Revision of the *Pachycondylasikorae* species-group (Hymenoptera: Formicidae) in Madagascar. Zootaxa 3683: 447–485. 10.11646/zootaxa.3683.4.8 25250464

[B62] Richards OW (1977) Hymenoptera. Introduction and key to families. Second edition. Handbooks for the Identification of British Insects, Vol. 6, Part 1. Royal Entomological Society of London, London, 100 pp. https://www.royensoc.co.uk/sites/default/files/Vol06_Part01_ed2.pdf

[B63] RStudio Team (2021) RStudio: Integrated development environment for R. RStudio, PBC, Boston, MA. http://www.rstudio.com

[B64] Schmidt CA (2013) Molecular phylogenetics of ponerine ants (Hymenoptera: Formicidae: Ponerinae). Zootaxa 3647: 201–250. 10.11646/zootaxa.3647.2.1 26295106

[B65] Schmidt CA Shattuck SO (2014) The higher classification of the ant subfamily Ponerinae (Hymenoptera: Formicidae), with a review of ponerine ecology and behavior. Zootaxa 3817: 1–242. 10.11646/zootaxa.3817.1.1 24943802

[B66] Snodgrass RE (1910) The thorax of the Hymenoptera. Proceedings of the United States National Museum 39: 37–91. 10.5479/si.00963801.39-1774.37

[B67] South A (2011) rworldmap: A new R package for mapping global data. The R Journal 3(1): 35–43. 10.32614/RJ-2011-006

[B68] South A (2012) rworldxtra: Country boundaries at high resolution. R package version 1.01. https://CRAN.R-project.org/package=rworldxtra

[B69] Tennekes M (2018) tmap: Thematic maps in R. Journal of Statistical Software 84(6): 1–39. 10.18637/jss.v084.i0630450020

[B70] Ward PS (1990) The ant subfamily Pseudomyrmecinae (Hymenoptera: Formicidae): generic revision and relationship to other formicids. Systematic Entomology 15: 449–489. 10.1111/j.1365-3113.1990.tb00077.x

[B71] Wickham H François R Henry L Müller K (2021) . dplyr: A grammar of data manipulation. R package version 1.0.7. 10.1007/978-1-4842-6876-6_1

[B72] Willey RB Brown Jr WL (1983) New species of the ant genus *Myopias* (Hymenoptera: Formicidae: Ponerinae). Psyche 90: 249–285. 10.5281/zenodo.25285

[B73] Wilson EO (2013) Letters to a young scientist. Liveright, New York, 244 pp.

[B74] Yamane S (2007) *Pachycondylanigrita* and related species in Southeast Asia. In: Snelling RR Fisher BL Ward PS (Eds) Advances in ant systematics (Hymenoptera: Formicidae): homage to E.O. Wilson – 50 years of contributions. Memoirs of the American Entomological Institute, 80. American Entomological Institute, Gainesville, 650–663.

[B75] Yoder MJ Mikó I Seltmann KC Bertone MA Deans AR (2010) A Gross Anatomy Ontology for Hymenoptera. PLoS ONE 5: e15991. 10.1371/journal.pone.0015991 PMC3012123 21209921

