# Supplementary material for: ﻿Corrieoponenouragues gen. nov., sp. nov., a new Ponerinae from French Guiana (Hymenoptera, Formicidae)
Source: Zookeys. 2021 Dec 3;1074:83–173. doi: 10.3897/zookeys.1074.75551 (PMC8671710; doi:10.3897/zookeys.1074.75551)
Supplement: Supplementary material 2 — Table S2 [file zookeys-1074-083-s002.pdf]

Supplementary Material for:

Corrieopone nouragues gen. nov., sp. nov.: a new Ponerinae from French Guiana (Hymenoptera, Formicidae)

Flavia A. Esteves*, Brian L. Fisher

Entomology, California Academy of Sciences, 55 Music Concourse Drive, San Francisco, CA 94118, U.S.A.
* Corresponding author. E-mail: <flaviaesteves@gmail.com>

Table S2. List of morphological characters

Below, we list characters used to compare the morphology of *Corrieopone nouragues* gen. nov., sp. nov. with other ponerine genera. When producing this list and the associated character matrix (S3 Character matrix), our goals were to document and organize our observations. It only contains characters which we could unambiguously discretize. We share it to disclose our methodology and foster validation, replication, and reinterpretation of our results.

The following enumeration corresponds to character numbers in *Corrieopone* genus diagnosis.

2. Mandibles, dentition: (0) absent, (1) present.
An edentate mandible (state 0) is devoid of teeth, denticles, or a projected apex.

3.1. Mandibles, basolateral pit: (0) absent, (1) present.
The basal pit (= basal pit in Fisher and Bolton 2016) is a round, oval, or oblong impression on the basolateral area of the mandible, as seen in *Brachyponera* species.

3.2. Mandibles, dorsal pit: (0) absent, (1) present.
The dorsal face of the mandible in some Ponerinae may present an oblong/oval groove (state 1, as in *Dolioponera fustigera*).

3.3. Mandibles, dorsolateral sulcus (modified from Keller 2011, char. 30): (0) absent, (1) present.
This sulcus runs obliquely along the mandible, from the basal portion of the dorsal face towards the lateral face. It is widespread among the Ponerinae, and may be shallowly or deeply impressed, restricted to the basal portion of the mandible, or present along almost the entire lateral face of the mandible.

3.4. Mandibles, dorsomasticatory sulcus: (0) absent, (1) present.
If present, it is a long and narrow sulcus that borders the masticatory margin on the dorsal face of the mandible. The sulcus may occur on the proximal or distal areas of the mandible (as in *Plectroctena anops* and *P. strigosa*, respectively), or

along most of the masticatory margin (as in *P. cristata*). See also Fisher and Bolton (2016).

6. Torular lobes, degree of connection between median and lateral arches of torulus (as in Keller 2011, char. 08): (0) median arch anteriorly and posteriorly continuous with the lateral arch; (1) median arch anteriorly continuous and posteriorly discontinuous with the lateral arch; (2) median arch anteriorly and posteriorly discontinuous with the lateral arch.

9. Ocelli (as in Keller 2011, char. 48): (0) absent, (1) present.

11. Palpal formula: number of maxillary palps, number of labial palps.

12. Mesonotum, round, dome-shaped outline in profile (modified from Keller 2011: char. 52): (0) absent, (1) present.
If present (state 1), the mesonotum has a somewhat round dorsal margin in profile, which interrupts the outline of the mesosoma (as in *Brachyponera sennaarensis*). Otherwise (state 0), the mesonotum is flat and continuous with the outline of the mesosoma (as in *Pachycondyla striata*), or has an irregular outline (as in *Simopelta transversa*).

13. Notopleural suture (as in Keller 2011, char. 53): (0) absent, (1) present.

14. Metanotal sulcus (as in Keller 2011, char. 57): (0) obliterated, (1) shallowly or deeply impressed groove-like impression.

15. Mesopleuron, division into anepisternum and katepisternum (as in Keller 2011, char. 55): (0) absent, (1) present.

16. Metathoracic spiracle (as in Keller 2011, char. 56): (0) not concealed by lobe, (1) concealed by lobe.

20. Propodeum, dorsoposterior projections: (0) absent, (1) present.
If present (state 1) and seen in profile, the propodeal dorsoposterior corner bears an acute projection, which may be short or long.

22. Propodeal spiracle, shape (as in Keller 2011, char. 65): (0) slit-shaped, (1) round to oval.

24. Metacoxal cavities (modified from Keller 2011, char. 69, and Fisher and Bolton 2016): (0) closed, with annulus fused and uninterrupted; (1) open, with annulus not fused, (2) open, with annular gap.
If closed (state 0), the medial surface of the metacoxal acetabulum does not have a fenestra, and thus, the metacoxal cavity is not connected internally with the propodeal foramen; and the annulus externally encircling the cavity is fused (as in *Platythyrea cribrinodis*). If open, the medial internal surface of the cavity is

fenestrate and connects with the propodeal foramen, and the annulus is unfused. In this case, the annulus may encircle the cavity with its free ends overlapping next to the propodeal foramen (state 1, as in *Corrieopone nouragues*); or there may be a gap in the annulus (state 2, as in *Platythyrea punctata*).

25. Calcar of strigil, basoventral lamella (as in Keller 2011, char. 74): (0) absent, (1) present.

27. Probasitarsus, row of spine-like setae extending along the posterior face of the probasitarsal notch (modified from Keller 2011, char. 72): (0) absent, (1) present.
Most Ponerinae present a row of stout, spine-like setae that skirts the posterior margin of the comb of strigil. The definition of "row" adopted here is analogous to a line that requires at least two points in space to exist. Thus, the row is present (state 1) if two or more spine-like setae align longitudinally along the posterior face of the probasitarsal notch; if there are fewer than two setae, the row is absent (state 0).

28.1. Anterior mesotibial spur, shape: (0) absent, (1) simple, (2) serrate, (3) pectinate.
Most ponerine genera present two mesotibial spurs, but some taxa lack the anterior spur, and a few are devoid of both spurs on the mesotibia. If present, the anterior spur may be spike-shaped, long, or minute, and lack teeth-like or finger-like projections serially-arranged along its ventral margin (state 1); or it may be serrate, and its ventral margin bears a series of small, sharp, teeth-like projections (state 2); or it may be pectinate, with longer, finger-shaped projections rising from its ventral margin (state 3).

28.2. Posterior mesotibial spur, shape: (0) absent, (1) simple, (2) serrate, (3) pectinate.
Definition analogous to that of char. 28.1.

29. Metatibia, cuticular patch on apicoposterior surface: (0) absent, (1) present.
If present (state 1), the metatibial cuticular patch is an oblong, glabrous, distinctly colored, smooth or distinctly sculptured area on the posterior surface of the metatibial apex (as in *Corrieopone nouragues*). If absent (state 0), the posterior surface of the apex of the metatibia is uniformly vested, colored, and sculptured, or presents a brush of distinctly-shaped setae next to the posterior metatibial spur.

30.1. Anterior metatibial spur, shape: (0) absent, (1) simple, (2) serrate, (3) pectinate.
Definition analogous to that of char. 28.1.

30.2. Posterior metatibial spur: (0) absent, (1) simple, (2) serrate, (3) pectinate.
Definition analogous to that of char. 28.1.

31.1. Mesotibia, stout, spine-like setae on anterodorsal surface: (0) absent, (1) present.
If present (state 1), the setae are clearly stouter than the regular, filiform setae also present on the anterodorsal surface of the mesotibia (as in *Cryptopone gilva*). If

absent (state 0), the anterodorsal face of the mesotibia is uniformly vested (as in *Dinoponera lucida*).

31.2. Mesobasitarsus, stout, spine-like setae on outer face: (0) absent, (1) present.
Definition analogous to that of char. 31.1.

31.3. Metatibia, stout, spine-like setae on outer face: (0) absent, (1) present.
Definition analogous to that of char. 31.1.

31.4. Metabasitarsus, stout, spinelike setae on outer face: (0) absent, (1) present.
Definition analogous to that of char. 31.1.

33.1. Propretarsal claws, shape (modified from Keller 2011, char. 82): (0) simple, (1) basal tooth, (2) pre-apical tooth, (3) pectinate.
Simple claws (state 0) may lack any prominences or may present a basal blunt angle or rounded swell (as in *Corrieopone nouragues*). A claw with a basal tooth (state 1) bears an acute prominence on the basal third of its inner margin. The basal tooth overhangs the outline of the inner margin of the claw (as in *Pachycondyla procidua*), and may be armed with several minute, acute projections (as in *Bothroponera pachyderma*). A claw with a preapical tooth (state 2) has an acute projection rising from the apical two-thirds of its inner margin (as in *Dinoponera lucida*). Pectinate pretarsal claws (state 3) are shaped like a comb, and are only found among ponerines in Leptogenys.

33.2. Mesopretarsal claws, shape: (0) simple, (1) basal tooth, (2) pre-apical tooth, (3) pectinate.
Definition analogous to that of char. 33.1.

33.3. Metapretarsal claws, shape: (0) simple, (1) basal tooth, (2) pre-apical tooth, (3) pectinate.
Definition analogous to that of char. 33.1.

34. Pretarsal claws, arolium (modified from Keller 2011, char. 83): (0) indistinct, (1) distinct.
If distinct (state 1), the arolium is conspicuous and clearly visible under a stereomicroscope (as in *Simopelta oculata*). If indistinct (state 0), it is absent or reduced to a membranous cuticular flap between the pretarsal claws, and not visible under a stereomicroscope (as in *Pachycondyla procidua*).

37. Petiolar tergite, laterotergite (as in Keller 2011, char. 101): (0) indistinct, (1) distinct.

39. Petiolar poststernite, posterior spatulate projection (modified from Keller 2011, char. 111): (0) absent, (1) present.
When present, the spatulate projection rises from the posterior area of the petiolar poststernite and extends posteriad as a broad, blunt prominence, overlapping either partially or entirely the remaining sternite (as in *Asphinctopone silvestrii*).

40. Abdominal presclerites III (helcium), position (as in Keller 2011, char. 114): (0) infraaxial, (1) axial.

41. Abdominal poststernite III, prora: (0) absent, (1) present and conspicuous, (2) present but indistinct in the profile of undissected specimens.
The prora is an anteroventral process of abdominal sternite III that contacts the petiole sternite and gives stability to the ventral flexion of the gaster during stinging (Boudinot et al. 2020). If present, it is located anywhere from the area between the ventral margins of the helcial tergite arch to the anterior surface of the poststernite of abdominal segment III. The prora may be well developed (as in *Corrieopone nouragues*), or a poorly developed protrusion (as in *Cryptopone guianensis*) visible in the profile of undissected specimens (state 1). Alternatively, it may be a minute prominence restricted to the area between the ventral margins of the helcial tergite, and can only be visualized in ventral view or when the gaster is disassociated from the petiole (state 2, as in *Brachyponera croceicornis*). If absent (state 0), protrusions of any shape and size are absent from the area between the ventral margins of the helcial tergite and the anterior surface of abdominal sternite III.

43. Abdominal segment IV, constriction between presclerites and postsclerites (as in Keller 2011, char. 121): (0) absent, (1) present.

44. Abdominal pretergite IV, stridulitrum (as in Keller 2011, char. 124): (0) absent, (1) present.

46.1. Abdominal sternite VII (hypopygium), ventral face: (0) convex or flat, (1) concave.
In *Corrieopone*, the ventral face of the hypopygium is conspicuously concave longitudinally. This is likely an autapomorphy of the genus.

46.2. Abdominal sternite VII (hypopygium), hook-shaped setae on ventroposterior portion: (0) absent, (1) present.
The ventroposterior surface of the hypopygium in *Corrieopone* bears longer, stout, hook-shaped setae which are conspicuous in profile. This is likely an autapomorphy of the genus.

47. Abdominal sternite VII (hypopygium), stout setae/microtrichia on posterolateral portion: (0) absent, (1) present.
If present, the posterolateral portion of the hypopygium bears spine-like microtrichia (as in *Thaumatomyrmex fraxini*), or stout, spine-like, or aristate setae (as in *Pachycondyla crassinoda*) which may be conspicuous or minute.

References

Boudinot BE, Perrichot V, Chaul JCM (2020) Camelosphecia gen. nov., lost ant-wasp intermediates from the mid-Cretaceous (Hymenoptera, Formicoidea). ZooKeys 1005: 21–55. https://doi.org/10.3897/zookeys.1005.57629

Fisher BL, Bolton B (2016) Ants of Africa and Madagascar: A guide to the genera. University of California Press, Berkeley, 514 pp.

Keller RA (2011) A phylogenetic analysis of ant morphology (Hymenoptera: Formicidae) with special reference to the poneromorph subfamilies. Bulletin of the American Museum of Natural History 355: 1–90. https://doi.org/10.1206/355.1
